# Quantifying the Uncertainty of Force Field Selection on Adsorption Predictions in MOFs

**DOI:** 10.1021/acs.jctc.4c00287

**Published:** 2024-05-31

**Authors:** Connaire McCready, Kristina Sladekova, Stuart Conroy, José R. B. Gomes, Ashleigh J. Fletcher, Miguel Jorge

**Affiliations:** † Department of Chemical and Process Engineering, University of Strathclyde, 75 Montrose Street, Glasgow G1 1XJ, United Kingdom; ‡ CICECO − Aveiro Institute of Materials, Department of Chemistry, University of Aveiro, Campus Universitário de Santiago, Aveiro 3810-193, Portugal

## Abstract

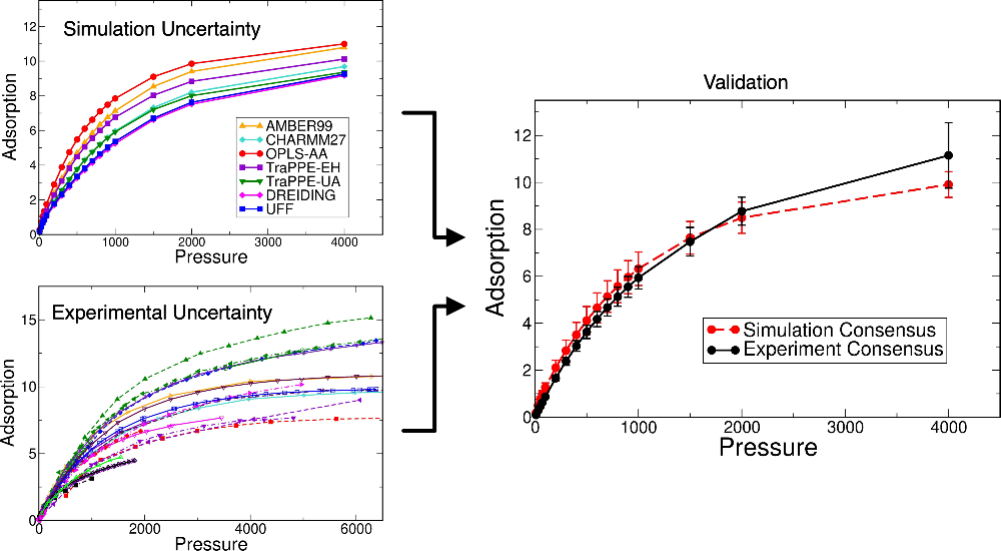

Comparisons between simulated and experimental adsorption isotherms in MOFs are fraught with challenges. On the experimental side, there is significant variation between isotherms measured on the same system, with a significant percentage (∼20%) of published data being considered outliers. On the simulation side, force fields are often chosen “off-the-shelf” with little or no validation. The effect of this choice on the reliability of simulated adsorption predictions has not yet been rigorously quantified. In this work, we fill this gap by systematically quantifying the uncertainty arising from force field selection on adsorption isotherm predictions. We choose methane adsorption, where electrostatic interactions are negligible, to independently study the effect of the framework Lennard–Jones parameters on a series of prototypical materials that represent the most widely studied MOF “families”. Using this information, we compute an adsorption “consensus isotherm” from simulations, including a quantification of uncertainty, and compare it against a manually curated set of experimental data from the literature. By considering many experimental isotherms measured by different groups and eliminating outliers in the data using statistical analysis, we conduct a rigorous comparison that avoids the pitfalls of the standard approach of comparing simulation predictions to a single experimental data set. Our results show that (1) the uncertainty in simulated isotherms can be as large as 15% and (2) standard force fields can provide reliable predictions for some systems but can fail dramatically for others, highlighting systematic shortcomings in those models. Based on this, we offer recommendations for future simulation studies of adsorption, including high-throughput computational screening of MOFs.

## 1 Introduction

Metal–organic frameworks (MOFs) are crystalline solid materials that consist of inorganic nodes (either metal ions or secondary-building units (SBUs) based on metal-containing clusters) coordinatively bonded to organic ligands (linkers) in a three-dimensional porous network. They have been gaining increasing interest from researchers in recent years, with the field growing rapidly since the 1990s.^1^ Greater pore volumes and surface areas are some of the many advantages MOFs hold over more conventional adsorbent materials such as activated carbons and zeolites.^1^ They are also highly tunable, allowing researchers to, at least in principle, tailor their properties (e.g., pore size distribution, chemical functionality) by judiciously combining different metal centers and organic linkers. These desirable characteristics have led to a wide variety of potential applications, such as for biomedicine, catalysis for organic reactions, even radiation detection and chemical sensors, and, most notably, adsorption-based processes like gas separation and storage, leading to a veritable explosion in research on this class of materials.^2^ Currently, over 60,000–70,000 MOF structures are already listed in the Cambridge Crystallographic Database (CCD),^3^ and more are expected given the development of computational software to identify new hypothetical MOFs.^4,5^ There are far too many materials to feasibly study through systematic laboratory experiments, but computational modeling, such as Grand-Canonical Monte Carlo (GCMC), can screen these systems more time- and cost-effectively. Computational screening of MOFs for adsorption-based applications, such as gas storage and separation, heavily relies on simulations to accurately describe the structural and chemical properties, as well as the adsorption mechanism. The accuracy, and therefore predictive ability, of the molecular simulation can be very sensitive to the model parameters used for the adsorbate–adsorbent interactions.^6,7^

The most commonly used force fields when modeling adsorption in MOFs are the Universal Force Field (UFF)^8^ and DREIDING.^9^ Their development stemmed from the desire for a generalized set of Lennard–Jones (LJ) parameters that could cover as many chemical elements as possible (including metal atoms) rather than focusing development on a smaller subset of atoms, such as those from proteins, organics, and nucleic acids, as done in many popular force fields like AMBER,^10^ CHARMM,^11^ and OPLS.^12,13^ DREIDING was published first, but its limited coverage of inorganic atoms led to the development of UFF, which provided parameters for the full periodic table. The presence of various metal sites in MOF frameworks meant that UFF and DREIDING were a natural choice for pioneering studies of molecular simulation of adsorption in MOFs. Indeed, the very first studies of this kind made use of those force fields for the entire MOF framework; see Kawakami et al.^14^ and Vishnyakov et al.^15^ who used UFF and Sarkisov et al.^16^ who used DREIDING. The relatively good agreement with the limited experimental data available at the time, coupled with the convenience of generic force fields that covered a wide range of chemical elements, led to the almost universal adoption of UFF and DREIDING (or combinations thereof) for molecular simulations in MOFs. However, it has become apparent over the years that UFF and DREIDING may not always accurately describe the underlying intermolecular interactions.^17−20^

Despite this fact, the speed of developments in MOF research, coupled with the inherent challenges in developing and testing robust force fields for adsorption systems,^21,22^ means that force field parameters are still generally taken “off the shelf” from the literature with, at best, limited validation—i.e., comparison against a single experimental adsorption isotherm—and often with no validation at all. When discrepancies between simulation predictions and experimental data emerge, the most commonly adopted approach has been to adjust the force field parameters,^23−26^ often to match a single (or a very limited set of) adsorption isotherm(s). However, this can lead to rather disastrous results. For example, Yang and Zhong^24^ adjusted the parameters of the OPLS-AA force field to match an experimental hydrogen adsorption isotherm covering a relatively low-pressure range. When data at higher pressures later became available, it was shown that the adjusted model failed to capture the behavior at high pressure and greatly overpredicted adsorption uptake compared to experimental results.^27^ Although this is just an anecdotal example, it highlights the pitfalls of “blindly” adjusting force field parameters to match a limited set of experimental data. Such an approach often lacks a physical basis, for example, when attempting to describe coordination-type interactions at open metal sites by adjusting dispersion interaction parameters,^22^ and overlooks the inherent uncertainty in experimental measurements of adsorption in MOFs.^28−30^

The reproducibility and reliability of reported experimental isotherms can be seriously lacking, thus leading to uncertainty when comparing a simulated adsorption isotherm or one’s own experimental results to a previously published experimental adsorption isotherm. Park et al. concluded in 2017 that, for CO_2_ adsorption isotherms, only 15 of the thousands of known MOFs contained reproducible experimental adsorption isotherms (i.e., where independent research groups obtained consistent results on the same system).^31^ Furthermore, they reported that ∼20% of the CO_2_ adsorption isotherms they analyzed were outliers and hence likely to constitute erroneous measurements or data obtained on material samples of poor quality. Similar observations were later made on adsorption isotherms for alcohols^32^ and alkanes,^33^ suggesting that this may be a general phenomenon. Furthermore, poor reproducibility is likely an even greater problem for binary adsorption experiments.^34^

Another issue concerns the lack of detail regarding the experimental procedures and/or the characterization of the MOF materials. Even values as significant as the BET surface area and pore volume fail to be reported in several publications for both experimental and simulated systems. An important consequence of this situation is that one should avoid using a single experimental adsorption isotherm when comparing with simulations and should instead try to find as many consistent isotherms as possible for the chosen system. In this context, the NIST-ISODB database provides a very useful resource.^35^ Recently, efforts have been made to match MOF isotherms from the NIST-ISODB with their corresponding structures in the Cambridge Structural Database (CSD).^36^ Ongari et al. found that only 35% of the measured pore volumes fell within 75–110% of the theoretical geometric pore volume. For the remaining systems, it is likely that deviations from the theoretical crystal structure in the MOF sample, such as collapsed pores or unremoved solvent, prevent a direct comparison between the experimental uptake and molecular simulation predictions.^36^

To our knowledge, despite the extremely large number of GCMC studies of adsorption in MOFs, there have only been limited attempts at a systematic assessment of the effect of force field choice on the accuracy of the results.^7,17,18,37−39^ Those studies were mostly restricted to either a single MOF (or family of MOFs) and/or to a limited number of force fields. For example, the recently developed CRAFTED database of simulated adsorption isotherms considers only UFF and DREIDING.^7^ We aim to fill this gap by systematically investigating the impact of varying framework force field parameters on adsorption isotherm predictions, attempting to address several fundamental questions in the process: (i) How likely is a particular generic force field (like UFF or DREIDING) to accurately predict experimental adsorption in MOFs? (ii) Does any generic force field emerge as a more reliable choice for adsorption predictions? (iii) How can we quantify the uncertainty arising from force field selection? (iv) What are the consequences of comparing a single simulation model against a single experimental isotherm (i.e., the conventional approach)? (v) Should force field parameters be “tweaked” to match limited experimental adsorption data?

Following on from our recent study on the impact of choosing different point charge assignment methods on adsorption isotherm predictions in MOFs,^40^ here we vary only the framework Lennard–Jones (LJ) parameters. To minimize the effect of point charges and the impact of electrostatic interactions, we select a system—methane adsorption using a United Atom (UA) model^41^—where we can safely ignore them and treat the frameworks as electronically neutral. The adsorbate model was kept constant throughout all our simulations because our focus was to test the effect of the framework LJ force field parameters on adsorption predictions in isolation. As test systems, we chose Cu-BTC (also known as HKUST-1),^42^ IRMOF-1 (also known as MOF-5),^43^ Co-MOF-74 (also known as Co-DOBDC),^44^ MIL-47,^45^ and UiO-66,^46^ thus covering a variety of MOFs from distinct “families”. Most of these systems are well-researched, with plenty of published data that can be mined/analyzed. However, others, particularly MIL-47, are less extensively covered and required extra effort to obtain appropriate data. We adopt an experimental data collection and curation process similar to that of Sholl and co-workers^31−33^ but scale experimental isotherms by the ratio of the theoretical to experimental pore volume (see details and discussion below) to account for potential sample imperfections.^28^ This allows us to generate consensus isotherms that include an estimate of experimental uncertainty, which are then used to assess the suitability of each force field for predicting adsorption. Such a systematic comparison is hitherto unprecedented and shows that good predictions from “off-the-shelf” force fields should not be taken for granted even for such a simple adsorbate as methane.

## 2 Methodology

### 2.1 Experimental Data Collection and Curation

For each MOF considered here, we followed the procedure below:

1. Collect data for methane at *T* = 298 ± 5 K from NIST-ISODB and from supplementary literature search, if necessary.
2. Categorize isotherms by color code with respect to the reporting of sample pore volume and/or N_2_ adsorption isotherm at 77 K on the same sample.
3. Discard isotherms with no reported information for pore volume calculation.
4. Scale each experimental adsorption isotherm by the ratio of the theoretical and experimental pore volumes.
5. Fit each scaled adsorption isotherm to the Toth isotherm model.
6. Recalculate isotherms using Toth model parameters at a predefined uniform set of pressures.
7. Apply Tukey’s method to identify and discard outliers.
8. Calculate average adsorption uptake over all isotherms, and a 95% confidence interval error bar for each pressure point.

Following this procedure yields an experimental “consensus isotherm” with uncertainty for each MOF, which can be compared against molecular simulation predictions. These are analogous to those reported by Sholl and co-workers,^31−33^ with a few important differences that we discuss below. In Supporting Information (Section 1), we describe each step of the above procedure in more detail using Cu-BTC as an example. We also include detailed spreadsheets containing all the collected experimental isotherms and subsequent analysis as additional information (see link under “Data Availability”).

As explained above, our study focused on methane isotherms measured at 298 K but allowed for a variation of ±5 K, in line with the approach of Park et al.^31^ The starting point for our data collection was the NIST/ARPA-E Database of Novel and Emerging Adsorbent Materials (NIST-ISODB).^35^ Every isotherm collected from the NIST-ISODB was checked against the original reference and redigitized if necessary (e.g., points were too sparse, incorrect units, incorrect pressure scale). We also identified a few instances where the original source of the data set did not correspond to the DOI reported in the NIST-ISODB; in such cases, we checked the NIST-ISODB for accuracy against the original source of the experimental measurements. The data collection from the NIST-ISODB was supplemented by a manual literature search; we used Clarivate’s Web of Science with keywords “(methane OR CH4) AND (MOF)”, where “(MOF)” refers to the material name, including synonyms where relevant.^35^ This was required for all MOFs studied here except for Cu-BTC due to a shortage of viable isotherms. This allowed us to collect and analyze a larger number of isotherms than in the recent study of Bingel et al.,^33^ which included methane adsorption on three of our five selected MOFs with overlapping temperature ranges but only considered data available in the NIST-ISODB (see Table 1). Note, however, that we cannot guarantee that *all* relevant methane isotherms thus published in the scientific literature have been collected and analyzed. As shown in Figure S16, our consensus isotherms (before pore volume scaling) are statistically consistent with those reported by Bingel et al. for the three MOFs that were considered in both studies, which support the robustness of this procedure.

**Table 1:** Summary of the Number of Isotherms Analyzed for Each MOF^a^

**MOF**	***N*_A_**	***N***_**A**_^′^	***N*_B_**	***N***_**B**_^′^	*T*_B_**(K)**
Cu-BTC	27	15	31	26	303 ± 5
IRMOF-1	44	12	10	9	298 ± 5
UiO-66	46	33	11	10	303 ± 5
MIL-47	13	3			
Co-MOF-74	8	5			

a *N_i_* is the total number of collected isotherms, *N*_*i*_^′^ is the final number of consistent isotherms, the subscript A represents this work, and B represents that of Bingel et al.^33^ We also report the temperature ranges (*T*_B_) in the work of Bingel et al. because they were not always the same as used here, i.e.298 ± 5 K.

One of the pitfalls of comparing molecular simulations against experimental isotherms is that the former are, most often at least, carried out on perfect crystal structures, whereas the latter are measured on inherently “imperfect” samples due to, e.g., defects, impurities, and incomplete activation. One possible way in which these effects can be mitigated is to scale the experimental data by the surface area (SA) or pore volume (*v*_p_), i.e., multiply them by the ratio of the theoretical SA or *v*_*p*_ to the corresponding property of the experimental sample to enable direct comparison against simulation data on a perfect crystal. In this work, we chose to use the ratio of pore volumes rather than surface areas because calculating the latter in microporous materials, such as MOFs, is fraught with reproducibility problems and questionable assumptions, as demonstrated in recent work.^47,48^

To maximize consistency in the determination of the experimental pore volumes, we also collected experimental N_2_ (77 K) or Ar (87 K) isotherms on the MOF samples corresponding to each experimental methane isotherm when such data were available (isotherms categorized as “green”; see SI for details). We then calculated the pore volume using the Gurvitsch rule,^49^ which has been shown to agree with geometric pore volume calculations on many microporous materials despite the approximations involved.^50^ The N_2_/Ar uptake at saturation was estimated by carrying out a linear least-squares fit of the plateau region of the adsorption isotherm and interpolating or extrapolating to *P*/*P*_Sat_ = 0.99 (see Figure S3 for a detailed example calculation). The theoretical pore volume was calculated using the same procedure, ensuring that all samples were treated consistently, regardless of the quality and range of the underlying data. We note that somewhat different procedures have been used by other authors—for example, Ongari et al.^36^ took the average of the reported uptakes over a range of *P*/*P*_Sat_ between 0.6 and 0.8—and we also tested the impact of this alternative approach (see Section 3.1).

If N_2_/Ar isotherms were unavailable but the authors reported a value for the sample pore volume, the methane isotherms were still considered and scaled using that reported value (isotherms categorized as “amber”). This is not ideal because we cannot ensure that the reported pore volume was calculated in a way that is consistent with the above approach. However, it is likely to have a marginal impact on the results because only 24% of all collected isotherms were classified as amber, compared to 64% classified as green. Finally, when no information about the pore volume was provided (isotherms categorized as “red”), the corresponding methane isotherms were discarded from further analysis; 12% of all collected isotherms fell under this category. Figure S2 shows an example of this color coding for Cu-BTC.

The application of pore volume scaling is the most important difference between our procedure and that of Sholl and co-workers.^31−33^ The underlying assumption is that the real sample can be approximated as a mixture of pure MOF crystal and a nonadsorbing component that contributes only to the sample mass but not to the adsorbed amount. For this assumption to be valid, the experimental pore volume should always be lower than the theoretical one, but not by a very large amount as this would suggest a more extensive level of defects of a different nature. This was indeed observed for the vast majority of isotherms collected on Cu-BTC, IRMOF-1, Co-MOF-74, and MIL-47, in agreement with the results of Ongari et al.^36^ For UiO-66, however, most samples had pore volumes that exceeded the theoretical estimate, sometimes by as much as 50%. This clearly suggests that UiO-66 samples are highly defective and do not align with the simple approximation described above. We will return to this point in Section 3.5.

**Figure 1:**
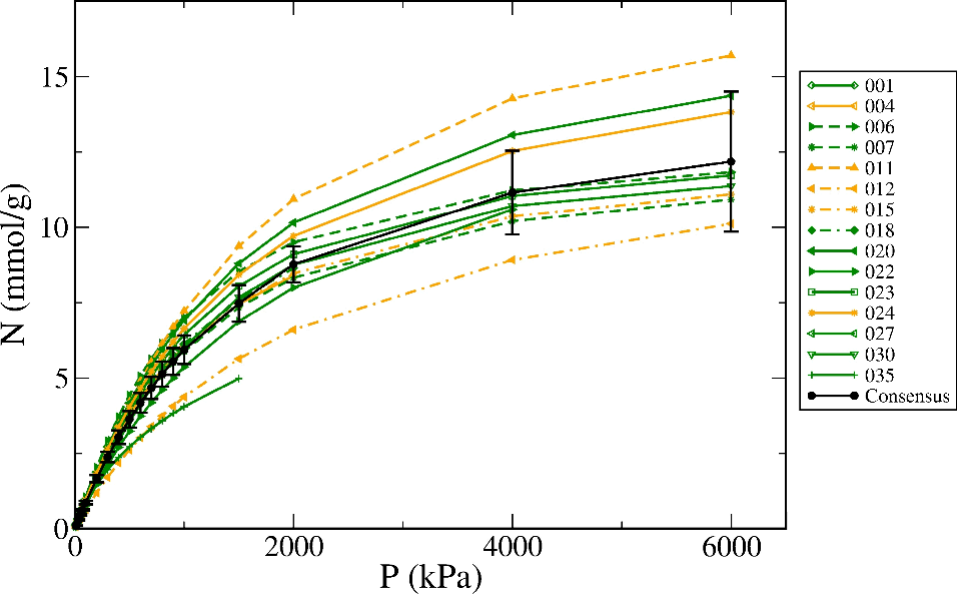
Isotherms collected from the NIST-ISODB for methane on Cu-BTC at 298 ± 5 K after scaling by the pore volume ratio, fitting to the Toth isotherm model, and removing outliers. Also shown is the consensus isotherm with error bars (black line). The labels in the legend correspond to individual entries in the data spreadsheets provided as additional information.

The final step of our procedure is to identify and remove outliers, which was achieved by applying Tukey’s method^51^ to the data for each pressure point. Overall, 13% of green and amber isotherms were marked as outliers, in good agreement with the estimate of ∼15% obtained by Bingel et al.^33^ The remaining isotherms were used to calculate an average of the experimental data and the 95% confidence interval error bars for each pressure point, as shown in Figure 1 for the case of Cu-BTC.

### 2.2 Simulation Details

We simulated pure methane on Cu-BTC (HKUST-1),^42^ IRMOF-1 (MOF-5),^52^ Co-MOF-74 (Co-DOBDC),^44^ MIL-47,^45^ and UiO-66 (dehydroxylated form).^46^ All modeled adsorption isotherms were calculated by GCMC simulations using RASPA 2.0.47.^53^ The simulations were run with sufficient equilibration and sampling steps to ensure precise calculations of the adsorbed amount at each temperature and pressure; namely, we ran 1 × 10^5^ initializing cycles and 2 × 10^5^ sampling cycles at each pressure, corresponding to the experimental data (see Section 2.1). Potentials were truncated at a cutoff radius of 11.0 Å and tail corrections were employed. Although not always employed when simulating adsorption systems, LJ tail corrections have been shown to virtually eliminate the dependence of simulated isotherms on the cutoff radius.^38,54^ In Figure S17, we confirm that our simulated isotherms are indeed independent of the choice of cutoff radius when tail corrections are used. MC trials were accepted or rejected based on a probability that is proportional to their Boltzmann factor, with TranslationProbability and SwapProbability weighted at a ratio of 1:2 (i.e., translation, insertion, and deletion trials are equally weighted).

The Lennard–Jones 12–6 potential and Lorentz–Berthelot combining rules described all dispersion and repulsion interactions. The methane adsorbate was modeled using TraPPE-UA^41^ in all our simulations, which has been shown to describe the vapor–liquid equilibrium curves of alkanes very accurately.^41^ Atoms in the MOF framework were assigned model parameters from seven different force fields: AMBER-99,^10^ CHARMM-27,^11^ OPLS-AA (AA = All-Atom),^12,13^ TraPPE-UA,^55^ TraPPE-EH (EH = Explicit Hydrogens),^56^ UFF,^8^ and DREIDING.^9^ Because most of these force fields do not include parameters for metal atoms (the exceptions are UFF and, to a much more limited extent, DREIDING), we opted to assign the same UFF force field parameters for metal atoms in all MOF/force field combinations. This means that, strictly speaking, our study assesses the effect of force field parameters for nonmetal atoms. The implications of this assumption will be discussed later. All MOF structures were assumed to be rigid and taken from the RASPA GitHub repository (https://github.com/iraspa/RASPA2).

Apart from methane, we also carried out simulations of N_2_ adsorption at 77 K for the purpose of calculating theoretical pore volumes necessary for scaling the experimental isotherms (see Section 2.1 for details). In those runs, nitrogen was modeled by the TraPPE force field,^57^ which contains point charges to better describe the small quadrupole moment of that molecule. Point charges for the framework atoms were obtained from DDEC6 calculations,^58−60^ and Ewald summations^61−63^ were employed when calculating the electrostatic interactions.

**Figure 2:**
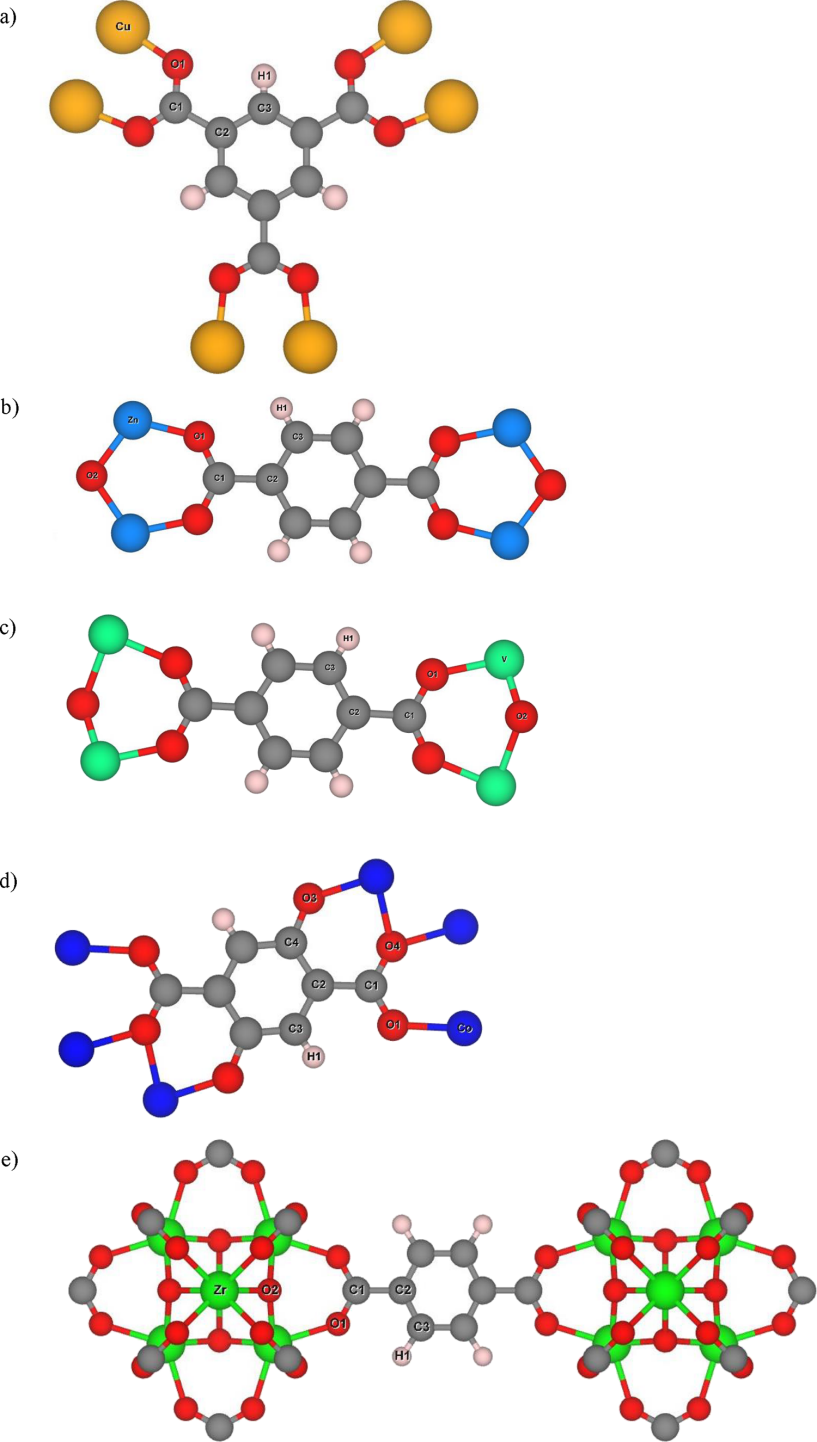
Repeating units with labeled unique atom types for all MOFs studied here: (a) Cu-BTC, (b) IRMOF-1, (c) MIL-47, (d) Co-MOF-74, and (e) UiO-66.

To assign framework LJ parameters, first, the repeating unit for each MOF was isolated, and its unique atom types were labeled (Figure 2). When assigning force field parameters, the main challenge is that none of the general force fields considered above have been developed for MOFs (or, indeed, for any hybrid organic–inorganic material). When assigning parameters, we tried to identify the force field atom types corresponding to the most similar chemical environment observed in the MOF unit cell. For some force fields, more than one choice was possible, in which case we carried out a sensitivity analysis to assess the variability arising from atom type assignment. Below, we describe this process in detail, again using Cu-BTC as an example.

For DREIDING and UFF, the selection of ε and σ parameters was straightforward due to their generalized approach of assigning a single set of parameters for each chemical element. Therefore, every carbon atom in the MOF repeating units was assigned the same ε and σ values, even though the chemical environment of those carbon atoms can be quite distinct. The other four force fields considered here were developed with organic molecules in mind—either trying to describe small organic molecules, like in TraPPE and OPLS, or focusing more closely on biomolecules, like in AMBER and CHARMM. As such, they allow for a greater degree of flexibility in the parameter assignment.

**Figure 3:**
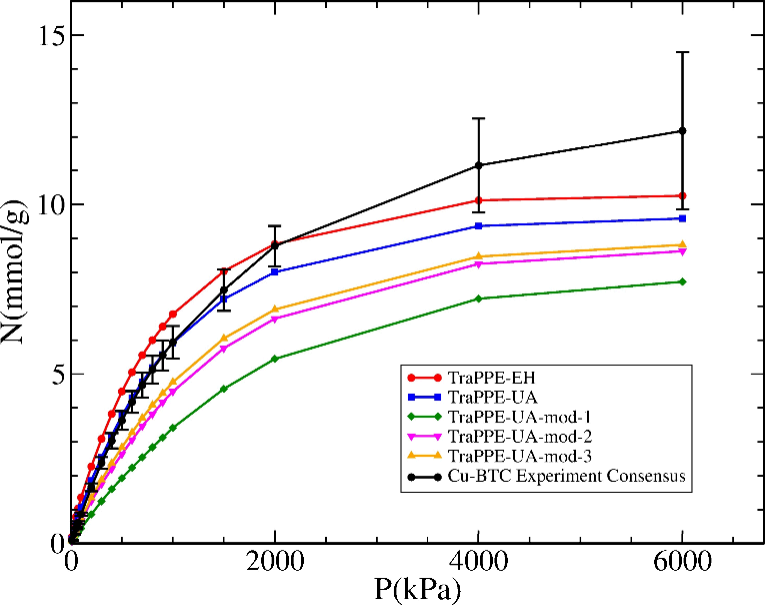
Effect of changing the parameter assignments of the O and C1 atoms using TraPPE-EH and TraPPE-UA on the predicted adsorption isotherms of methane on Cu-BTC (see text for a description of each parameter set). Also shown is the consensus experimental isotherm for Cu-BTC (black circles).

The TraPPE force fields allowed for a distinction between the different carbon atoms. Two sets of parameters that rationally described the chemistry of the repeating unit were used: one each from the TraPPE-EH^55^ and TraPPE-UA^56^ force fields. In both cases, the oxygen atom type was described as a double-bonded oxygen in an ester functional group^64^ because it is the most similar chemical environment to the carboxylate moiety present in many MOFs (see Figure 2). We note that TraPPE assigns the same LJ parameters to oxygen atoms in carbonyl (e.g., ketones and aldehydes) and carboxyl (e.g., esters and carboxylic acids) groups. This logically led to C1 being allocated as a carbon double-bonded to oxygen from an ester group,^64^ which uses the same parameters as in carboxylic acids.^65^ The difference between the two parameter sets derives from the possibility of describing the aromatic ring section of the repeating unit with parameters from either an explicit-hydrogen benzene model or a united-atom model used for toluene. We recall that in the UA approach, the effect of nonpolar hydrogen atoms is accounted for implicitly in the ε and σ parameters of the adjacent carbon; i.e., each CH_*x*_ group is described as a single interaction site. As such, the parameters for C2 and C3 in TraPPE-UA are different because the latter must implicitly include the effect of the adjacent hydrogen atom. In contrast, both carbons are assigned the same parameters in TraPPE-EH due to the explicit treatment of nonpolar hydrogens in that force field. As we can see in Figure 3, there is a relatively small difference in the methane adsorption isotherms predicted by those two models, with TraPPE-EH leading to slightly higher adsorbed amounts throughout the whole pressure range. The consensus experimental isotherm for Cu-BTC is close to the two TraPPE model predictions, with a better agreement with TraPPE-UA at low pressures, and a somewhat better agreement with TraPPE-EH at high pressures. We carry out a more detailed comparison between simulations and experiments in Section 3.3.

Although we believe that our parameter selection, described above, provides the most chemically realistic description of the environment of each atom, alternative assignments have been used in the past. Namely, Lyubchyk et al.^66^ used the TraPPE-UA model for the aromatic ring carbons with an identical parameter assignment as we described above, but assigned ester/ether single-bonded oxygen parameters^67^ to the linker oxygen atoms and sp^2^ alkene carbon parameters to the C1 atom.^56^ This parameter set is presented as TraPPE-UA-mod-1 in Figure 3 (see Table S1 for parameters). To analyze the effect of each of those assignments in more detail, we have also calculated isotherms where only the oxygen atom was changed (TraPPE-UA-mod-2), and where only the carbon atom was changed (TraPPE-UA-mod-3) to the assignment of Lyubchyk et al. Both changes lead to an underestimation of the adsorbed amounts when compared to the base TraPPE-UA but greater uptake compared to TraPPE-UA-mod-1. As we can see, the differences are quite significant, and all the alternative parameter assignments lead to a substantial underestimation of the experimental isotherm. This emphasizes the need for a careful and consistent assignment of atom type parameters.

For the remaining force fields, we strived to ensure chemical consistency in our final parameter assignment while carrying out a similar analysis as shown in Figure 3 to test the sensitivity of resulting isotherms to atom type choices (see the Supporting Information for details). In brief, for AMBER-99, all carbon atoms were assigned the same LJ parameters corresponding to carbonyl and pure aromatic carbons because this force field does not distinguish between those two atom types, whereas the hydrogen was designated as aromatic. The oxygen was assigned as a carbonyl/carboxyl (i.e., double-bonded) atom type, but the impact of assigning parameters for an ester/ether (i.e., single-bonded) oxygen atom type was found to be relatively minor (Figure S18a). In CHARMM-27, we assigned carbonyl/carboxyl carbon parameters to C1 and aromatic carbon parameters to C2 and C3 because the force field allowed for this distinction. O1 was assigned carbonyl/carboxyl oxygen parameters, and again, the impact of an alternative selection was minor, although, curiously, it was in the opposite direction to that observed for AMBER-99 and TraPPE-UA (Figure S18a). Finally, in OPLS-AA, C1 was described as a carboxylate/ester carbon double-bonded to oxygen, whereas C2/C3 and H were assigned aromatic atom types. We tested three options for oxygen: carboxyl/ester double-bonded oxygen, hydroxyl/ester single-bonded oxygen, and ether oxygen (Figure S18b). We opted for the former assignment for chemical consistency. A full list of the various parameters for all force fields and modifications thereof is provided in the Supporting Information (Tables S1–S3).

The atom type assignment for the remaining MOFs followed the same logic as for Cu-BTC. However, there is an additional atom type for the oxygen atoms that sit within the metal cluster and are only coordinatively bonded to metal atoms, labeled as O2 in Figure 2. After testing various oxygen parameters, the O2 atom was eventually allocated as an oxygen from a hydroxyl group. This made logical sense because those oxygen atoms normally originate from hydroxyl groups during MOF synthesis reactions.^68^ Furthermore, the impact of changing the atom type assignment for those oxygen atoms on the simulated adsorption isotherm was found to be negligible (see Figure S19). This assignment completes the framework model for IRMOF-1 and MIL-47 materials.

UiO-66 has the same organic linker as IRMOF-1 and MIL-47 but has a somewhat more complex metal cluster because it can be present in either a hydroxylated (Zr_6_O_4_(OH)_4_ SBU) or dehydroxylated/dehydrated (Zr_6_O_6_) form.^69^ The UiO-66 structure from the RASPA GitHub repository is in the dehydroxylated form. It has been shown experimentally that both forms lead to nearly indistinguishable methane adsorption isotherms,^70^ and we also confirmed this through simulations (see Section 3.5). UiO-66 is also prone to defects in its structure, such as missing linkers and/or missing clusters. This phenomenon will be discussed in more detail in Section 3.5, where we simulate methane adsorption in defective UiO-66 structures.

Co-MOF-74 has a repeating unit that is rather different from the other MOFs considered here, with an oxygen atom (O3) bonded to the carbon labeled C4 (Figure 2d) and a cobalt atom. The O3 bonded to C4 was treated as oxygen from a generic hydroxyl group for AMBER-99, CHARMM-27, and OPLS-AA. However, the TraPPE force field distinguishes aliphatic from aromatic hydroxyl groups. Hence, the O3 atom was described as the oxygen from a phenol molecule.^71^ In Co-MOF-74, there are also two distinct carboxylate oxygens: one (O1) coordinated to a single metal atom, as in all the previous MOFs, and another (labeled O4) coordinated to two metal atoms. Although the parameters for O4 were taken as identical to O1 (i.e., it is still a carboxylate atom type), we have labeled it differently to highlight its distinct coordination environment.

Table 2 reports the final selections of LJ parameters from all the force fields used to model each MOF’s inorganic metal and organic ligand sections. We ran GCMC simulations for each model reported in Table 2 and used the predicted isotherms for comparison with the curated experimental data taken from the literature. Input files for all simulations carried out in this work are made openly available through the University of Strathclyde’s data repository (see link under “Data Availability”).

**Table 2:** Lennard–Jones Framework Parameters Used for Modeling the MOFs with Each Force Field Tested Here^a^

**atom**	**UFF**		**DREIDING**		**TraPPE-EH**		**TraPPE-UA**		**AMBER-99**		**OPLS-AA**		**CHARMM-27**	
	**ε**/***k*_B_**	**σ**	**ε**/***k*_B_**	**σ**	**ε**/***k*_B_**	**σ**	**ε**/***k*_B_**	**σ**	**ε**/***k*_B_**	**σ**	**ε**/***k*_B_**	**σ**	**ε**/***k*_B_**	**σ**
Cu^b^	2.516	3.114	2.516	3.114	2.516	3.114	2.516	3.114	2.516	3.114	2.516	3.114	2.516	3.114
Zn^b^	62.399	2.462	62.399	2.462	62.399	2.462	62.399	2.462	62.399	2.462	62.399	2.462	62.399	2.462
V^b^	8.052	2.801	8.052	2.801	8.052	2.801	8.052	2.801	8.052	2.801	8.052	2.801	8.052	2.801
Zr^b^	34.722	2.783	34.722	2.783	34.722	2.783	34.722	2.783	34.722	2.783	34.722	2.783	34.722	2.783
Co^b^	7.045	2.559	7.045	2.559	7.045	2.559	7.045	2.559	7.045	2.559	7.045	2.559	7.045	2.559
O1	30.218	3.118	48.158	3.033	79.0	3.050	79.0	3.050	105.682	2.960	105.682	2.960	60.390	3.029
O2	30.193	3.118	48.158	3.033	93.0	3.020	93.0	3.020	105.883	3.067	85.552	3.070	76.544	3.154
O3	30.193	3.118	48.158	3.033	118.0	3.040	118.0	3.040	105.883	3.067	85.552	3.070	76.544	3.154
O4	30.193	3.118	48.158	3.033	79.0	3.050	79.0	3.050	105.682	2.960	105.682	2.960	60.390	3.029
C1	52.838	3.431	47.845	3.473	41.0	3.900	41.0	3.900	43.279	3.400	52.841	3.750	55.357	3.564
C2	52.838	3.431	47.845	3.473	30.7	3.600	21.0	3.880	43.279	3.400	35.227	3.550	35.227	3.550
C3	52.838	3.431	47.845	3.473	30.7	3.600	50.5	3.695	43.279	3.400	35.227	3.550	35.227	3.550
C4	52.838	3.431	47.846	3.473	30.7	3.600	21.0	3.880	43.279	3.400	35.227	3.550	35.227	3.550
H1	22.142	2.571	7.649	2.846	25.450	2.360	0.0	0.0	7.549	2.600	15.097	2.420	15.097	2.420

a Values for ε/*k*_B_ are in K, and σ is given in Å.

b All parameters for metal atoms were taken from UFF.

## 3 Results and Discussion

A detailed analysis of each of the 5 MOF materials studied here is provided in the Supporting Information. Here, we focus on the main conclusions drawn from our analysis.

### 3.1 Experimental Uncertainty and Pore Volume Scaling

Our statistical analysis of the experimental data yields an estimate of the experimental uncertainty that is ∼12% on average (over all the MOFs and pressure points) but can be as high as 20% in some cases, which is in broad agreement with the conclusions of previous studies.^31−33^ As discussed above, the use of pore volume scaling is the main difference between our experimental data analysis procedure and that of Sholl and co-workers.^31−33^ In Table 3, we show the average scaling factors (i.e., average ratio between the theoretical and experimental pore volumes) for each MOF, as well as the maximum value applied. The scaling factors for Cu-BTC, IRMOF-1, and Co-MOF-74 are only slightly larger than 1. This supports the assumption that the experimental samples are mostly, but not entirely, composed of pure MOF crystal. Note, however, that these scaling factors are averaged for each system after outlier removal; in fact, many of the isotherms identified as outliers had pore volumes that deviated quite dramatically from the theoretical limit (e.g., for IRMOF-1, the three outliers had factors of 4.81, 4.44, and 2.02). For MIL-47 and UiO-66, the scaling factors are generally larger, and these are precisely the materials for which agreement between simulation and experiment is poorer (see Sections 3.4 and 3.5). These observations suggest, in agreement with the analysis of Ongari et al.,^36^ that a comparison between theoretical and experimental pore volumes can help to identify MOF samples of poor quality.

**Table 3:** Average and Maximum Scaling Factors (i.e., the Ratio of Theoretical to Experimental Pore Volume) Applied to the Experimental Data Collected for Each of the Five MOFs Considered Here

	**Cu-BTC**	IRMOF-1	**MIL-47**	**Co-MOF-74**	**UiO-66**^a^
average scaling	1.11	1.18	1.28	1.03	1.30
maximum scaling	1.49	1.37	1.50	1.08	1.82

a The analysis for UiO-66 considered a partially defective sample, as explained in detail in Section 3.5.

**Figure 4:**
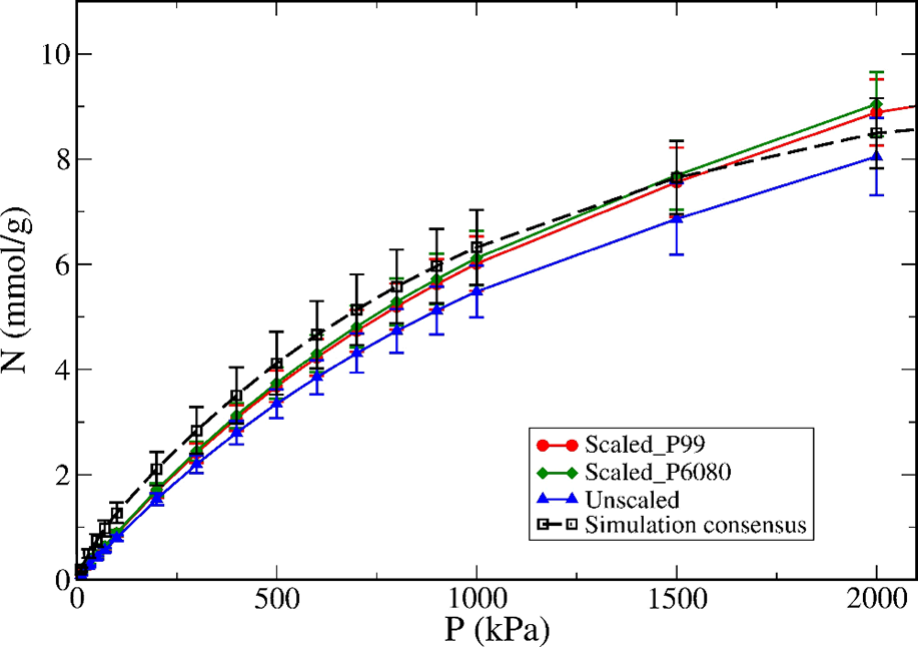
Experimental consensus adsorption isotherms for methane at 298 K on Cu-BTC obtained by averaging the same set of isotherms (i.e., after outlier removal) but using different scaling procedures: red circles, scaled by pore volume determined by extrapolating nitrogen isotherms to *P*/*P*_Sat_ = 0.99; green diamonds, scaled by pore volume determined by averaging nitrogen uptake in *P*/*P*_Sat_ = [0.6–0.8]; blue triangles, without applying pore volume scaling. We also show the simulation consensus isotherm as open black squares and dashed line.

Our analysis found that scaling the experimental isotherms by the pore volume ratio improved agreement between simulation and experimental consensus isotherms. This was the case for four out of five MOFs; the exception was Co-MOF-74, but because of the average scaling factor being very close to 1 (Table 3), the effect of scaling was negligible. An example of this comparison, for Cu-BTC, is shown in Figure 4, where it is clear that the scaled experimental consensus isotherm (red line) is closer to the simulation curve than the unscaled experimental consensus isotherm (blue) over the entire pressure range. We also compare the scaled consensus experimental isotherms obtained using two approaches for the pore volume calculation: (i) extrapolating the amount adsorbed to *P*/*P*_Sat_ = 0.99, as described in Section 2.1, and (ii) averaging the reported uptakes over a range of *P*/*P*_Sat_ between 0.6 and 0.8, as used by Ongari et al.^36^ The two isotherms are practically identical, which suggests that the precise method for estimating the sample pore volume has a negligible effect on the scaling procedure. This is in marked contrast with the surface area, which has been shown to suffer from substantial variability and a strong dependence on the details of the calculation method.^47,48^

### 3.2 Uncertainty Due to Force Field Selection

**Figure 5:**
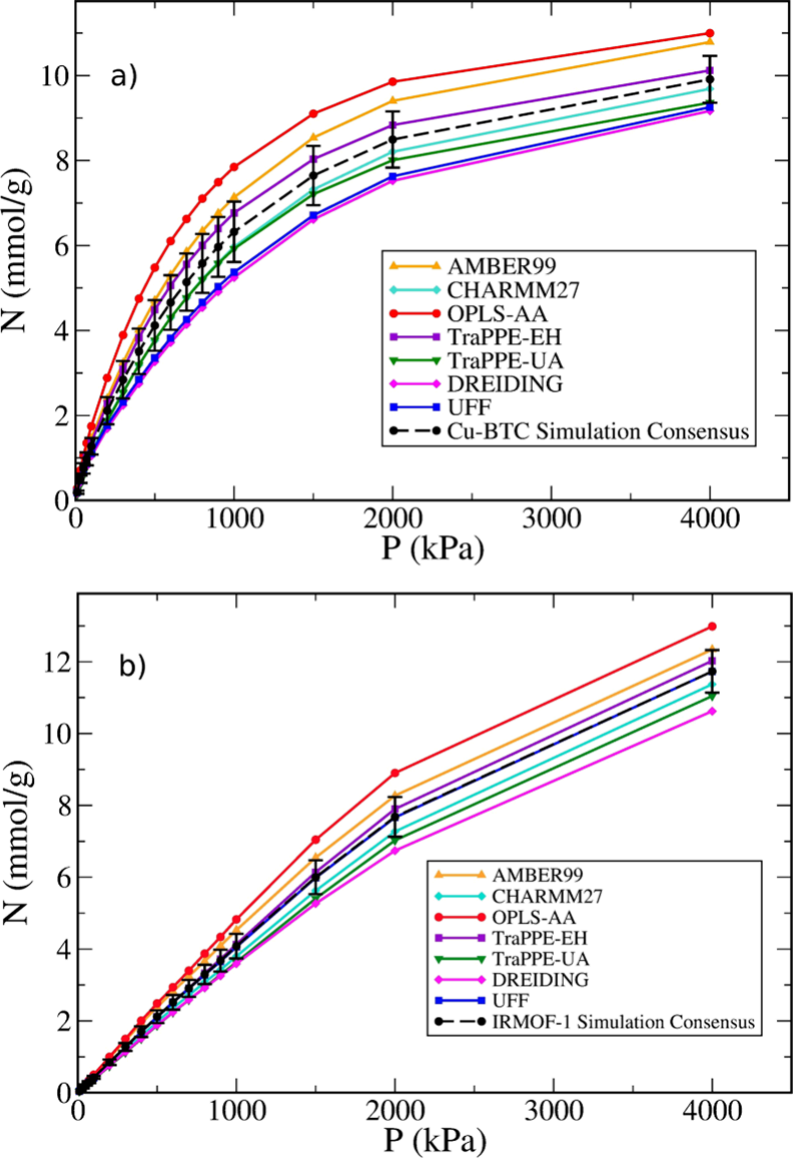
Predicted adsorption isotherms for methane at 298 K on (a) Cu-BTC and (b) IRMOF-1 using different force field parameter sets, together with the average of the simulated isotherms with 95% confidence interval error bars (black circles and dashed line).

One of the main goals of this work is to estimate the uncertainty that arises from the choice of framework force field parameters. In Figure 5, we show simulation results using all force fields for both Cu-BTC and IRMOF-1, which are the MOFs that exhibit the largest and smallest degree of variability, respectively; similar figures for the other MOFs are shown in the Supporting Information. For all MOFs studied here, the simulated isotherms using different force fields show the same general curvature, suggesting that the underlying adsorption mechanism is similar. However, the quantitative differences in adsorbed amount can be quite significant. The average over the seven different models was taken to yield a consensus isotherm for the predicted adsorption uptake of methane (dashed line in Figure 5), and the error bars represent the 95% confidence interval for the average of the simulated data. This uncertainty is 10% on average (over all the MOFs and pressure points) but can be as high as 15% in some cases, which is of the same order of magnitude as the experimental uncertainty (see Section 3.1). It is important to reiterate that this uncertainty arises only from the variation in framework LJ parameters but is already orders of magnitude larger than the statistical uncertainty of individual simulations (which is not visible in Figure 5 because it is smaller than the size of the symbols). It quantifies the potential error that arises when a single force field is chosen off-the-shelf for a molecular simulation study of adsorption in MOFs and, to our knowledge, has not been estimated before.

Although there are slight variations in the ranking of the force fields in terms of total uptake, when analyzing the data for all five MOFs, it is clear that DREIDING is systematically on the low end of the scale; i.e., it predicts the lowest uptake for three of the five MOFs and the second lowest for the other two. Conversely, AMBER-99 and OPLS-AA are systematically on the high end of the scale. This may be related to the rather high ε parameter for the oxygen atoms, particularly for the carboxylate oxygen (O1) in those two force fields (Table 1). The ranking of UFF is rather erratic, predicting the lowest uptake for Co-MOF-74 and the second highest for UiO-66.

To provide a more quantitative assessment of each force field, Table 4 shows the root mean squared deviation (RMSD) over all the isotherm pressure points with respect to the simulation consensus isotherm for each force field/MOF combination; the larger the RMSD is, the more a particular force field deviates from the consensus. Although the results are system-specific, the average RMSD over all MOFs clearly highlights TraPPE-EH as the model that, on average, best approaches the consensus simulation isotherms, followed closely by CHARMM-27. Furthermore, those two force fields predict isotherms that are practically always within the error bars of the consensus isotherm. These results suggest that a single simulation with one of these force fields may present a reasonable alternative to determining a full consensus simulation isotherm in cases where time and/or computational resources are limited, provided the correct uncertainty is reported as well. Conversely, DREIDING and, to a lesser extent, UFF do not appear as the best options if one wishes to accurately replicate the consensus isotherms. This is an important observation if we take into account that the vast majority of simulations of adsorption in MOFs are carried out with those two force fields. Note, however, that this analysis simply identifies which force field is the most *internally consistent* with the set of force fields chosen for analysis; it says nothing about the ability of each force field to predict the correct experimental isotherm. That question is discussed in Section 3.3.

**Table 4:** RMSD Analysis of Individual Force Fields in Relation to the Simulation Consensus^a^

**force field**	**Cu-BTC**	IRMOF-1	**MIL-47**	**Co-MOF-74**	**UiO-66**	**average RMSD**
AMBER-99	0.600	0.335	0.681	0.663	0.646	0.585
CHARMM-27	**0.278**	0.210	0.266	0.167	0.403	0.265
OPLS-AA	1.138	0.604	0.542	0.474	0.402	0.632
TraPPE-EH	0.302	0.096	**0.076**	**0.127**	0.454	**0.211**
TraPPE-UA	0.339	0.349	0.974	0.146	0.405	0.443
DREIDING	0.757	0.455	0.267	0.416	0.489	0.477
UFF	0.669	**0.012**	0.254	0.535	**0.393**	0.373

a The force field with the lowest RMSD is highlighted in bold for each system.

### 3.3 Simulation vs Experimental Consensus

**Figure 6:**
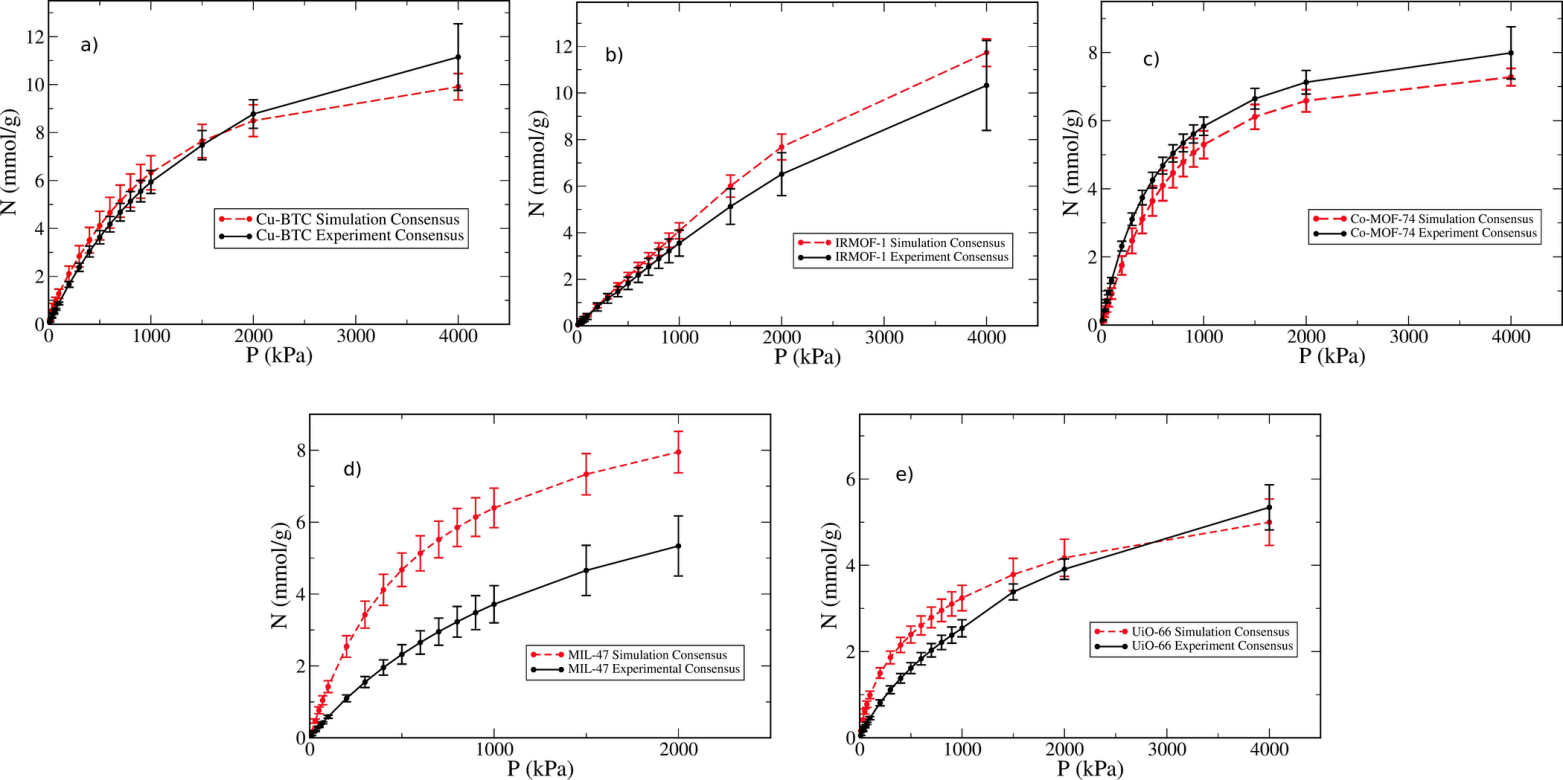
Experimental and simulation consensus isotherms for methane at 298 K on (a) Cu-BTC, (b) IRMOF-1, (c) Co-MOF-74, (d) MIL-47, and (e) UiO-66. Error bars represent the 95% confidence interval of the mean.

Having completed the statistical analysis of both experimental and simulation data, we are now in a position to carry out a comparison of the consensus isotherms, rigorously taking into account their respective uncertainties. Figure 6 shows this comparison for all five MOFs studied here. The experimental and simulation consensus isotherms overlap within their respective error bars for three out of the five MOFs (Cu-BTC, IRMOF-1, and Co-MOF-74). The good agreement observed for Cu-BTC and Co-MOF-74, both of which contain open metal sites (OMS) in their structure, strongly suggests that the effect of OMS on methane adsorption at room temperature is relatively small. Indeed, we observed this to be the case for both ethane and propane in previous work.^28,29,72^

**Figure 7:**
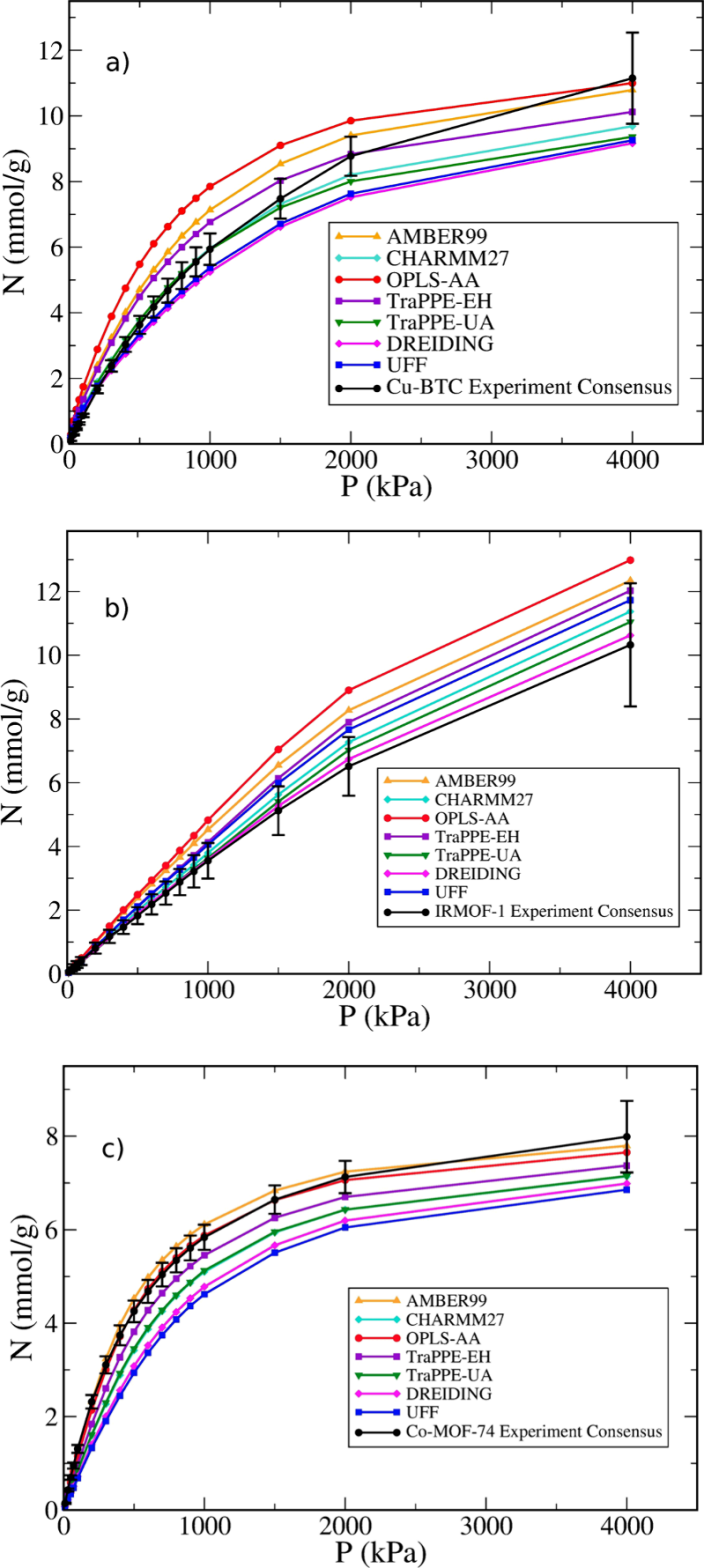
Simulated isotherms for methane at 298 K on (a) Cu-BTC, (b) IRMOF-1, and (c) Co-MOF-74 using different force fields compared to the experimental consensus isotherms (full black lines).

Although Figure 6 shows that, *on average*, simulation predictions are consistent with experimental data, this does not imply the same conclusions are true for individual force fields. Figure 7 compares the individual simulated isotherms against the consensus isotherm for the above-mentioned three MOFs. From this plot, finding a single force field that yields the best agreement with experimental data is much harder. For example, because the Co-MOF-74 simulation consensus lies somewhat below the experimental consensus, the best-performing force fields are those that predict the highest uptake, i.e., OPLS-AA and AMBER-99. Conversely, the simulation consensus lies above the experimental one for IRMOF-1, and therefore, the best-performing models are on the low end of the scale, i.e., DREIDING and TraPPE-UA. However, one might argue that TraPPE-EH provides the best balance over all three of the MOFs because its predictions lie within experimental uncertainty for most of the pressure points considered here.

**Figure 8:**
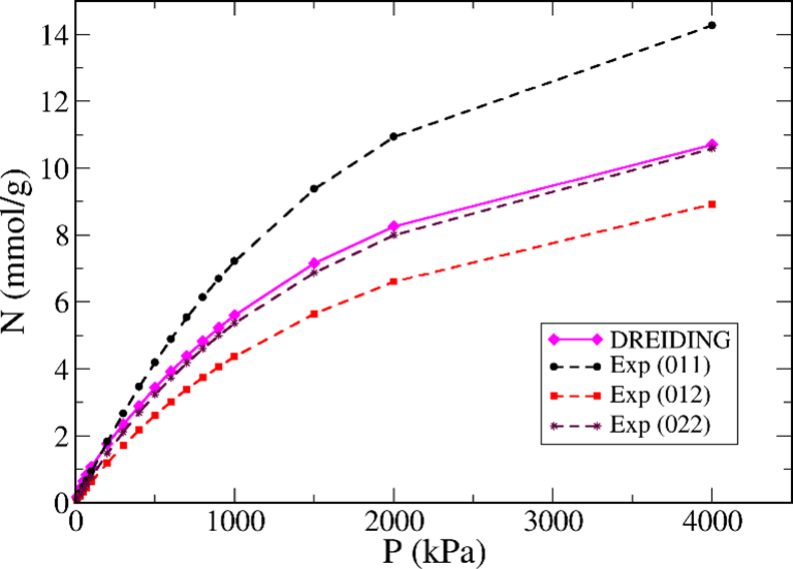
Comparison of predicted adsorption uptake of methane on Cu-BTC at 298 K for the DREIDING force field (magenta line), with experimental isotherms 011 (black circles and dashed line), 012 (red squares and dashed line), and 022 (purple stars and dashed line).

Another interesting comparison is shown in Figure 8. There, we plot the methane adsorption isotherm on Cu-BTC predicted with the DREIDING model, which is probably the most widely used model in simulation studies of adsorption in MOFs, against three selected experimental data sets (not classified as outliers). Although the simulations are in near-perfect agreement with data obtained on sample 022, they significantly underestimate the data for sample 011 and overestimate the data for sample 012. These systematic discrepancies remain, even if we assign a standard uncertainty of ±5% to the experimental data, which is commonly thought to represent an upper limit to the uncertainty of individual measurements. This comparison emphasizes the limitations of comparing simulation results obtained from a single off-the-shelf model against a single experimental adsorption isotherm, which is the standard practice in the field. The interpretation can be remarkably different depending on which experimental isotherm is chosen. For instance, a comparison performed with samples 011 or 012 alone might suggest that the model needs adjustments to match experimental data. Such practices often lead to unpredictable results, as discussed in the introduction (Section 1).

### 3.4 *Ad Hoc* Parameter Adjustments

As seen in Figure 6d, the simulation consensus isotherm greatly overpredicts the experimental methane uptake in MIL-47. Even when comparing the experimental consensus to individual models (Figure S23d), all force fields overpredict methane adsorption by a significant margin beyond the upper limit of the experimental error bars. Furthermore, all force fields predict an isotherm with somewhat different curvature from the experimental one—the uptake is much more pronounced at low pressures, indicating stronger adsorbate-adsorbent interactions than observed experimentally. It is also possible that the experimental isotherm will cross the simulation consensus at higher pressures and yield a higher saturation capacity, but because of the lack of experimental data above 4 bar, this cannot be confirmed at present.

**Figure 9:**
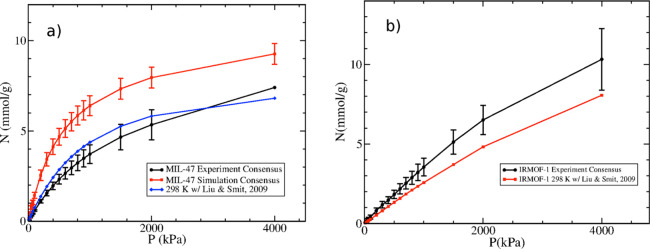
(a) Adsorption isotherms for methane on MIL-47 at 298 K: experimental consensus isotherm with error bars (black circles), simulation consensus isotherm with error bars (red squares), and simulated isotherm using modified parameters from Liu and Smit^20^ (blue diamonds). (b) Adsorption isotherms for methane on IRMOF-1 at 298 K: experimental consensus isotherm with error bars (black circles) and simulated isotherm using modified parameters from Liu and Smit^20^ (red squares).

One commonly used approach to account for this type of discrepancy between simulations and experiments is to assume that the model requires improvement and manually adjust some of the interaction parameters. In fact, Liu and Smit^20^ modified the UFF parameters for the organic linker in the MIL-47 repeating unit to achieve good agreement with an experimental isotherm from Rosenbach et al.^73^ This isotherm was categorized as “red” in our analysis but is actually consistent with our experimental consensus isotherm (see SI). The simulation protocol of Liu and Smit differs from ours in that they employed a cutoff radius of 12.8 Å and shifted potentials with no tail corrections (see Figure S25). We simulated methane on MIL-47 using the same parameters and protocol as Liu and Smit to compare it to the consensus isotherms (see Figure 9a). This modified UFF model indeed provides better quantitative agreement with the experimental consensus isotherm, although it is evident that the curvatures of the two isotherms are still quite different.

The problem with *ad hoc* adjustments in force field parameters to match experimental data on specific systems (in this case, methane on MIL-47) is that they are often not transferable. To examine whether this particular model was transferable, we simulated methane on IRMOF-1 because this MOF has the same organic linker as MIL-47. Figure 9b shows that the modified model of Liu and Smit provides much poorer agreement with the IRMOF-1 experimental consensus isotherm than any of the generic force fields considered in this work. This was expected because the modified parameters were designed to yield weaker interaction energies with the MIL-47 structure than the original UFF force field. Carrying that effect over to IRMOF-1 leads to a systematic underestimation of adsorption over the entire pressure range. This shows that the Liu and Smit model is not transferable and again highlights the pitfalls of tuning parameters to fit a single experimental isotherm.

Apart from force field limitations, there are several possible reasons for the observed discrepancy. Framework flexibility has been observed in other members of the MIL family of MOFs (e.g., MIL-53)^74^ and is well-known to affect adsorption results.^75^ However, no such “breathing” effects are present in MIL-47(V) due to the oxidation state of the metal atom.^76^ Furthermore, the structure used here for MIL-47 would already be in a “large pore” form, so any breathing effects would cause a narrowing of the pore space and lead to an increase of the adsorbate–adsorbent interaction strength at low pressure, the opposite of what is required to improve agreement between simulation and experiment. Other forms of dynamic behavior, such as ligand rotation, are also unlikely to be significant for the MIL-47 linker at room temperature.^77,78^

Defects in the MOF framework can also cause a discrepancy between simulations and experiments because the former are carried out under the assumption that the crystal is perfect. Our pore volume scaling procedure (see Section 2.1) accounts, in an approximate way, for defects that simply induce a relatively small decrease in the adsorption capacity (e.g., the presence of nonadsorbing impurities), but it cannot account for more significant defects, such as missing linkers or missing clusters. Although there is no unequivocal evidence showing that such defects are prevalent in MIL-47 (unlike the case of UiO-66, as discussed in Section 3.5), a more detailed analysis, perhaps using a recently developed framework to generate defective framework models,^79^ is needed to definitively rule out this possibility.

Finally, the origin of the discrepancy may lie in the experimental data itself. It is important to note that MIL-47 was the material for which it was hardest to find valid adsorption isotherms. Ultimately, only three isotherms in total were considered, measured by only two independent research groups, and only one of which was categorized as “green” (i.e., it also reported nitrogen adsorption at 77 K on the same sample). As such, the degree of confidence in the experimental consensus isotherm for this system is comparatively low, and it is likely that the experimental variability for MIL-47 may be underestimated. Further measurements of methane adsorption on this material by independent authors would be quite valuable.

### 3.5 Accounting for Framework Defects

Systematic discrepancies between simulation and experimental consensus isotherms were also observed for UiO-66, with the simulation overpredicting methane uptake in the low to intermediate pressure regions and underpredicting experimental adsorption at high pressures (Figure 6e). Interestingly, the vast majority of experimental samples for this MOF (29 out of 34, i.e., >85%) had pore volumes greater than the theoretical pore volume and were, therefore, not scaled by the pore volume ratio (see Eq 1 in the SI).

The most likely explanation for the above observations is UiO-66’s susceptibility to defects in its structure. This has been amply demonstrated experimentally,^70,80−82^ and the implications have been assessed by several simulation studies.^54,83−85^ The presence of defects in experimental samples would also explain the large number of reported pore volumes that were greater than the theoretical value because the absence of a linker or node would result in more available space in a synthesized sample with an imperfect structure than in the simulated “perfect crystal” structure. To investigate this further, we carried out GCMC simulations on pristine and defective UiO-66 structures kindly provided by Van Speybroeck and co-workers; see the Supporting Information for details on the parameter assignment for these structures. The pristine structure from Van Speybroeck et al. corresponds to a fully hydroxylated structure, but the predicted methane adsorption isotherm is very similar to the one obtained on the dehydroxylated structure from the RASPA database (Figure S26). Furthermore, in agreement with Vandenbrande et al.,^54^ our simulations show that the effect of missing linker defects on methane adsorption isotherms is relatively minor. On the contrary, a single missing cluster defect (i.e., 1:4 concentration of defects) leads to a pronounced difference in the adsorbed amount and in the isotherm curvature (Figure S26). Therefore, we conducted simulations on the missing cluster structure with all force fields considered previously and computed the corresponding consensus isotherm for comparison with experiment.

**Figure 10:**
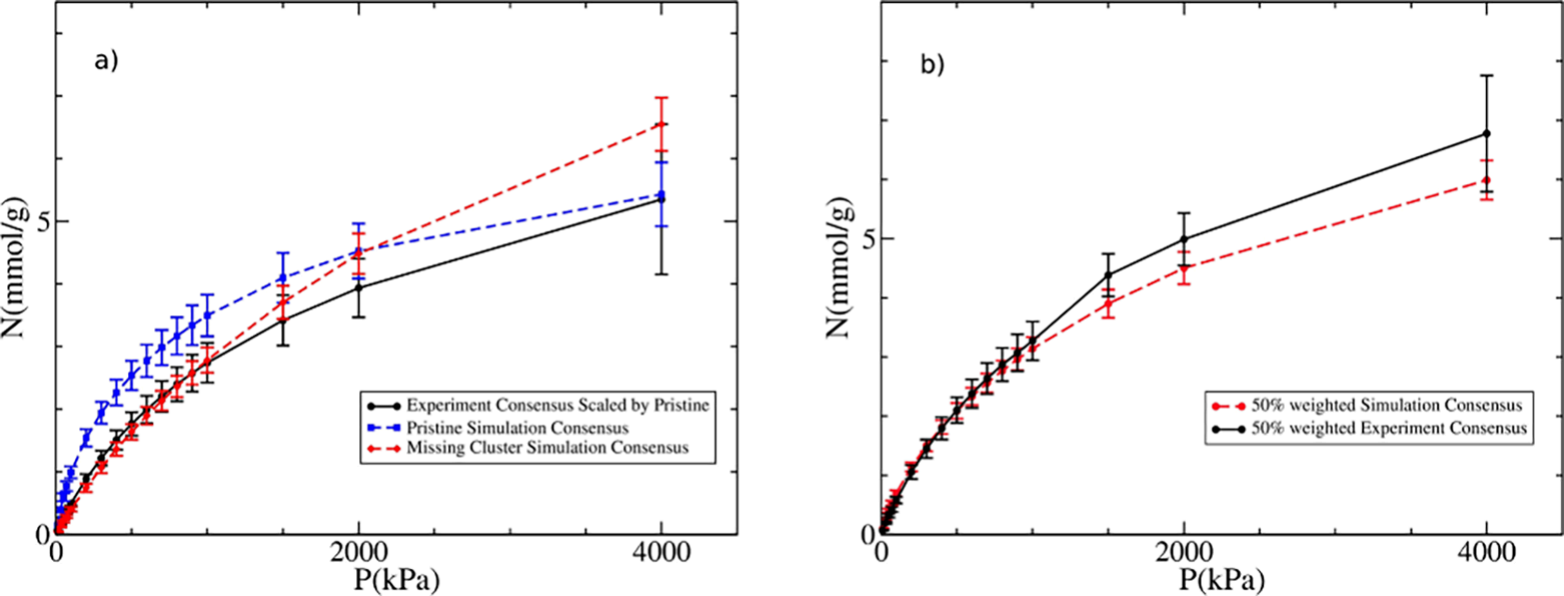
Adsorption isotherms for methane on UiO-66 at 298 K: (a) experimental consensus isotherm with error bars when pore volume scaling by the theoretical pore volume of pristine UiO-66 (black circles), simulation consensus isotherm with error bars for pristine UiO-66 (blue squares and dashed line), and simulation consensus isotherm with error bars for missing cluster UiO-66 (red diamonds and dashed line) and (b) consensus isotherms for both simulated isotherms (red dashed line) and experimental data (full black line) assuming a sample corresponding to 50% pristine and 50% missing cluster structures.

Figure 10a shows that the experimental consensus isotherm agrees better with the missing cluster simulation consensus isotherm at lower pressures but tends to agree more with the pristine simulation consensus isotherm for *P* ≥ 1000 kPa. This suggests that the average defect concentration in the experimental UiO-66 samples should lie somewhere in between those two extremes. To represent this intermediate degree of defects in UiO-66, we produced new simulated and experimental consensus isotherms considering a 50/50 weighting for pristine and missing cluster structures (i.e., assuming that the defect concentration is half that of the original missing cluster structure, or 1:8). For the experimental data, we determined the weighted average of the theoretical pore volumes of both the pristine and the missing cluster structures and used that value for the pore volume scaling of the experimental data (see spreadsheet for “defective UiO-66” in additional information). Encouragingly, the resulting theoretical pore volume (0.69 cm^3^/g) was slightly higher than the largest experimental pore volume of any of the valid samples (0.683 cm^3^/g), meaning that all the experimental UiO-66 isotherms could now be scaled by applying Eq 1 in the SI. The results of this analysis are presented in Figure 10b, which shows remarkably good agreement between simulation and experimental consensus isotherms on a structure estimated to include ∼12.5% missing clusters. This defect concentration is within the order of magnitude of experimental estimates.^80^

## 4 Conclusions

In this work, we have systematically quantified the variation in adsorption simulation predictions that arise from the choice of framework force field. The associated uncertainty was found to be quite large, averaging at about ±12, ± 8, ± 11, ± 10, and ±10% of the amount adsorbed for Cu-BTC, IRMOF-1, Co-MOF-74, MIL-47, and UiO-66, respectively, although the curvature of the simulated isotherms was always very similar among different force fields. This suggests that the variation is mainly due to different interaction strengths between methane and the MOFs rather than to fundamentally different adsorption mechanisms predicted by each model. On average, CHARMM-27 and TraPPE-EH provided simulated isotherms that deviated the least from the simulation consensus, hence leading to the most consistent predictions.

The consensus simulation isotherms were compared to experimental consensus isotherms determined from a large amount of experimental data harvested from the NIST-ISODB and the literature, which were manually curated and analyzed using a combination of the procedures developed by Sholl and co-workers^31^ and Smit and co-workers.^36^ However, in this work, we have opted to scale each experimental isotherm by the ratio of the theoretical and experimental pore volumes, which led to an improved agreement between simulation and experiment. Pore volume scaling is therefore recommended as a relatively simple method to account for slight sample imperfections, most often manifested by experimental pore volumes slightly lower than the theoretical “perfect crystal” values.

The collection and curation of experimental data were fraught with challenges, including occasional errors in the NIST-ISODB database, great variability in the way experimental isotherms are reported (e.g., unit basis), and, more importantly, difficulties in obtaining sample characterization data (e.g., pore volume). As such, considerable time was spent on this part of the analysis to allow us to refine a robust data set that had been thoroughly checked for accuracy. Reporting data such as the specific pore volume and specific surface area of a porous material sample, publishing adsorption data in tabulated form in the Supporting Information, and providing a structure file—such as a Crystallographic Information File (.cif)—for the studied sample are all procedures that would significantly ease the process of data curation and handling. Establishing standard practices will reduce the need for this painstaking digitization of isotherms from figures in academic papers and mitigate the loss of information. In this context, the recent proposal of the “adsorption information file” (AIF) by Evans et al.,^86^ which converts the output files of various file formats from commercial adsorption equipment to a standardized human- and machine-readable file format, is particularly useful. This AIF format has been approved by IUPAC,^87^ and its general adoption by the adsorption community would be most welcome.

Our analysis showed that when comparing consensus experimental and simulation isotherms obtained on “as-received” structures (i.e., corresponding to perfect crystals), good agreement was observed for only three out of five MOFs (i.e., 60%). Interestingly, this includes two materials, Cu-BTC and Co-MOF-74, that possess open metal sites. This confirms previous assertions that interactions between aliphatic hydrocarbons and OMS do not play a major role in adsorption at room temperature and above. The observed discrepancies between simulation and experiment for UiO-66 could be rationally explained by the likely presence of missing cluster defects in experimental samples; in fact, we observed very good agreement for a hypothetical sample containing 1:8 missing clusters. This, once again, emphasizes the importance of careful experimental characterization of samples prior to adsorption measurements and for the detailed reporting of such characterization studies. An even more significant discrepancy was observed for MIL-47, and at present, we do not have a conclusive explanation for this observation. In this context, issues such as insufficient solvent removal or intrinsic framework flexibility, which were not explored in depth here, deserve further consideration.^75^

Overall, this work shows that agreement between simulation and experiment for several off-the-shelf force fields should not be taken for granted, particularly for the most widely used UFF and DREIDING models. TraPPE-EH seems to provide a good balance between consistency and accuracy, with the added benefit of compatibility between adsorbate–adsorbate and adsorbate–adsorbent interactions. However, we emphasize that the present study is restricted to a relatively small number of materials and a single adsorbate (methane); analysis of a much larger number of MOFs is currently hindered by the limited amount of experimental data available and the significant effort required for experimental data collection and curation. We believe that our approach lays the groundwork for more systematic assessments of molecular models in future studies. In this regard, the next step should be to conduct a similar analysis for polar adsorbates, such as carbon dioxide and water, where electrostatic interactions also play a prominent role. It would also be interesting to use this approach to compare the performance and transferability of generic force fields like those analyzed here against fully quantum-mechanically derived models. In this context, extending our approach to zeolites holds promise in light of recent systematic efforts to develop and test predictive force fields for this class of materials.^88,89^

Finally, our work emphasizes the pitfalls of the standard approach of comparing simulations from a single force field against a single experimentally measured isotherm without adequate consideration of the sources of uncertainty in both simulations and experiments. *Ad hoc* adjustments of force field parameters based on such limited comparisons are almost certain to lead to a lack of transferability and potentially erroneous predictions, as demonstrated here in the case of MIL-47. Efforts to parameterize force fields for adsorption in MOFs should instead use a wide range of structures and experimental data that are demonstrably reproducible for both training and validation purposes.

## Data Availability

Spreadsheets containing all the collected and processed experimental data are provided and input files for all adsorption simulations are freely available from the University of Strathclyde KnowledgeBase https://doi.org/10.15129/d9d42c14-72fd-4aa5-a0a2-f005dadf4ccb.

## Supporting Information Available

Additional details and figures for the experimental data collection and curation; examples of pore volume calculations and isotherm fits; additional force field tests and full tables of parameters; and additional details for simulations on UiO-66 defective structures (PDF)
