# Supplementary material for: Quantifying the Uncertainty of Force Field Selection on Adsorption Predictions in MOFs
Source: J Chem Theory Comput. 2024 May 31;20(11):4869–84. doi: 10.1021/acs.jctc.4c00287 (PMC11171284; doi:10.1021/acs.jctc.4c00287)
Supplement: Supplementary file 1 — ct4c00287_si_001.pdf [file ct4c00287_si_001.pdf]

Supporting Information for:

Quantifying the Uncertainty of Force Field Selection on Adsorption Predictions in MOFs

Connaire McCready[1], Kristina Sladekova[1], Stuart Conroy[1], José R. B. Gomes[2], Ashleigh Fletcher[1], Miguel Jorge[1,]*

[1] Department of Chemical and Process Engineering, University of Strathclyde, 75 Montrose Street, Glasgow G1 1XJ, United Kingdom

[2] CICECO – Aveiro Institute of Materials, University of Aveiro, Campus Universitário de Santiago, Aveiro, Portugal

[*] Email: miguel.jorge@strath.ac.uk

1. Detailed procedure for experimental data collection and curation

As explained in the main paper, our study focused on methane isotherms measured at 298 K, but we allowed for a variation of ± 5 K in line with the approach of Park et al.[1] The starting point for our data collection was the NIST/ARPA-E Database of Novel and Emerging Adsorbent Materials (NIST-ISODB).[2] Digitising data from peer-reviewed literature reports, the NIST-ISODB contains both experimental and modelling results. Initially, only experimental data was taken for our analysis by applying the "experiment" filter in the Measurements tab of the search function. However, we later realised that there were several instances where simulated isotherms were mislabelled as experimental; therefore, we eliminated this filter and manually curated each methane isotherm collected from NIST-ISODB by checking against the original literature source. Through this manual curation, we also identified a few instances where the original source of the dataset did not correspond to the DOI reported in the NIST-ISODB. In such cases, we checked the NIST-ISODB for accuracy against the original source of the experimental measurements. We found that most of the data reported in the NIST-ISODB were reliable. However, there were a few cases where isotherms had to be (re-)digitised manually due to either a scarcity of data points in the NIST-ISODB, incorrect units being reported (including a logarithmic pressure axis being incorrectly digitised as linear), or even the complete absence of a pertinent isotherm. Those cases are identified in the Supporting Information datasheets related to this publication, which contain all the

experimental isotherms collected here. A manual literature search subsequently supplemented the data collection on NIST-ISODB – we used Clarivate's Web of Science with keywords "(methane OR CH4) AND (MOF)", where "(MOF)" refers to the material name, including synonyms where relevant. This was required for all MOFs studied here due to a shortage of viable isotherms (see below for details); Cu-BTC was the exception, due to the large number of viable datasets collected from NIST-ISODB. Figure S1 shows the full set of collected experimental isotherms for Cu-BTC over the entire range of pressures.

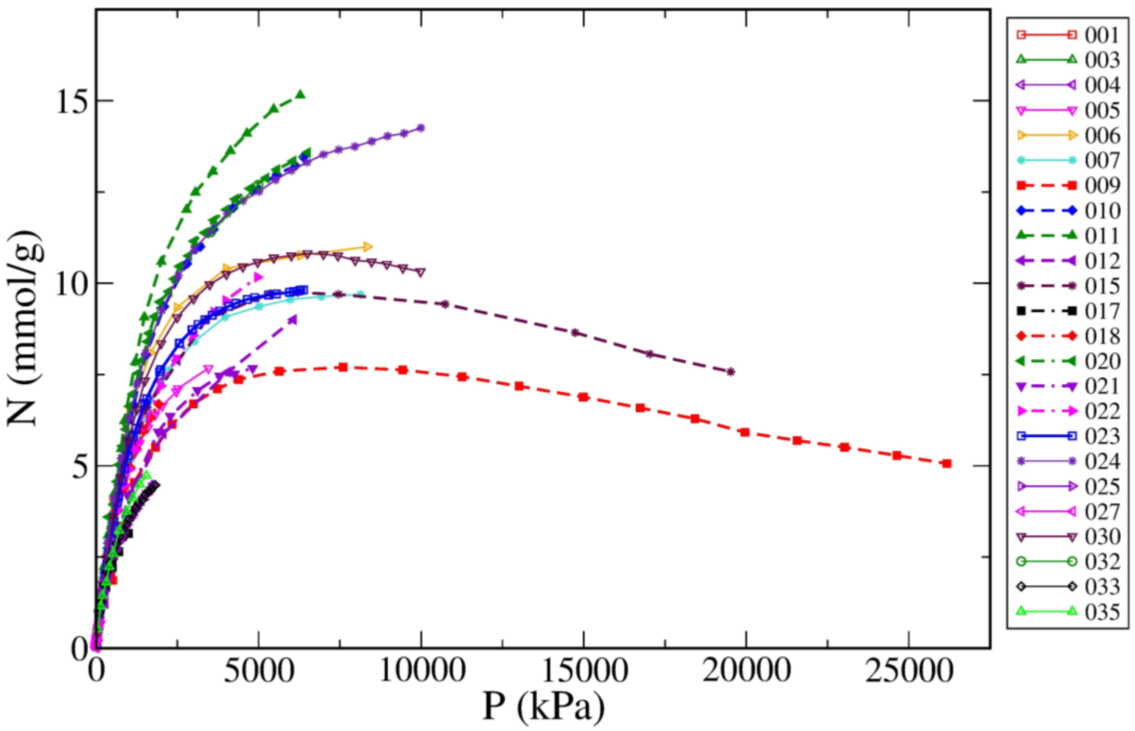

Figure S1. *Isotherms collected from the NIST-ISODB for methane on Cu-BTC at 298 ± 5 K over the entire pressure range. The labels in the legend correspond to individual entries in the data spreadsheets provided as further Supporting Information.*

After collection, isotherms were classified into three categories based on their reporting of pore volume information. Isotherms were labelled: i) green when full N_2 (77 K) or Ar (87 K) isotherms were also reported for the same sample, or they could be retrieved from appropriately cited earlier work; ii) amber when a numerical value for the pore volume of the same sample was reported or retrievable from earlier references, but the original N_2/Ar isotherms were not reported; iii) red when no information on the pore volume or N_2/Ar isotherms was provided for that sample. As mentioned above, the main reason for this classification was due to our decision

to scale the experimental isotherms using the pore volume ratio, which obviously requires knowledge of the experimental pore volume of the sample. Figure S2 shows the full set of raw experimental isotherms for Cu-BTC with the above colour coding. Notice that we limit our analysis to pressures below 6000 kPa – above this pressure, data is much sparser.

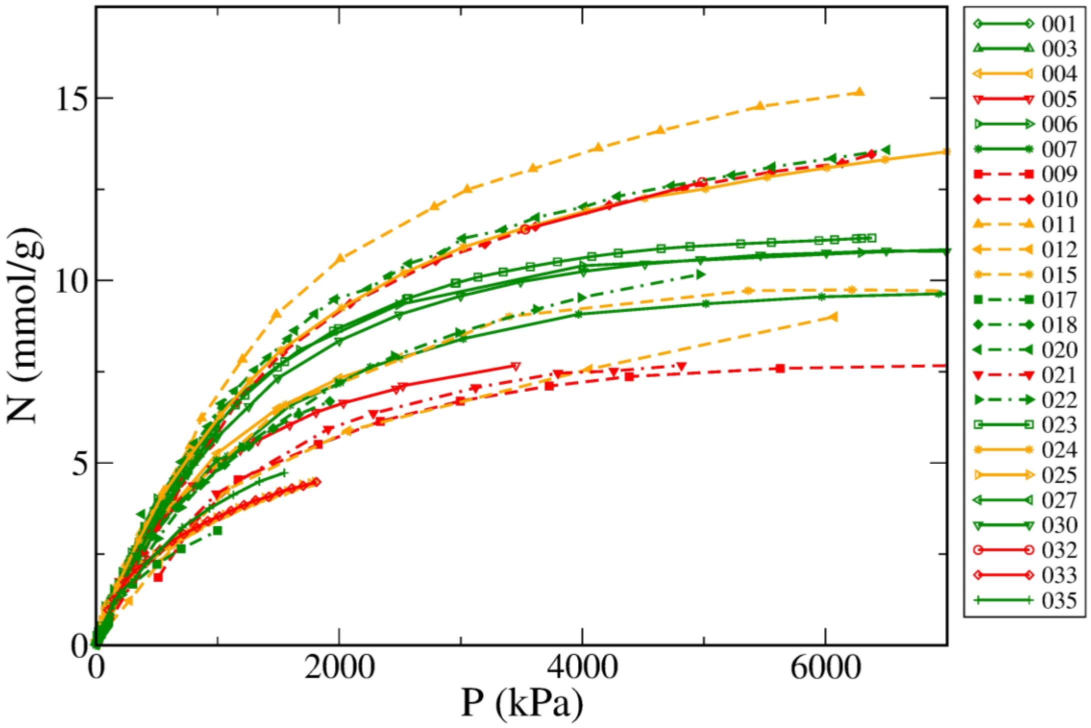

Figure S2. *Isotherms collected from the NIST-ISODB for methane on Cu-BTC at 298 ± 5 K, colour-coded according to the reporting of pore volume characteristics: N_2/Ar isotherms reported – green; only pore volume reported – amber; no pore volume information – red.*

After manual curation was concluded, the isotherms classified as “red” were discarded since they could not be scaled due to the absence of pore volume information. Note, however, that this does not imply that all those isotherms are incorrect or invalid; in fact, some of the red isotherms appeared to be consistent with most green and amber isotherms, as shown in Figure S2. However, without data on the pore volume of the particular sample, it is hard to assess whether this was indeed the case.

This "traffic light" categorisation system allowed us to scale the amber and green isotherms, despite their differing pore volumes, and compare them in a fairer manner than merely comparing the adsorption uptake on samples that could be characteristically dissimilar.[1,3–5] More importantly, this scaling allows us to account, in an approximate way, for impurities

present in the experimental sample and compare them to simulated isotherms that correspond to a perfect crystal. The pore volume scaling takes the form:

$$\begin{cases} n_{scaled} = n_{ads} \frac{v_p^{computed}}{v_p^{experimental}} & for \quad v_p^{computed} > v_p^{experimental} \\ n_{scaled} = n_{ads} & for \quad v_p^{computed} \leq v_p^{experimental} \end{cases} \tag{1}$$

Where n_{ads} is the original experimental adsorption uptake, $v_p^{computed}$ is the computed theoretical pore volume, and $v_p^{experimental}$ is the experimental pore volume of the sample. For amber isotherms, the latter corresponded to the value reported in the corresponding publication, while for green isotherms, it was recalculated manually from the N_2 (77 K)/Ar (87 K) isotherms provided, even when a numerical pore volume value was also reported. In such cases, the Gurvitsch rule[6] was applied:

$$v_{p,G} = \frac{n_{ads}^{Sat}}{\rho_{liq}} \tag{2}$$

where $v_{p,G}$, n_{ads}^{Sat}, and ρ_{liq} are the total specific pore volume, adsorption uptake at saturation, and density of liquid adsorbate, respectively.[7] This approach assumes that the fluid adsorbed in the pores has the same density as the bulk liquid at the same temperature and pressure. The Gurvitsch rule has been shown to agree with geometric pore volume calculations on many microporous materials, despite the approximations involved.[8]

The adsorbed amount of N_2/Ar at saturation was estimated by carrying out a linear least-squares fit of the plateau region of the adsorption isotherm and interpolating or extrapolating to $P/P_{Sat} = 0.99$. In this section, we provide an example of such a calculation for Cu-BTC sample 001.[9] Spreadsheets for all other samples are provided as a dataset adjoining the paper.

The first step is to identify the plateau region of the adsorption isotherm. This is beyond the initial region of a steep increase due to strong adsorbate-adsorbent interactions but excludes data after any inflection point observed at very high pressure. Such inflection points are normally due to the condensation of liquid in macropores and/or in the sample container. Figure S3 shows a typical example where the initial steep increase region (for this data set, below $P/P_{Sat} \sim 0.3$) and the condensation region (above $P/P_{Sat} \sim 0.8$) were eliminated from the fitting process. We can see that the plateau region is very well described by a straight line, which

allows us to estimate the adsorbed amount at P/P_{Sat} = 0.99 (last point of the red line in Figure S3). This procedure ensures that the experimental pore volume is calculated consistently for all data sets, regardless of the quality of the underlying isotherm data, thus enabling a direct comparison between different samples.

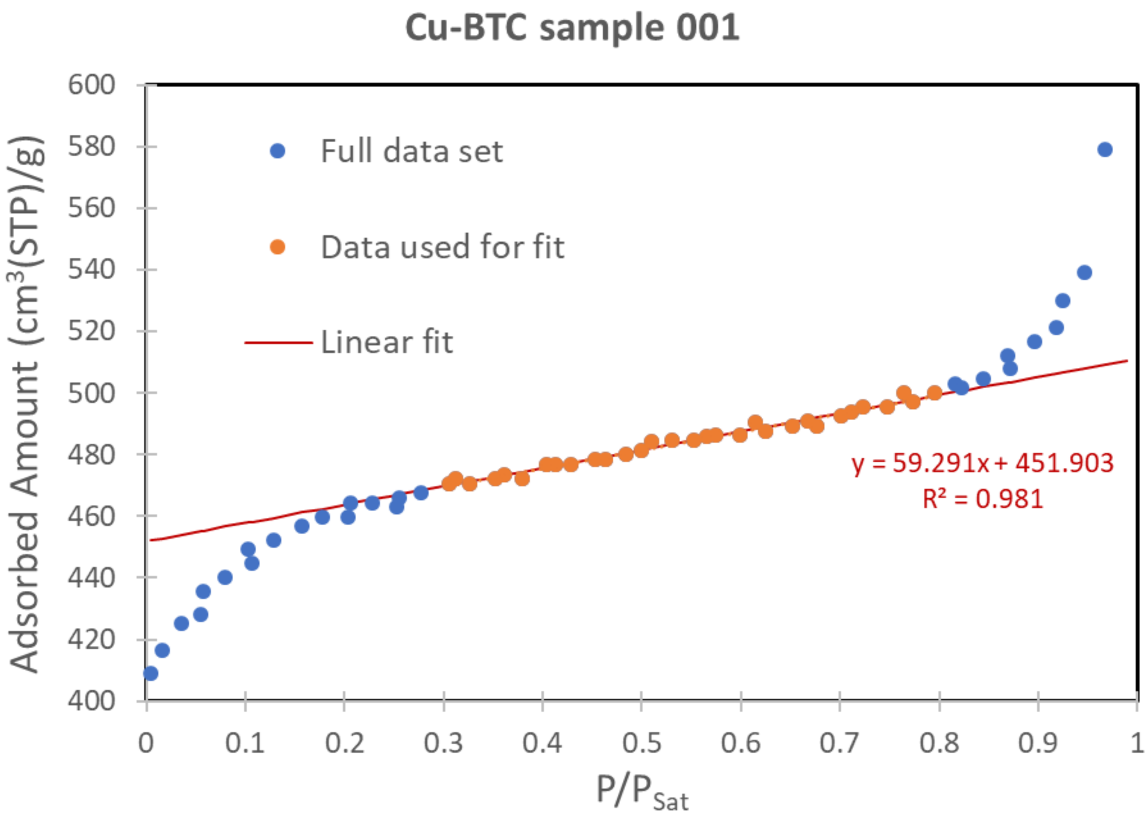

Figure S3. *N_2 adsorption isotherm at 77 K for Cu-BTC sample 001 (blue circles). The subset of data used to perform the linear least-squares fit is shown as orange circles, while the line of best fit, extrapolated to P/P_{Sat} = 0.99 is shown as a red line. The label shows the straight-line equation and corresponding correlation coefficient.*

For this particular sample, n_{ads}^{Sat} was estimated as 510.6 cm^3(STP)/g, which was then divided by ρ_{liq} (i.e. 0.8077 g/cm^3 for N_2 at 77 K and 1 bar) and converted to the required units according to Equation (3):

$$v_{p,G} = \frac{n_{ads}^{Sat} \times M_{N2}}{\rho_{liq} \times 0.022414} \quad \textbf{\textit{(3)}}$$

where M_{N2} is the molar mass of nitrogen (g/mol) and 0.022414 is the conversion factor in cm^3(STP)/mol. This yields a pore volume of 0.790 cm^3/g for this sample, which is not far below the theoretical pore volume of 0.813 cm^3/g calculated from a simulation of N_2 adsorption at 77 K and P/P_{Sat} = 0.99 on a pure crystal unit cell of Cu-BTC. $v_p^{computed}$ was also calculated

from the Gurvitsch rule, where the adsorbed amount was obtained from a GCMC simulation of N_2 at 77 K and P/P_{Sat} = 0.99. In this case, no linear interpolation was needed since we could precisely specify the equilibrium pressure. Although it is possible to estimate the theoretical pore volume from geometric methods,[10–13] we opted for the Gurvitsch rule to ensure complete consistency between the values of $v_p^{computed}$ and $v_p^{experimental}$ used in Equation (1).

It is important to note that the formulation of Equation (1) implies that pore volume scaling is only applied when the theoretical pore volume exceeds the experimental pore volume. This makes physical sense since the procedure is designed to approximately correct for the presence of non-adsorbing impurities in realistic MOF samples – in other words, it assumes that the real sample can be approximated as a mixture of pure MOF crystal and a non-adsorbing component that only contributes to the sample mass but not to the adsorbed amount. Under this assumption, the theoretical pore volume should always be higher than the experimental one. This was indeed observed for the vast majority of isotherms collected on Cu-BTC, IRMOF-1, Co-MOF-74 and MIL-47. For UiO-66, however, most samples had pore volumes that exceeded the theoretical estimate, sometimes by as much as 50%. This clearly suggests that UiO-66 samples are highly defective and do not align with the simple approximation described above (see also discussion in the main paper).

For the example of Cu-BTC, Figure S4 shows the dataset after discarding the "red" categorised isotherms and scaling the remaining isotherms by the pore volume ratio.

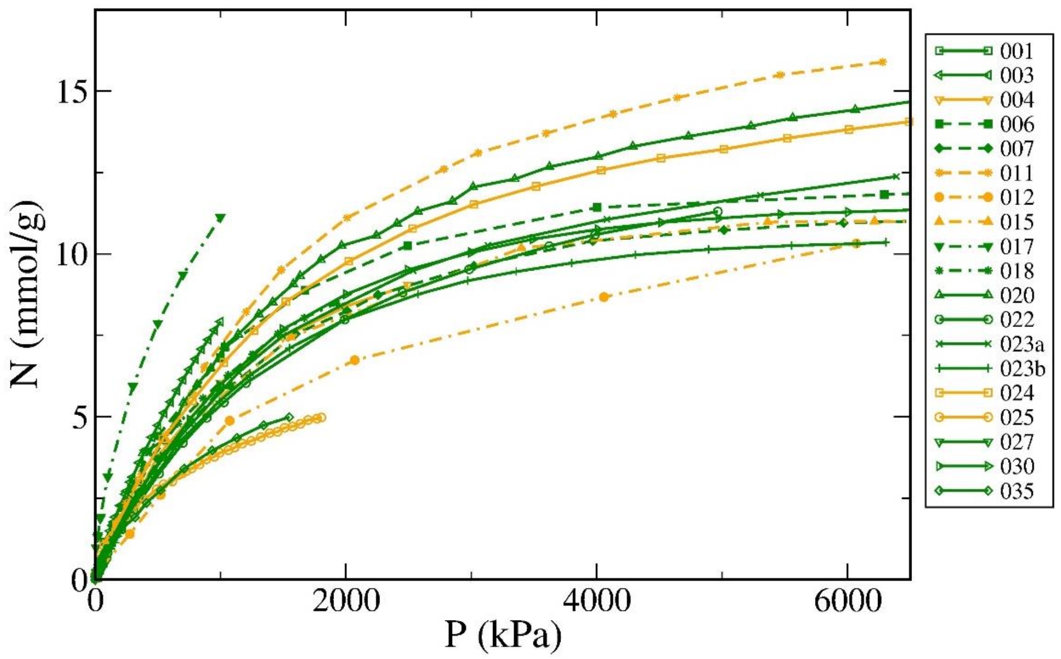

Figure S4. *Isotherms collected from the NIST-ISODB for methane on Cu-BTC at 298 ± 5 K. Data with no pore volume information was discarded and remaining isotherms (green and amber) were scaled by the ratio of the theoretical to the experimental pore volume.*

The scaled adsorption uptake values for each isotherm were then fitted to the Toth isotherm model,[14] given by:

$$n = \frac{n_m p}{\left(1/K_t + p^t\right)^{1/t}} \tag{4}$$

where p is pressure, K_t and t are Toth isotherm constants, and n_m is the monolayer adsorption capacity (the latter three variables are the fitting parameters). The Toth isotherm model was chosen because it captures the curvature of all isotherms well with a limited number of fitting parameters (3). Figure S5 shows a typical example of fitting the Toth model to experimental methane adsorption data. This was carried out after pore volume scaling. It was observed that in some samples the excess methane adsorption isotherm started to curve downwards at high pressures, typically above ~6500 kPa. Since the Toth model is not designed to take this into account (i.e. it assumes absolute adsorption), we opted not to include data above 6500 kPa in our fitting or subsequent analysis.

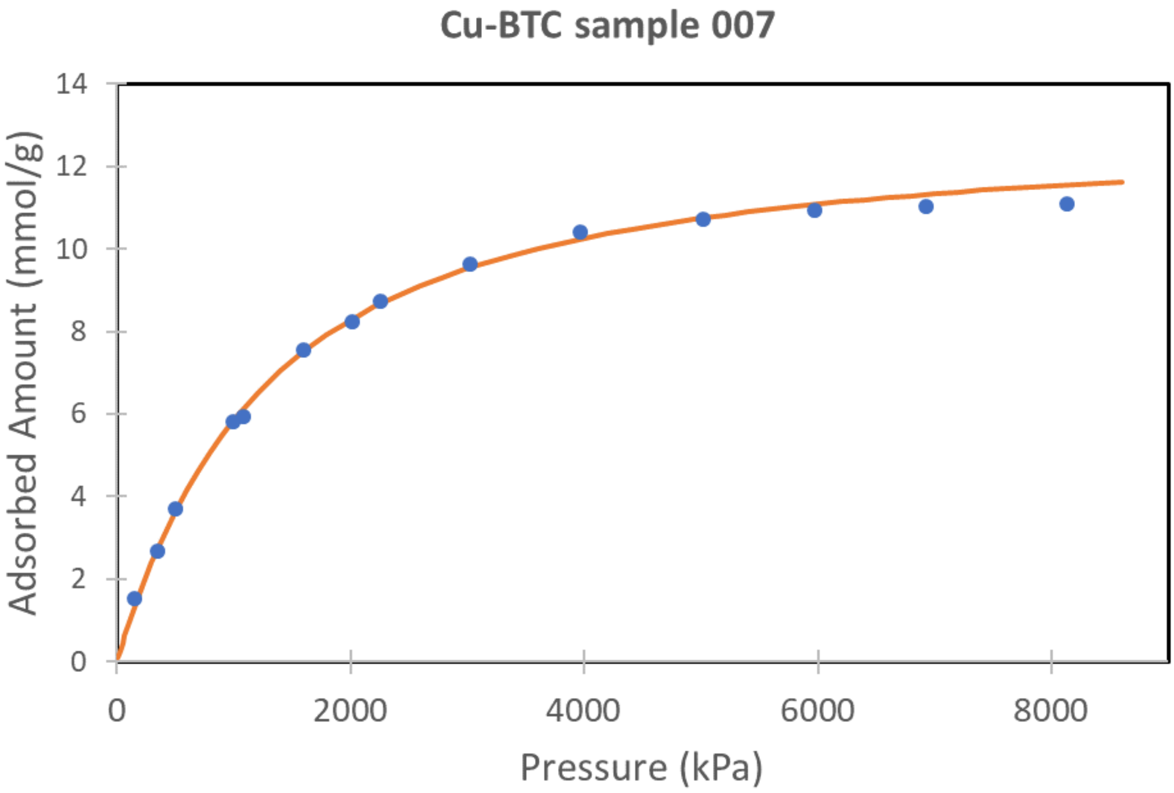

Figure S5. *Methane adsorption isotherm at 298 K on Cu-BTC sample 007 (blue circles). The Toth isotherm fit is shown as an orange line. The last two data points at high pressure were not included in the fit (see text for details).*

It can be seen that the Toth model provides a very good fit over the entire pressure range considered here, allowing for accurate interpolation of the adsorbed amount. Notice that, as well as allowing for statistical analysis to be carried out over multiple experimental isotherms, this fitting procedure also helps to smooth the data and compensate for errors arising during the manual digitization of isotherms.

Using the fitted model parameters, we re-plotted each isotherm using a fixed set of pressure values, namely [10, 30, 50, 70, 100, 200, 300, 400, 500, 600, 700, 800, 900, 1000, 1500, 2000, 4000, 6000] kPa, to allow direct comparison and statistical analysis between measurements at the same pressure. Note that, from the above range, only pressure points up to the maximum pressure reported experimentally for a given isotherm were considered in the analysis (see Figure S6 – i.e. the Toth model was not used to extrapolate isotherms beyond their maximum reported pressure.

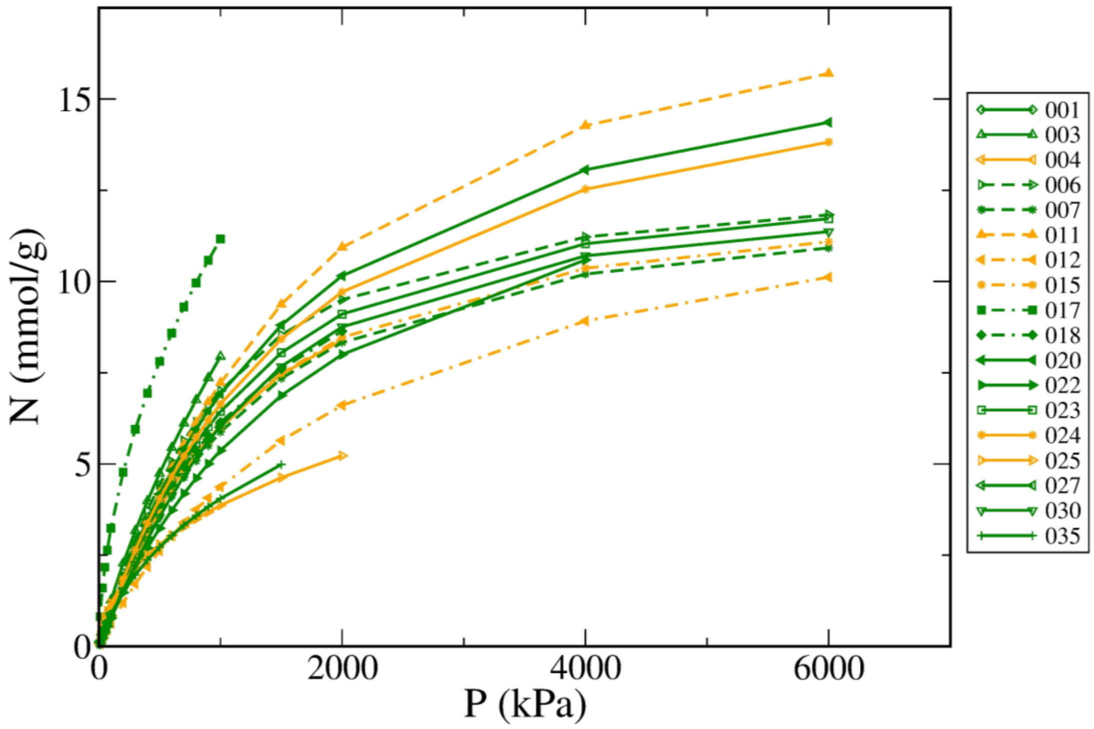

Figure S6. *Isotherms collected from the NIST-ISODB for methane on Cu-BTC at 298 ± 5 K after scaling by the pore volume ratio, fitting to the Toth isotherm model and replotting over the same pressure range. The colour code is the same as in Figure 1, but isotherms classified as "red" were discarded from further analysis.*

Outliers were identified by applying Tukey's method[15] to the data for each pressure point. Any isotherm with more than one outlier pressure point was discarded from the calculation of the average consensus isotherm. In total, 3 isotherms were marked as outliers for Cu-BTC, corresponding to ~16% of the green/amber subset; this is in broad agreement with the estimate of ~20% observed by Park et al. for CO_2 adsorption.[1] The remaining isotherms were used to calculate an average of the experimental data and the 95% confidence interval error bars for each pressure point, as shown in Figure S7.

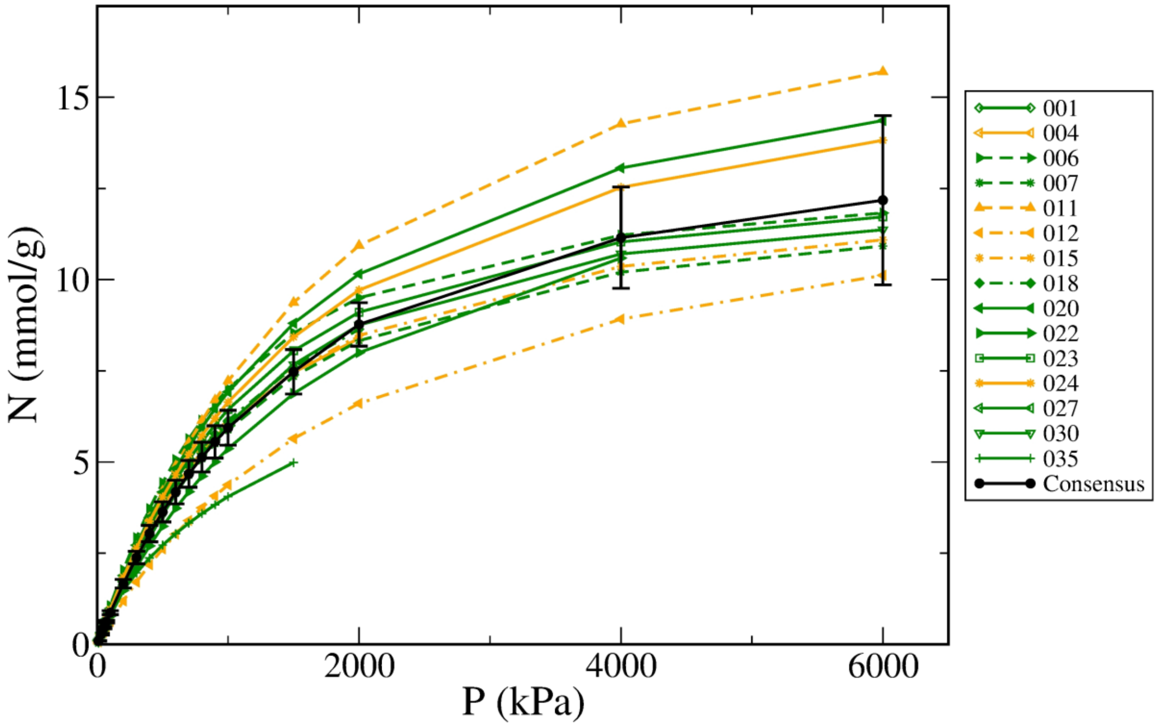

Figure S7. *Isotherms collected from the NIST-ISODB for methane on Cu-BTC at 298 ± 5 K after scaling by the pore volume ratio, fitting to the Toth isotherm model and removing outliers. Also shown is the consensus isotherm with error bars (black line).*

For each of the remaining MOFs, we first show a plot with the complete set of collected experimental isotherms over the entire pressure range, where each isotherm is labelled according to the adjoining spreadsheet (see, e.g. Figure S8 for IRMOF-1). We then show a set of four plots describing the curation process: a) raw isotherms over a restricted pressure range up to 6000 kPa and colour-coded according to the pore volume information provided; b) isotherms scaled by the pore volume ratio, after discarding those classified as “red”; c) isotherms replotted over a fixed set of pressures after fitting to the Toth isotherm model; d) the final set of rescaled and fitted isotherms, with outliers removed by applying Tukey’s method, including the consensus isotherm with 95% confidence interval error bars.

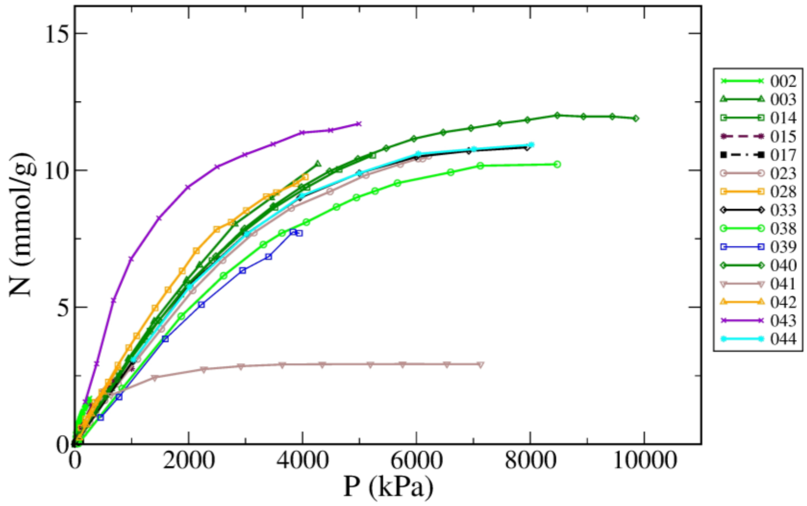

Figure S8. *Isotherms collected for methane on IRMOF-1 at 298 ± 5 K over the entire pressure range. The labels correspond to individual entries in the data spreadsheets provided in SI.*

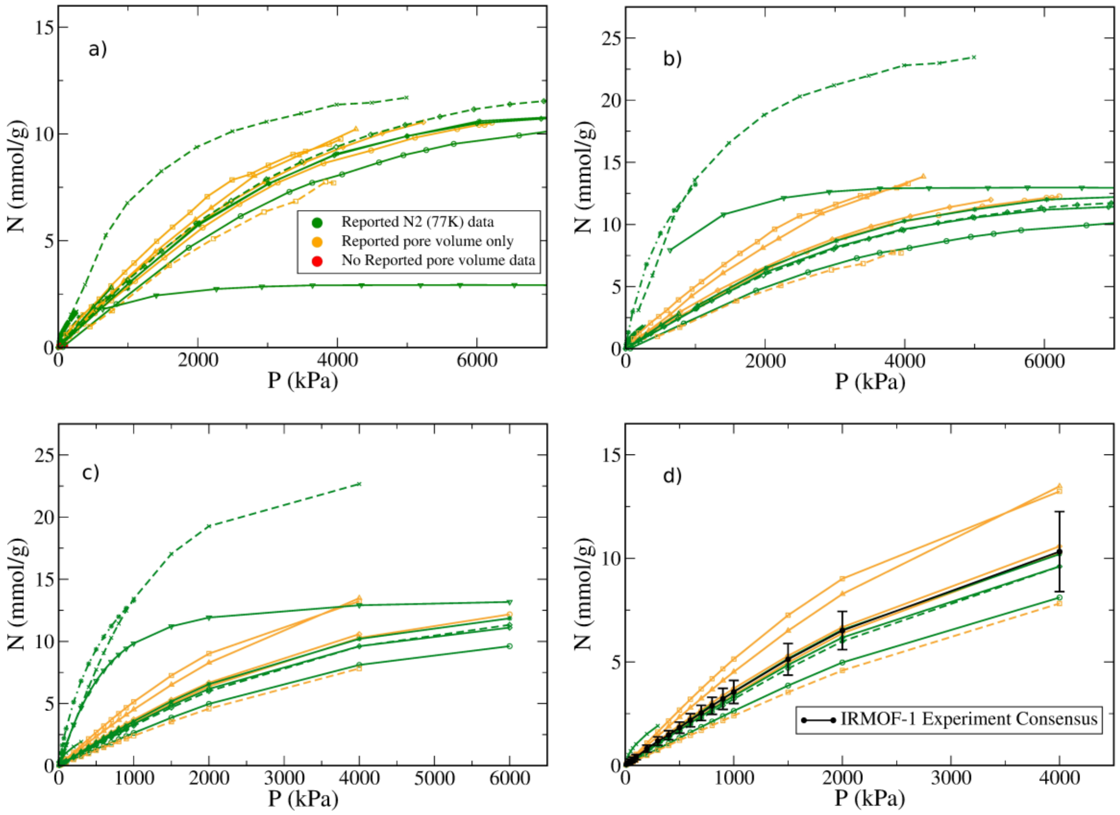

Figure S9. *Excess methane adsorption on IRMOF-1 at 298 ± 5 K. a) raw isotherms over a restricted pressure range up to 6000 kPa and colour-coded according to the pore volume information provided; b) isotherms scaled by the pore volume ratio, after discarding those classified as "red"; c) isotherms replotted over a fixed set of pressures after fitting to the Toth isotherm model; d) the final set of rescaled and fitted isotherms, with outliers removed by applying Tukey's method, including the consensus isotherm with 95% confidence interval error bars.*

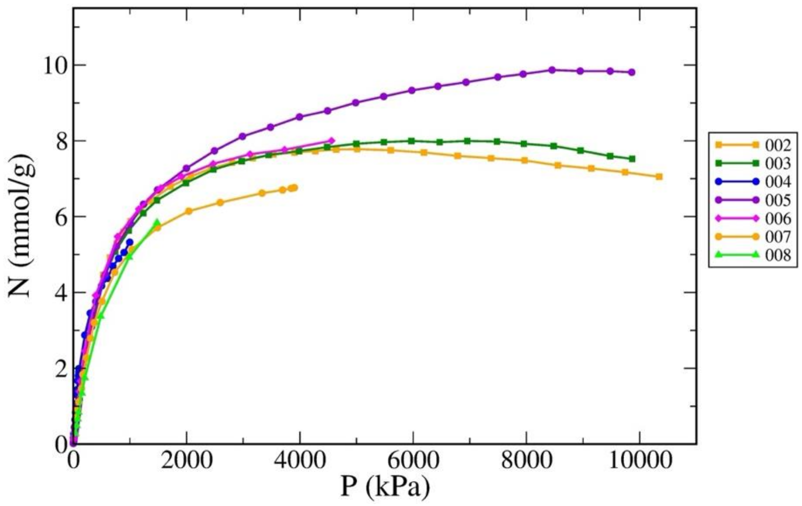

Figure S10. *Isotherms collected for methane on Co-MOF-74 at 298 ± 5 K over the entire pressure range. The labels correspond to individual entries in the data spreadsheets provided in SI.*

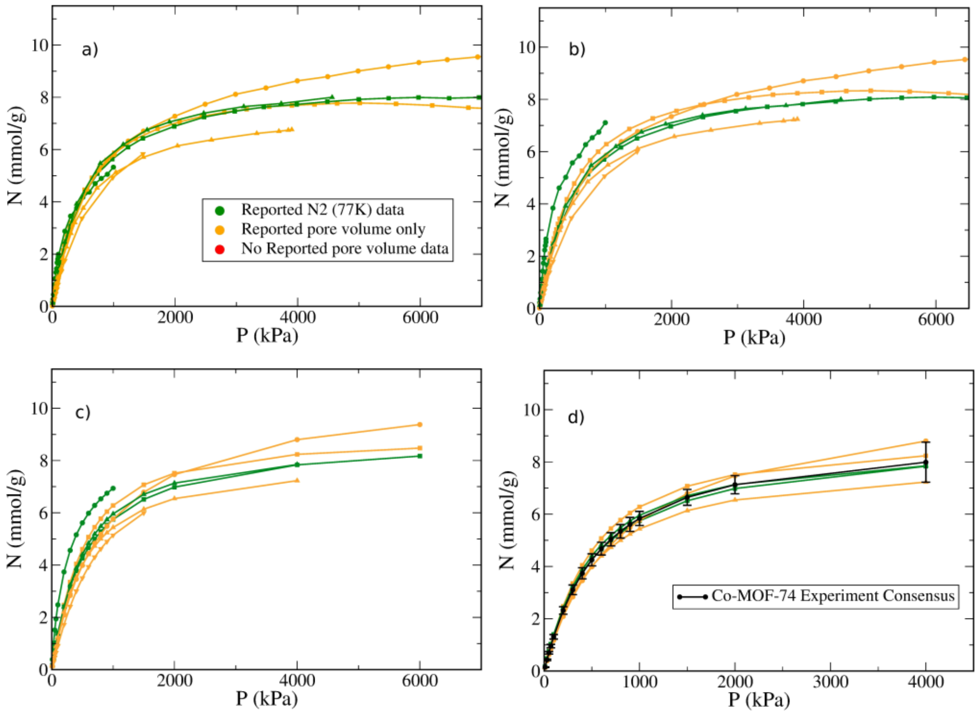

Figure S11. *Excess methane adsorption on Co-MOF-74 at 298 ± 5 K. a) raw isotherms over a restricted pressure range up to 6000 kPa and colour-coded according to the pore volume information provided; b) isotherms scaled by the pore volume ratio, after discarding those classified as "red"; c) isotherms replotted over a fixed set of pressures after fitting to the Toth isotherm model; d) the final set of rescaled and fitted isotherms, with outliers removed by applying Tukey's method, including the consensus isotherm with 95% confidence interval error bars.*

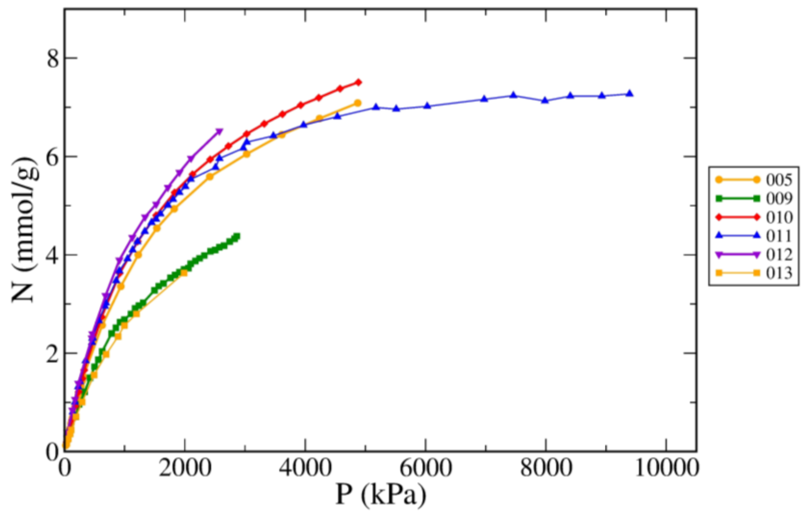

Figure S12. *Isotherms collected for methane on MIL-47 at 298 ± 5 K over the entire pressure range. The labels correspond to individual entries in the data spreadsheets provided in SI.*

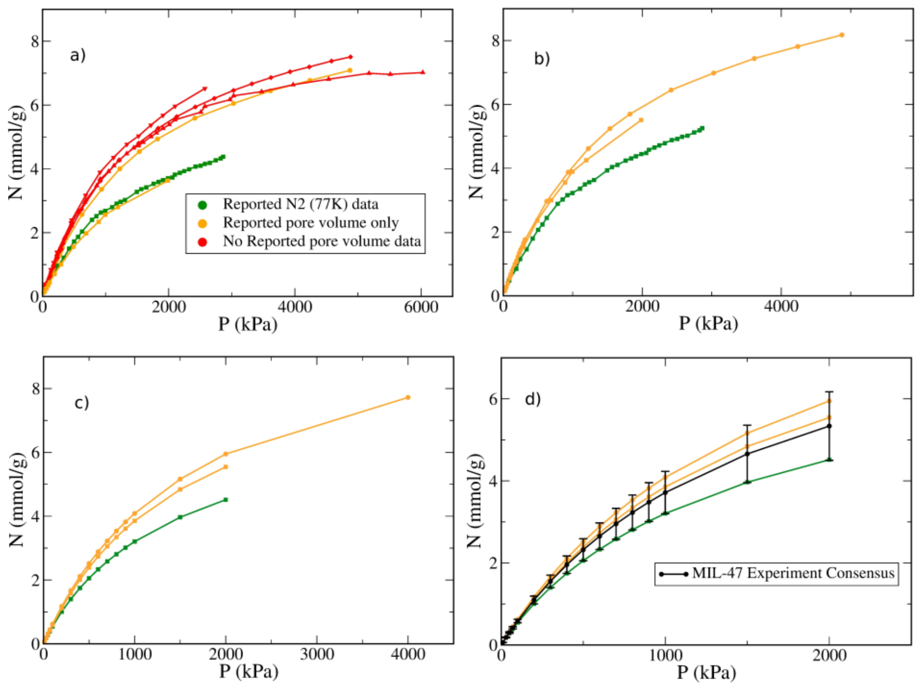

Figure S13. *Excess methane adsorption on MIL-47 at 298 ± 5 K. a) raw isotherms over a restricted pressure range up to 6000 kPa and colour-coded according to the pore volume information provided; b) isotherms scaled by the pore volume ratio, after discarding those classified as "red"; c) isotherms replotted over a fixed set of pressures after fitting to the Toth isotherm model; d) the final set of rescaled and fitted isotherms, with outliers removed by applying Tukey's method, including the consensus isotherm with 95% confidence interval error bars.*

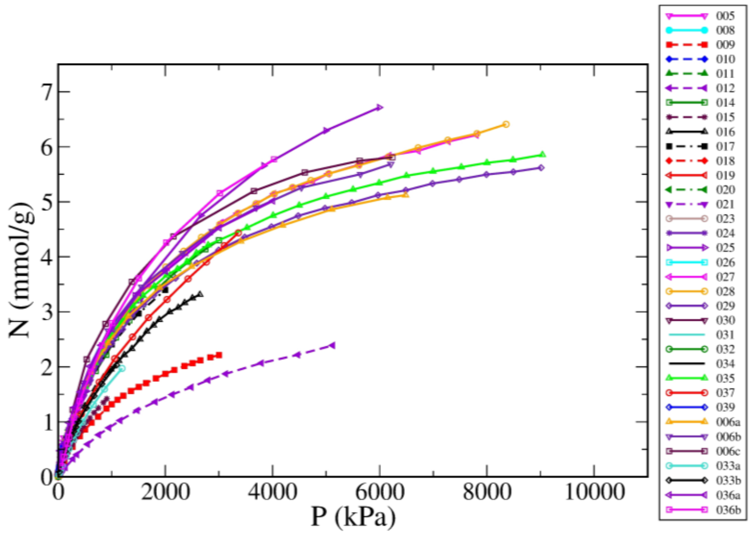

Figure S14. *Isotherms collected for methane on UiO-66 at 298 ± 5 K over the entire pressure range. The labels correspond to individual entries in the data spreadsheets provided in SI.*

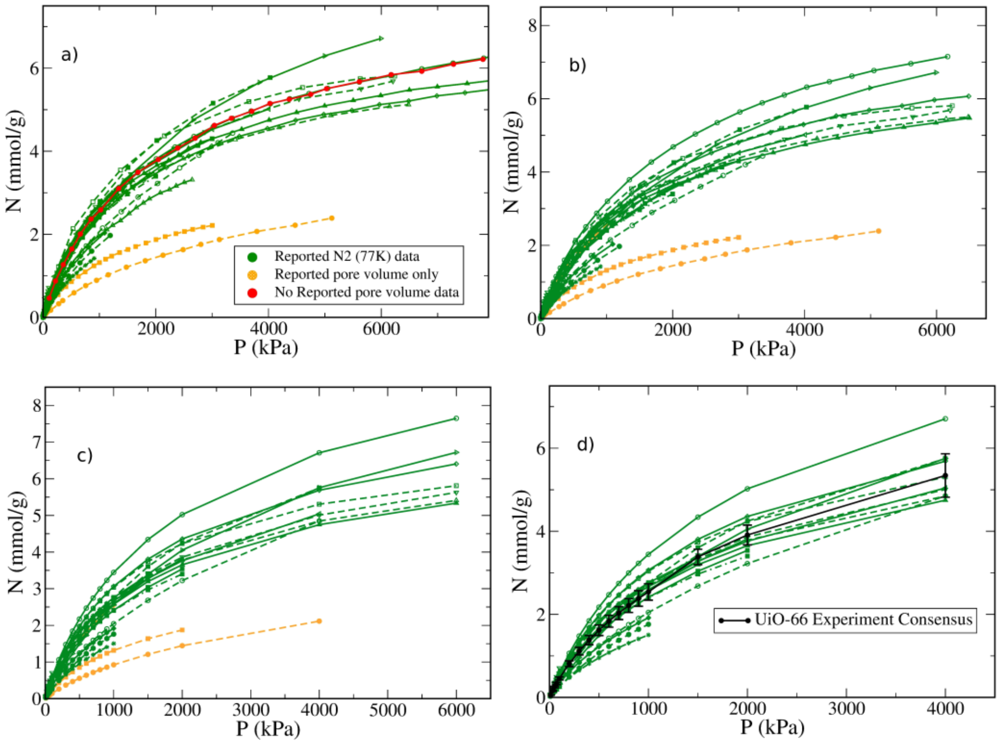

Figure S15. *Excess methane adsorption on UiO-66 at 298 ± 5 K. a) raw isotherms over a restricted pressure range up to 6000 kPa and colour-coded according to the pore volume information provided; b) isotherms scaled by the pore volume ratio, after discarding those classified as "red"; c) isotherms replotted over a fixed set of pressures after fitting to the Toth isotherm model; d) the final set of rescaled and fitted isotherms, with outliers removed by applying Tukey's method, including the consensus isotherm with 95% confidence interval error bars.*

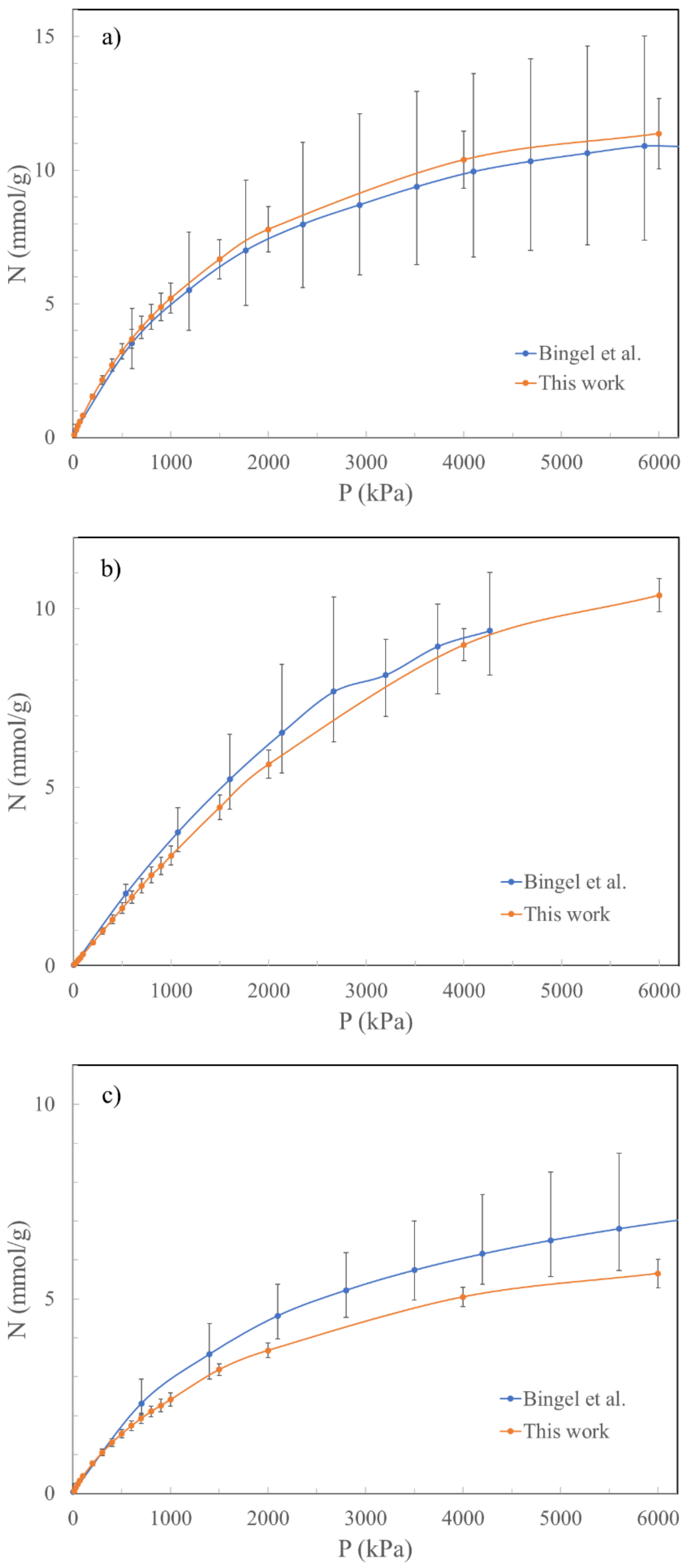

Figure S16. *Comparison of consensus isotherms from this work and from Bingel et al.*[16] *with error bars obtained by their respective methods: (a) Cu-BTC, (b) IRMOF-1, (c) UiO-66.*

2. Effect of cut-off radius and tail corrections

To save computational time, it is important to minimise the unit cell size – RASPA requires the unit cell size to be at least twice the cut-off radius. We resolved to use a cut-off of 11 Å with truncation and a long-range tail correction. Figure S17 shows the comparison between 12.9 Å with shifted potentials and no tail corrections (the RASPA default protocol), 12.9 Å truncated potentials and no tail corrections, 12.9 Å truncated potentials and tail corrections and 11 Å truncated potentials and tail corrections. We can clearly see that applying a shifted potential scheme lowers the amount adsorbed compared to a direct truncation since the entire potential energy curve is shifted upwards. While a shifted potential is important to ensure the absence of spikes in the calculation of forces in Molecular Dynamics simulations, this issue is irrelevant in Monte Carlo simulations as performed in this work. Furthermore, when tail corrections are applied, the adsorption isotherm becomes independent of the choice of cut-off radius. This improves consistency and makes it easier to compare results obtained by different groups. Furthermore, it is a better approximation of the "correct" potential energy arising from the selected force field parameters. Therefore, we recommend this protocol for future simulations of adsorption.

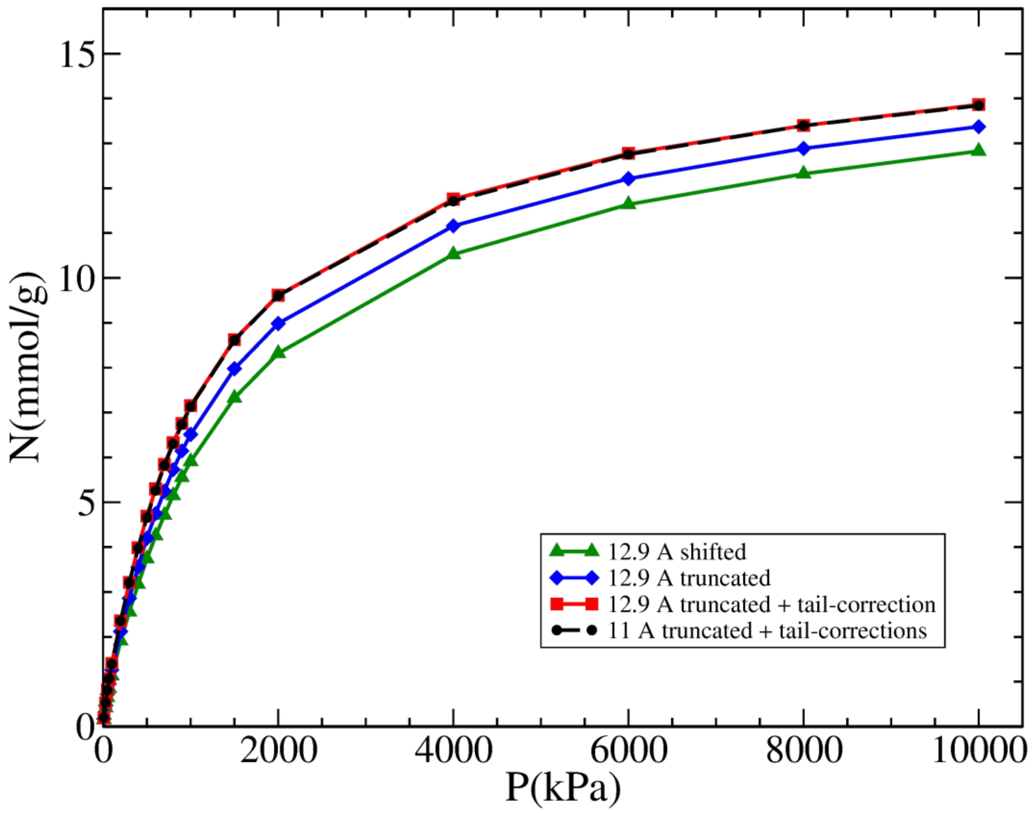

Figure S17. *Effect of shifted and truncated potentials and tail corrections on methane adsorption on Cu-BTC using the TraPPE-EH model.*

3. Additional force field tests

In the AMBER-99 force field, all carbon atoms were assigned the same LJ parameters, corresponding to carbonyl and pure aromatic carbons, since this force field does not distinguish between those two atom types, while the hydrogen was designated as aromatic. However, the oxygen could potentially be assigned as a carbonyl/carboxyl (i.e. double-bonded) or an ester/ether (i.e. single-bonded) oxygen atom type (see discussion for TraPPE-UA above). The variation in oxygen choice can be seen in Figure S18(a) as AMBER-99-model-1 and AMBER-99-model-2, respectively. CHARMM-27 is similar in its choice of selection for oxygen but also distinguishes between C1 (carbonyl/carboxyl carbon) and C2/C3 (aromatic carbon) with different ε and σ values. Interestingly, whereas changing the assignment of the oxygen atoms from double-bonded to single-bonded atom types leads to lower amounts adsorbed in AMBER-99-model-2 (similar to what was observed for TraPPE-UA), it actually leads to an increase for CHARMM-27-model-2. The higher uptakes are due to the higher ε values and the stronger interactions for that atom type (see Table S2). Overall, however, the differences are not very significant, and are within the uncertainty arising from force field choice, as discussed in the results section of the paper.

OPLS-AA also allows for the distinction between the carbon atom types. C1 was described as a carboxylate/ester carbon double-bonded to oxygen, while C2/C3 and H were taken as benzene atom types. The OPLS-AA force field provided three options for oxygen with parameters for a carboxyl/ester double-bonded oxygen, a hydroxyl/ester single-bonded oxygen or an ether oxygen (OPLS-AA-model-1, 2 and 3, respectively). Again, the decrease in the predicted uptakes can be explained as a consequence of the lower ε values for the oxygen (Table S2), reducing interaction energy as we change from the carboxyl to the hydroxyl and to the ether oxygen atom type.

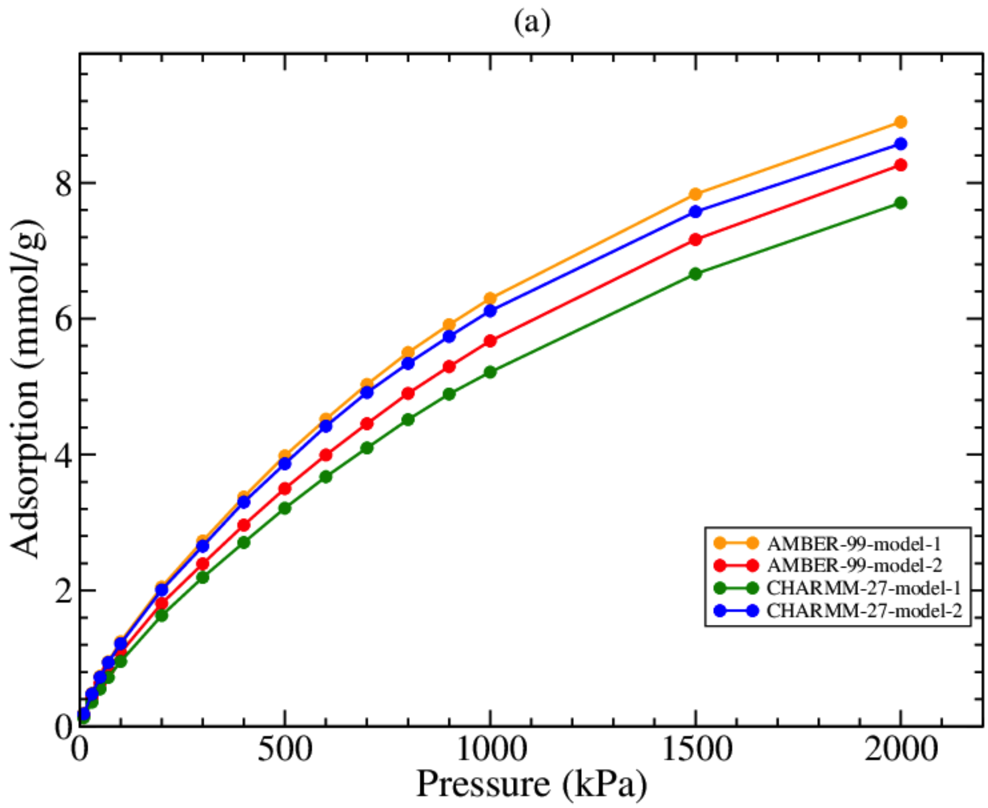

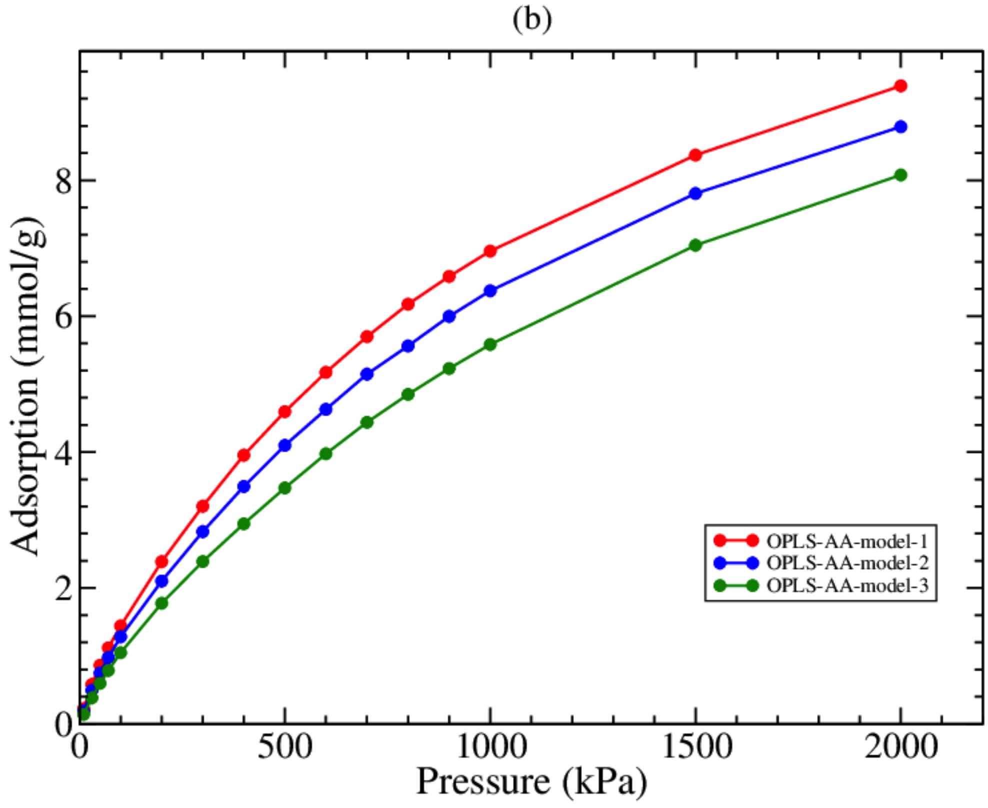

Figure S18. *Effect of different atom type assignments when modelling methane adsorption on Cu-BTC using (a) AMBER-99 and CHARMM-27; (b) OPLS-AA force fields (see text for a description of each parameter set).*

For the O2 oxygens that are only coordinatively bonded to two metal atoms in IRMOF-1 and MIL-47, we tested methane uptake in IRMOF-1 for force fields that distinguished between different types of oxygen atoms. AMBER-99 distinguishes between hydroxyl, carboxyl/phosphate group and ether/ester (single-bonded) oxygens. OPLS-AA is similar in its selections with alcohol, carboxylic acid/carboxylate/ester (double-bonded) and ether/ester (single-bonded) oxygens. CHARMM-27 only distinguishes between hydroxyl and carbonyl oxygens.

Figure S19 shows the effect of the atom type selection for O2 for methane adsorption on IRMOF-1 using the TraPPE-EH force field. It distinguishes between alcohol, ester (double-bonded) and ether/ester (single-bonded) oxygens – TraPPE-EH-model-1, 2 and 3, respectively (see Table S3). There is negligible distinction between the methane uptakes for the various oxygen atom type parameters. This was also true for the other force fields (AMBER, CHARMM and OPLS) although not shown here.

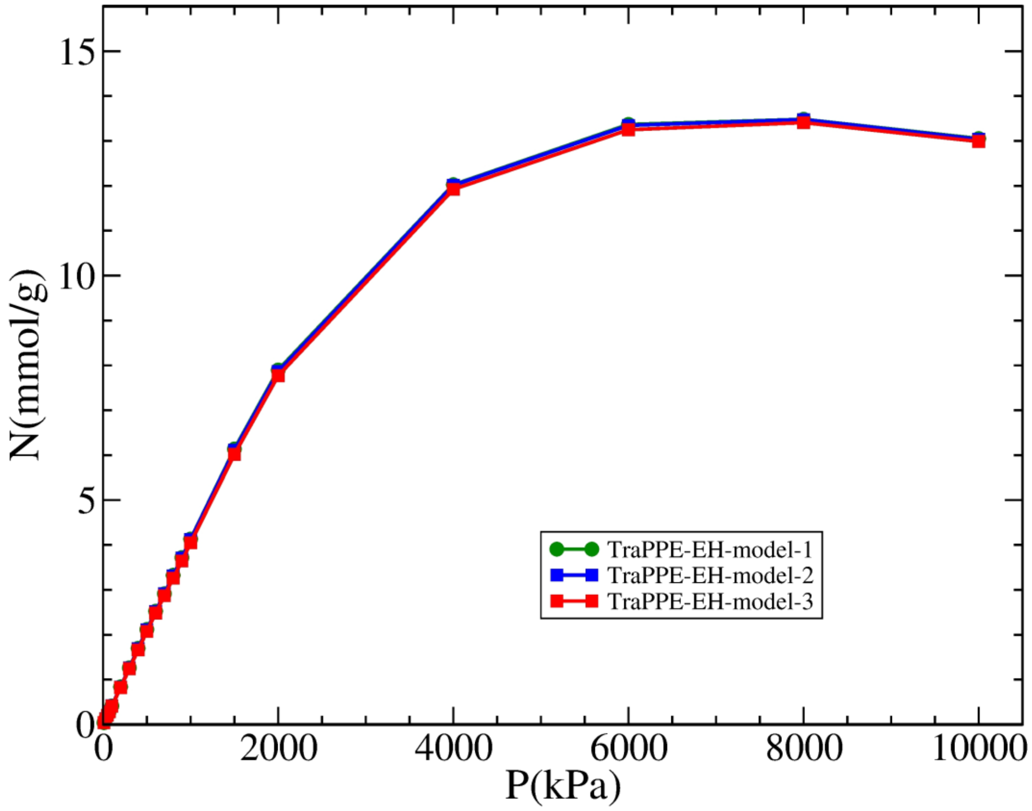

Figure S19. *Effect of different atom type assignments for O2 when modelling methane adsorption on IRMOF-1.*

This result suggests that these oxygen atoms are buried in the metal cluster and have no strong interactions with the adsorbate (methane); therefore, the parameter selection for this particular atom type was not a major factor in methane uptake. We decided to use the alcohol/hydroxyl group oxygen parameters since it was both available in all force fields (besides DREIDING and UFF) and it made the most chemical sense because the BDC linker in IRMOF-1 and MIL-47 comes from the reaction with terephthalic acid and a hydroxyl ion.[17]

$$4Zn^{2+} + 3H_2BDC + 8OH^{-} \rightarrow Zn_4O(BDC)_3 + 7H_2O$$

Tables S1-S3 report all the different sets of Lennard-Jones parameters from each force field (including variations of the final assignment reported in the main paper) used to model each of the 5 MOFs considered here, i.e. Cu-BTC, IRMOF-1, MIL-47, UiO-66 and Co-MOF-74.

***Table S1**. Lennard-Jones parameters used for modelling the Cu-BTC repeating unit in each TraPPE model tested here.*

Values for ε/k_B are in K and σ are given in Å. See Figure 3 of the main paper.

**Atom**	**TraPPE-EH**		**TraPPE-UA**		**TraPPE-UA-mod-1**		**TraPPE-UA-mod-2**		**TraPPE-UA-mod-3**	
	$\boldsymbol{\varepsilon/k_B}$	$\boldsymbol{\sigma}$	$\boldsymbol{\varepsilon/k_B}$	$\boldsymbol{\sigma}$	$\boldsymbol{\varepsilon/k_B}$	$\boldsymbol{\sigma}$	$\boldsymbol{\varepsilon/k_B}$	$\boldsymbol{\sigma}$	$\boldsymbol{\varepsilon/k_B}$	$\boldsymbol{\sigma}$
Cu[1]	2.516	3.114	2.516	3.114	2.516	3.114	2.516	3.114	2.516	3.114
O	79.0	3.050	79.0	3.050	55.0	2.800	55.0	2.800	79.0	3.050
C1	41.0	3.900	41.0	3.900	20.0	3.850	41.0	3.900	20.0	3.850
C2	30.7	3.600	21.0	3.880	21.0	3.880	21.0	3.880	21.0	3.880
C3	30.7	3.600	50.5	3.695	50.5	3.695	50.5	3.695	50.5	3.695
H	25.450	2.360	0.0	0.0	0.0	0.0	0.0	0.0	0.0	0.0

[1] – All Cu parameters were taken from UFF.

Table S2. *Lennard-Jones parameters used for modelling the Cu-BTC repeating unit in each AMBER, CHARMM and OPLS model tested here. Values for* ε/k_B *are in K and* σ *are given in Å. See Figure S18.*

**Atom**	**AMBER-99-model-1**		**AMBER-99-model-2**		**CHARMM-27-model-1**		**CHARMM-27-model-2**		**OPLS-AA-model-1**		**OPLS-AA-model-2**		**OPLS-AA-model-3**	
	ε/k_B	σ	ε/k_B	σ	ε/k_B	σ	ε/k_B	σ	ε/k_B	σ	ε/k_B	σ	ε/k_B	σ
Cu[1]	2.516	3.114	2.516	3.114	2.516	3.114	2.516	3.114	2.516	3.114	2.516	3.114	2.516	3.114
O	105.682	2.960	85.552	3.000	60.390	3.029	76.544	3.154	105.682	2.960	85.552	3.000	70.455	2.900
C1	43.279	3.400	43.279	3.400	55.357	3.564	55.357	3.564	52.841	3.750	52.841	3.750	52.841	3.750
C2	43.279	3.400	43.279	3.400	35.227	3.550	35.227	3.550	35.227	3.550	35.227	3.550	35.227	3.550
C3	43.279	3.400	43.279	3.400	35.227	3.550	35.227	3.550	35.227	3.550	35.227	3.550	35.227	3.550
H	7.549	2.600	7.549	2.600	15.097	2.420	15.097	2.420	15.097	2.420	15.097	2.420	15.097	2.420

[1] – All Cu parameters were taken from UFF.

Table S3. *Lennard-Jones parameters used for modelling the IRMOF-1 repeating unit in each TraPPE-EH model tested here for O2. Values for ε/k_B are in K and σ are given in Å. See Figure S19.*

**Atom**	**TraPPE-EH-model-1**		**TraPPE-EH-model-2**		**TraPPE-EH-model-3**	
	$\boldsymbol{\varepsilon/k_B}$	$\boldsymbol{\sigma}$	$\boldsymbol{\varepsilon/k_B}$	$\boldsymbol{\sigma}$	$\boldsymbol{\varepsilon/k_B}$	$\boldsymbol{\sigma}$
Zn*	62.399	2.462	62.399	2.462	62.399	2.462
O1	79.0	3.050	79.0	3.050	79.0	3.050
O2	93.0	3.020	79.0	3.050	55.0	2.800
C1	41.0	3.900	41.0	3.900	41.0	3.900
C2	30.7	3.600	21.0	3.880	30.7	3.600
C3	30.7	3.600	50.5	3.695	30.7	3.600
H	25.450	2.360	25.450	2.360	25.450	2.360

* – All Zn parameters were taken from UFF.

4. Detailed comparison between simulations and experiments for each MOF.

In what follows, we look at each of the selected MOF materials in turn, analysing the effect of varying the framework LJ force field and calculating a “consensus” simulated isotherm, which is then compared to the consensus experimental isotherm obtained from the data curation process. This allows us to rigorously assess the likelihood that simulations will lead to a good representation of experimental data and identify potentially problematic cases.

3.1 Cu-BTC

Figure S20a shows simulated adsorption isotherms for methane on Cu-BTC at 298 K using all 7 force fields considered here. As expected, different force fields predict different adsorption uptakes, with greater discrepancy in the intermediate pressure region. However, it is important to note that the curvature of all predicted isotherms is similar, suggesting that the adsorption mechanism is similar for all models considered. For this particular MOF, adsorption uptake increases in the order of DREIDING, UFF, TraPPE-UA, CHARMM-27, TraPPE-EH, AMBER-99 and OPLS-AA. The relatively large difference between OPLS-AA or AMBER-99, on the one hand, and CHARMM-27, on the other hand, is primarily due to the significant difference in the ε parameter for the oxygen atom (see Table 1), with the greater interaction energy resulting in higher predicted uptake of methane. In fact, the order of adsorption uptake roughly follows the order of increasing ε_{O1} parameter, suggesting that this particular atom plays an important role in the adsorption mechanism for Cu-BTC. It is also interesting to note that, despite the significant difference in values for the LJ parameters, DREIDING and UFF lead to practically identical isotherms. Both those isotherms are at the lower end of the spectrum, however, suggesting that those force fields might lead to a systematic underestimation of the adsorbed amount in the moderate to high-pressure region.

The average of these seven different models was taken to yield a consensus isotherm for the predicted adsorption uptake of methane (dashed line in Figure S20a), and this was used for comparison with the experimental consensus isotherm. To convey the uncertainty arising from the choice of model, we include error bars to represent a 95% confidence interval for the average of the simulated data. From all the force fields, TraPPE-EH, TraPPE-UA and CHARMM-27 predict adsorption isotherms that lie within the error bars of the consensus average.

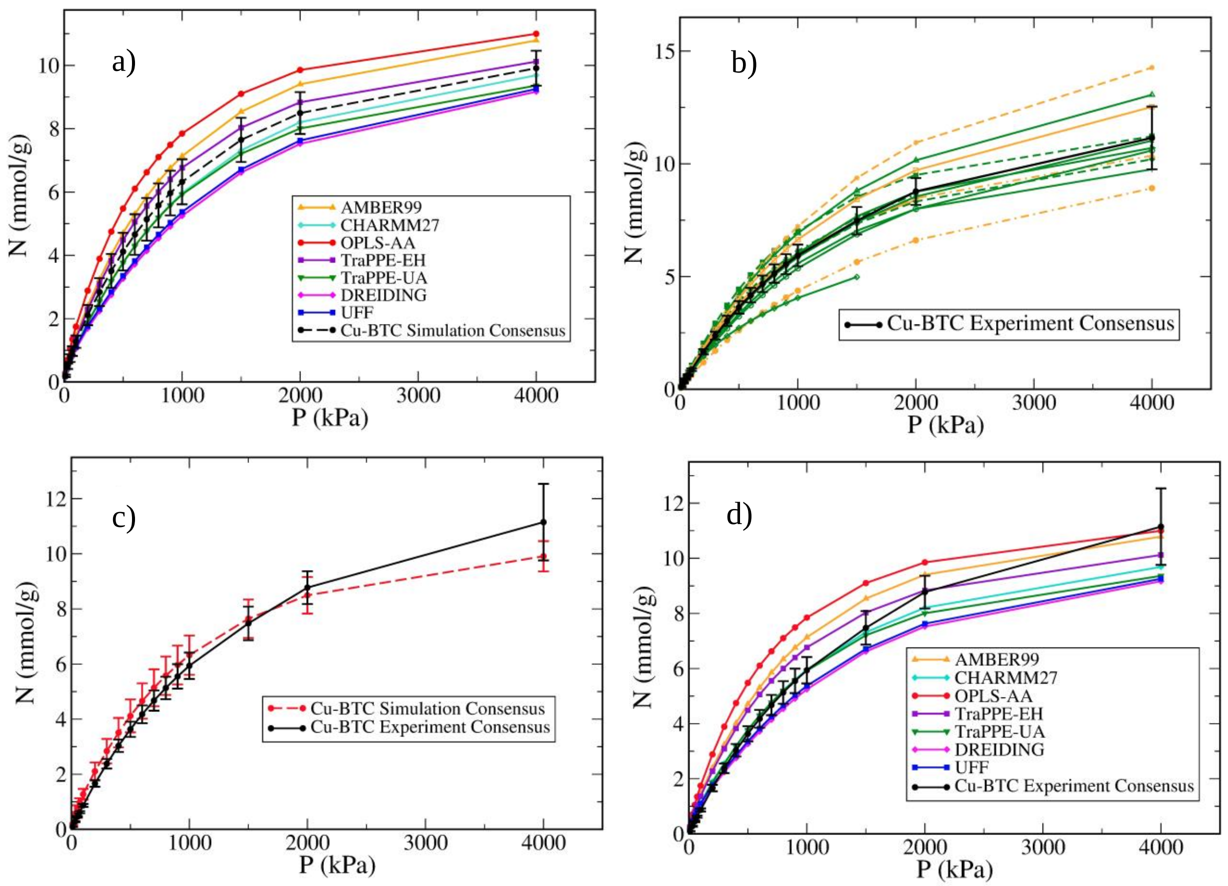

Figure S20. *Adsorption isotherms for methane on Cu-BTC at 298 K: a) Predicted adsorption uptake for different force field parameter sets, together with the average of the simulated isotherms with 95% confidence interval error bars (black circles and dashed line); b) Experimental isotherms after pore volume scaling and outlier removal, together with the experimental consensus isotherm with error bars (black line); c) Consensus isotherms for both simulated isotherms (red dashed line) and experimental data (full black line); d) Simulated isotherms for different force fields compared to the experimental consensus isotherm (full black line).*

The process of experimental data collection and curation for Cu-BTC was described in detail in Section 1. The initial NIST-ISODB search for methane (or CH_4) in Cu-BTC (or HKUST-1) with the "experiment" tag yielded 44 data sets. However, only 25 of those pertained to distinct methane isotherms measured experimentally at temperatures of 298 ± 5 K (the others were either simulated data or pertained to other adsorbates, like CO_2). After categorisation according to the criteria described in Section 1, there were 19 data sets that were green or amber and could be scaled by pore volume ratio (this accounts for 76% of the raw data sets). After fitting the scaled data to the

Toth isotherm model, 3 outliers were removed through the application of Tukey's method – accounting for 16% of the scalable data sets – and the consensus curve with 95% confidence interval error bars was computed (Figure S20b).

The consensus curve of the simulated isotherms shows very good agreement with that of the NIST-ISODB/experimental consensus for Cu-BTC (Figure S20c), with both isotherms within error bars of each other. One might argue that simulations somewhat underpredict adsorption above 2000 kPa but it is important to note that the variability (and hence uncertainty) of the experimental data in this range is substantially higher. It is also worth noting that the application of pore volume scaling brings the consensus experimental isotherm into better agreement with the simulation consensus curve – see Figure 4 of the main paper for a comparison between simulated data and the unscaled experimental consensus isotherm.

While Figure S20c shows that, *on average*, simulation predictions are consistent with experimental data, this does not imply the same conclusions for individual comparisons. For example, when comparing individual force field parameter sets to the experimental consensus curve (Figure S20d), both CHARMM-27 and TraPPE-UA yield excellent agreement with the average of the experimental data in the low to mid-pressure regions (i.e. up ~1500 kPa), but they start to underpredict adsorption above 2000 kPa and are below the lower limit of uncertainty in the experimental consensus isotherm at high pressures. Conversely, AMBER-99 and OPLS-AA fall well within the error bars of the experimental consensus isotherm at the highest pressure, but both significantly overpredict the methane uptake in the low to mid-pressure regions.

3.2 IRMOF-1

Figure S21a shows the predicted isotherms for methane on IRMOF-1 obtained from the different force fields, as well as the average isotherm. The variability is somewhat lower than for Cu-BTC, possibly because the curvature of the IRMOF-1 isotherm is less pronounced (i.e. it is much closer to linear). The order of methane uptake in IRMOF-1 is also slightly different from that in Cu-BTC, increasing according to DREIDING, TraPPE-UA, CHARMM-27, UFF, TraPPE-EH, AMBER-99 and OPLS-AA. In particular, UFF yields comparatively higher uptakes in IRMOF-1 than in Cu-BTC. In this case, all force field predictions except DREIDING (on the lower end) and OPLS-AA

(on the higher end) are within the 95% confidence interval of the consensus isotherm (black line with error bars).

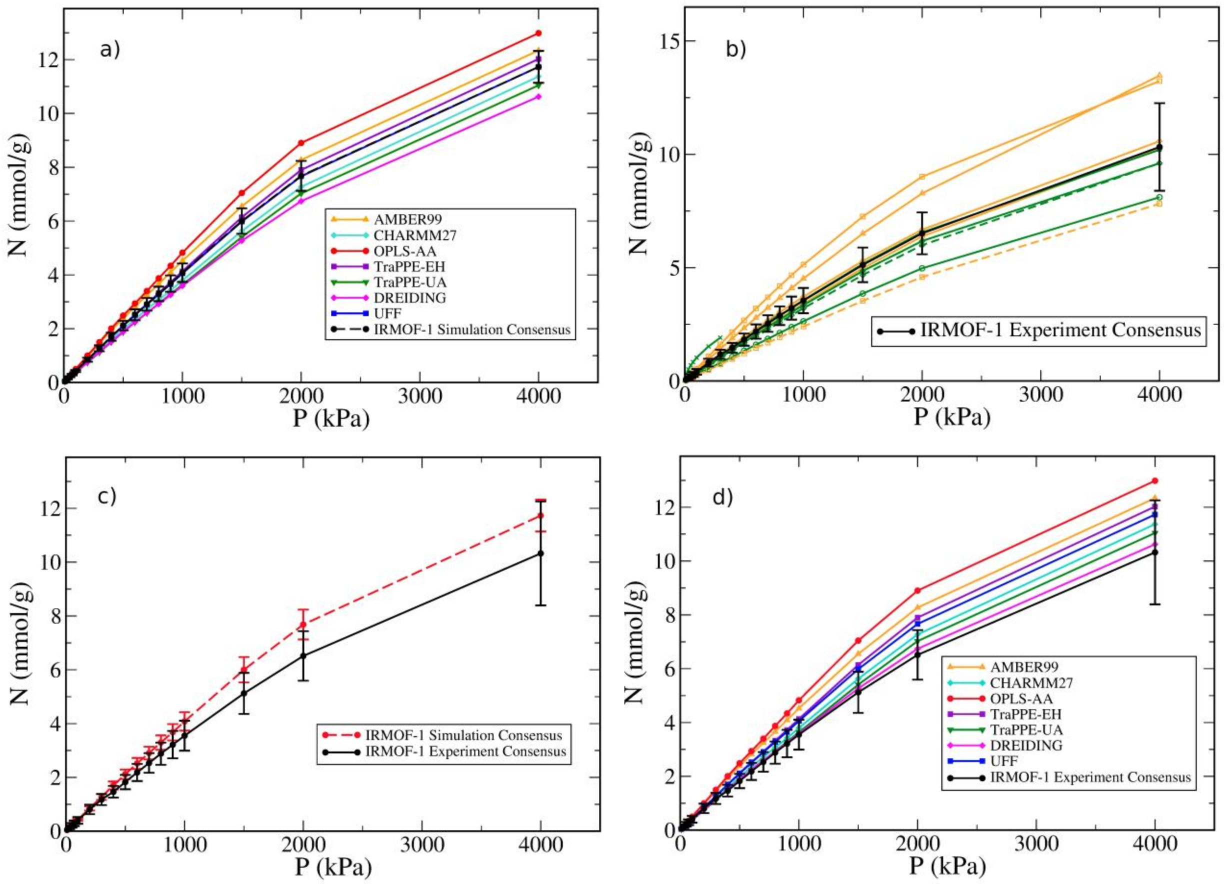

Figure S21. *Adsorption isotherms for methane on IRMOF-1 at 298 K: a) Predicted adsorption uptake for different force field parameter sets, together with the average of the simulated isotherms with 95% confidence interval error bars (black circles and dashed line); b) Experimental isotherms after pore volume scaling and outlier removal, together with the experimental consensus isotherm with error bars (black line); c) Consensus isotherms for both simulated isotherms (red dashed line) and experimental data (full black line); d) Simulated isotherms for different force fields compared to the experimental consensus isotherm (full black line).*

The initial NIST-ISODB search for methane (or CH_4) in IRMOF-1 (or MOF-5) with the "experiment" tag yielded 20 data sets but further searching resulted in 38 data sets available in NIST-ISODB. From these, only 9 pertained to distinct methane isotherms measured experimentally at temperatures within 298 ± 5 K. Therefore, an additional 6 data sets were found

through a literature search, yielding a total of 15 valid adsorption isotherms. After categorisation according to the criteria described in Section 1, there were 14 data sets that were green or amber and could be scaled by the pore volume ratio. After fitting the scaled data to the Toth isotherm model, 3 outliers were removed through the application of Tukey's method – accounting for 21.4% of the 14 scalable data sets – and the consensus curve with 95% confidence interval error bars was determined. These scaled isotherms, together with the consensus isotherm, are plotted in Figure S21b.

The simulated and experimental consensus isotherms show very good agreement with each other in the low-pressure region, with the simulation consensus slightly overpredicting the uptake for pressures above 500 kPa but well within the error bar range (Figure S21c). When comparing the individual force field parameter sets to the experimental consensus curve (Figure S21d), it is the DREIDING model that provides the best agreement with the average of the experimental data, although several other models (TraPPE-UA, CHARMM-27 and, to a lesser extent, TraPPE-EH and UFF) predict isotherms that are generally within the experimental error bars. The AMBER-99 and OPLS-AA models significantly overpredict the uptake of methane on IRMOF-1.

3.3 Co-MOF-74

The order of methane adsorption uptake in Co-MOF-74 increases according to UFF, DREIDING, CHARMM-27, TraPPE-UA, TraPPE-EH, AMBER-99 and OPLS-AA (Figure S22a). As for Cu-BTC, the curvature of the isotherm is quite pronounced, leading to a greater degree of variability in the intermediate pressure region. TraPPE-EH, TraPPE-UA and CHARMM-27 all show very good agreement with the simulation consensus, with the other four force fields lying beyond the 95% confidence interval across the entire pressure range – AMBER-99 and OPLS-AA above the error bars and UFF and DREIDING below.

The initial NIST-ISODB search for methane (or CH_4) in Co-MOF-74 (or Co-DODBC) with the "experiment" tag yielded only 3 data sets, one of which was actually a simulation, but a further literature search managed to uncover an additional 5 data sets. After checking the papers for pore volume and N_2 adsorption information, all 7 data sets were categorised as green or amber. After

fitting the scaled data to the Toth isotherm model, 2 outliers (37.5%) were removed and the consensus curve with 95% confidence interval error bars was determined (Figure 9b). The variability between experimental isotherms is noticeably smaller than observed for Cu-BTC and IRMOF-1, although the number of collected isotherms for Co-MOF-74 is also much smaller.

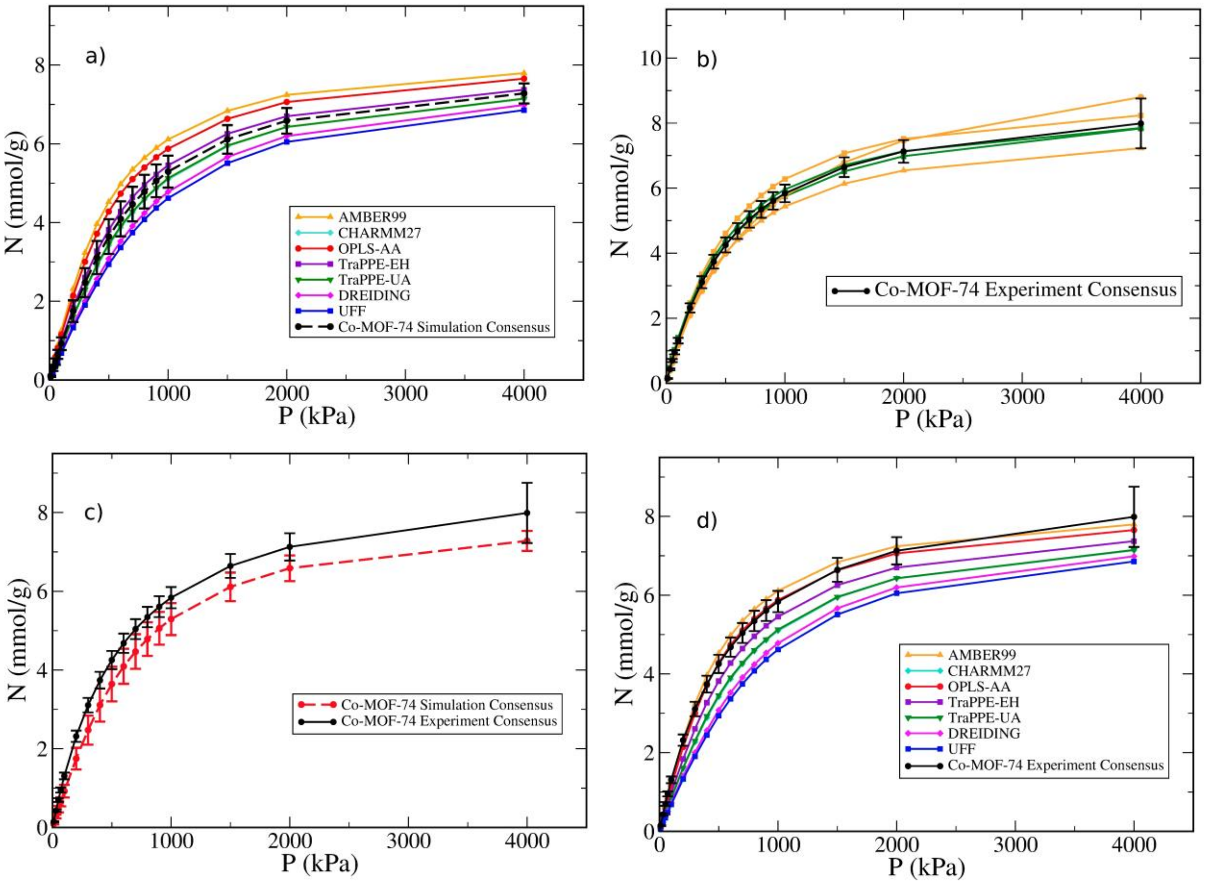

Figure S22. *Adsorption isotherms for methane on Co-MOF-74 at 298 K: a) Predicted adsorption uptake for different force field parameter sets, together with the average of the simulated isotherms with 95% confidence interval error bars (black circles and dashed line); b) Experimental isotherms after pore volume scaling and outlier removal, together with the experimental consensus isotherm with error bars (black line); c) Consensus isotherms for both simulated isotherms (red dashed line) and experimental data (full black line); d) Simulated isotherms for different force fields compared to the experimental consensus isotherm (full black line).*

There is good agreement between the simulated and experimental consensus curves (Figure S22c), although the simulated consensus slightly but systematically underpredicts the experimental

consensus over the whole pressure range. AMBER-99 and OPLS-AA are the individual force field models that most consistently agree with the experimental consensus (Figure S22d) since they yield stronger adsorbate-adsorbent interactions and hence predict higher adsorption uptake. All the other models underpredict the experimental methane uptake in Co-MOF-74, although TraPPE-EH yields the smallest discrepancy, falling just below the lower limit of the 95% confidence interval.

Like Cu-BTC, Co-MOF-74 also contains OMS, albeit at a much higher concentration. Therefore, one would expect that the effect of OMS interactions should be more significant in the latter, and this might explain the slight but systematic underestimation of experimental adsorption by standard force fields (see Figure S22c). However, given that the two consensus isotherms are in statistical agreement, a detailed analysis of this system is beyond the scope of the present paper.

3.4 MIL-47

The predicted methane uptake in MIL-47 at 298 K increases according to TraPPE-UA, CHARMM-27, DREIDING, TraPPE-EH, UFF, OPLS-AA and AMBER-99 (Figure S23a). The TraPPE-EH model provided the best agreement with simulation consensus throughout the entire pressure range, although most other FFs also mostly lie within the error bars. The exceptions are AMBER-99 (on the high end) and TraPPE-UA, which exhibit a significantly lower predicted methane uptake than the other six force fields. This trend is somewhat different than observed for the other MOFs, perhaps suggesting that explicit interactions with aromatic hydrogen atoms play a more important role in adsorption on MIL-47.

The initial NIST-ISODB search for methane (or CH_4) in MIL-47 with the "experiment" tag yielded 4 data sets, 2 of which were simulated isotherms. Searching again without the "experiment" tag yielded a further 5 results but none were experimental measurements. A further literature search uncovered an additional 4 data sets pertaining to distinct, experimentally measured methane isotherms at temperatures of 298 ± 5 K. Unfortunately, three of those sets did not report any pore volume information and were thus categorised as "red" and discarded from further analysis. This left us with only 3 green or amber isotherms, which were scaled and fitted. Those 3 isotherms showed a reasonable degree of consistency, and no outliers were detected by applying Tukey's

method. The consensus curve with a 95% confidence interval was determined and is shown in Figure S23b. It is worth noting that the three "red" isotherms are actually consistent with the consensus isotherm (see Figure S13), so including them in the analysis would have led to a reduction in the experimental uncertainty.

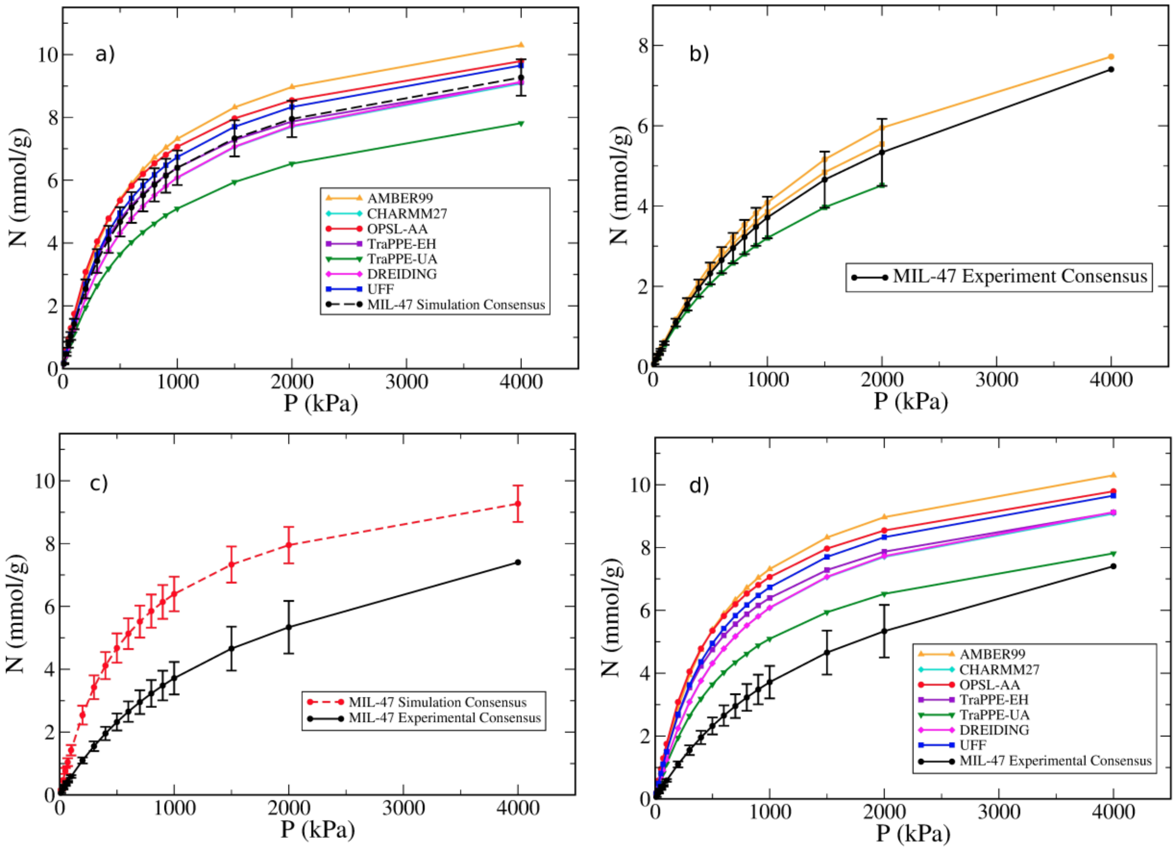

Figure S23. *Adsorption isotherms for methane on MIL-47 at 298 K: a) Predicted adsorption uptake for different force field parameter sets, together with the average of the simulated isotherms with 95% confidence interval error bars (black circles and dashed line); b) Experimental isotherms after pore volume scaling and outlier removal, together with the experimental consensus isotherm with error bars (black line); c) Consensus isotherms for both simulated isotherms (red dashed line) and experimental data (full black line); d) Simulated isotherms for different force fields compared to the experimental consensus isotherm (full black line).*

The simulation consensus isotherm greatly overpredicts the experimental methane uptake in MIL-47 (Figure S23c). Even when comparing the experimental consensus to individual models (Figure S23d), all force fields overpredict methane adsorption by a significant margin beyond the upper limit of the error bars. On the other hand, all except one of the experimental isotherms were reported by the same research grouping, suggesting that the experimental variability for MIL-47

may be underestimated. Further measurements of methane adsorption on this material by independent authors would be quite valuable. A more detailed discussion of the possible reasons for this discrepancy is presented in the main paper.

3.5 UiO-66

The predicted methane adsorption uptake in UiO-66 decreases in the rough order of AMBER-99, UFF, OPLS-AA, TraPPE-EH, TraPPE-UA, CHARMM-27, DREIDING, although the last four curves show a significant degree of overlap and the order changes depending on the pressure (Figure S24a). Only AMBER-99 (on the high end) and DREIDING (on the low end) consistently predict methane uptakes outside the 95% confidence intervals of the simulation consensus. The OPLS-AA model best agrees with the simulation consensus, followed by UFF.

The initial NIST-ISODB search for methane (or CH_4) in UiO-66 with the "experiment" tag yielded 11 data sets but 2 were simulated isotherms, one was measured at 273 K, one was measured at 308 K and one was a duplicate that referenced another source. A review of the literature managed to yield a further 29 valid data sets. Of the final 35 distinct experimental isotherms at temperatures of 298 ± 5 K, only one was discarded due to unavailability of pore volume information (i.e. categorised as "red"). However, the vast majority of remaining samples (29 out of 34, i.e. >85%) had experimental pore volumes greater than the theoretical pore volume and were therefore not scaled by the pore volume ratio (see Equation (1)). As such, unlike the other MOFs studied here, the pore volume scaling step had a negligible effect on the experimental consensus curve for UiO-66 (see Figure S15). Only two isotherms were identified as outliers to determine the consensus curve with 95% confidence intervals (Figure S24b).

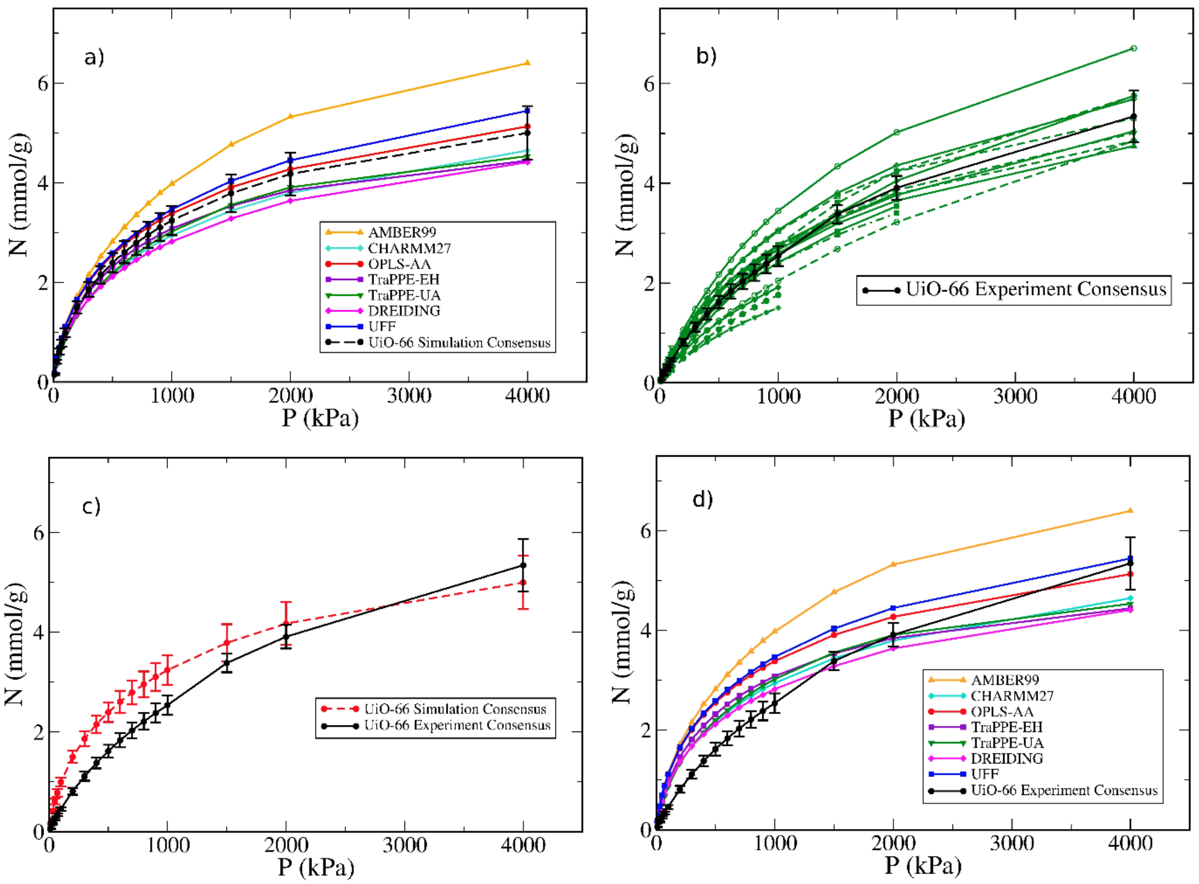

Figure S24. Adsorption isotherms for methane on UiO-66 at 298 K: a) Predicted adsorption uptake for different force field parameter sets, together with the average of the simulated isotherms with 95% confidence interval error bars (black circles and dashed line); b) Experimental isotherms after pore volume scaling and outlier removal, together with the experimental consensus isotherm with error bars (black line); c) Consensus isotherms for both simulated isotherms (red dashed line) and experimental data (full black line); d) Simulated isotherms for different force fields compared to the experimental consensus isotherm (full black line).

Compared to the experimental consensus, the simulation consensus overpredicts the methane uptake in UiO-66 over the low to mid-pressure regions (Figure S24c). The two isotherms cross over at ~2500 kPa, after which the simulation seems to underpredict experimental adsorption. However, at these high pressures, the uncertainty in the experimental data is higher and the two sets of error bars overlap to some extent. A similar trend is observed in Figure 12d, where the difference in the curvature between experimental and simulated isotherms becomes even more apparent.

5. Methane on MIL-47 Comparison with Models Found in Literature.

Liu *et al.*[18] used modified UFF LJ parameters (Table S4) for the organic linker and standard UFF for the vanadium metal atom to find good agreement with experimental data from Rosenbach *et al.*[19] Liu *et al.* state that they used a cut-off radius of 12.8 Å in their simulations, but do not clarify if this corresponds to a truncated or shifted potential. However, they cite an earlier study for details, which applied a shifted potential. In Figure S25, we compare three different protocols (the two mentioned previously, as well as our own protocol that applies tail corrections) against the original simulation data from Liu *et al.*, digitised from their paper. [18] As we can see, our results using a shifted potential agree almost exactly with those of Liu *et al.*, strongly suggesting that this was the protocol they used. As discussed above, this leads to much lower adsorbed amounts than if the "full" potential, including tail corrections, is applied.

Table S4*. Modified UFF parameters from Liu et al.*

**atom**	**σ (Å)**	**ε/k_B (K)**
V	2.801*	8.056*
O	3.12*	26.15**
C	3.43*	45.27**
H	2.57*	18.14**

*Taken from UFF of Rappé *et al.*[20] **Taken from Liu *et al.*[18]

The results of Liu *et al.*, as well as our repetition, agree quite well with the experimental isotherm of Rosenbach *et al.* This isotherm was considered in our experimental data analysis, but was discarded due to absence of pore volume information (see Figure S13). Nevertheless, the unscaled isotherm is consistent with the consensus experimental isotherm (black line with error bars in Figure S25). Furthermore, the isotherm of the Liu *et al*. model agrees with the experimental consensus isotherm, within statistical uncertainty. However, it is clear that the curvature of the two isotherms is different, potentially implying that the Liu *et al.* model would underpredict adsorption closer to saturation. More importantly, as demonstrated in the main paper, the modifications of Liu *et al.* are not transferable to other MOFs, even when they have the same organic linker.

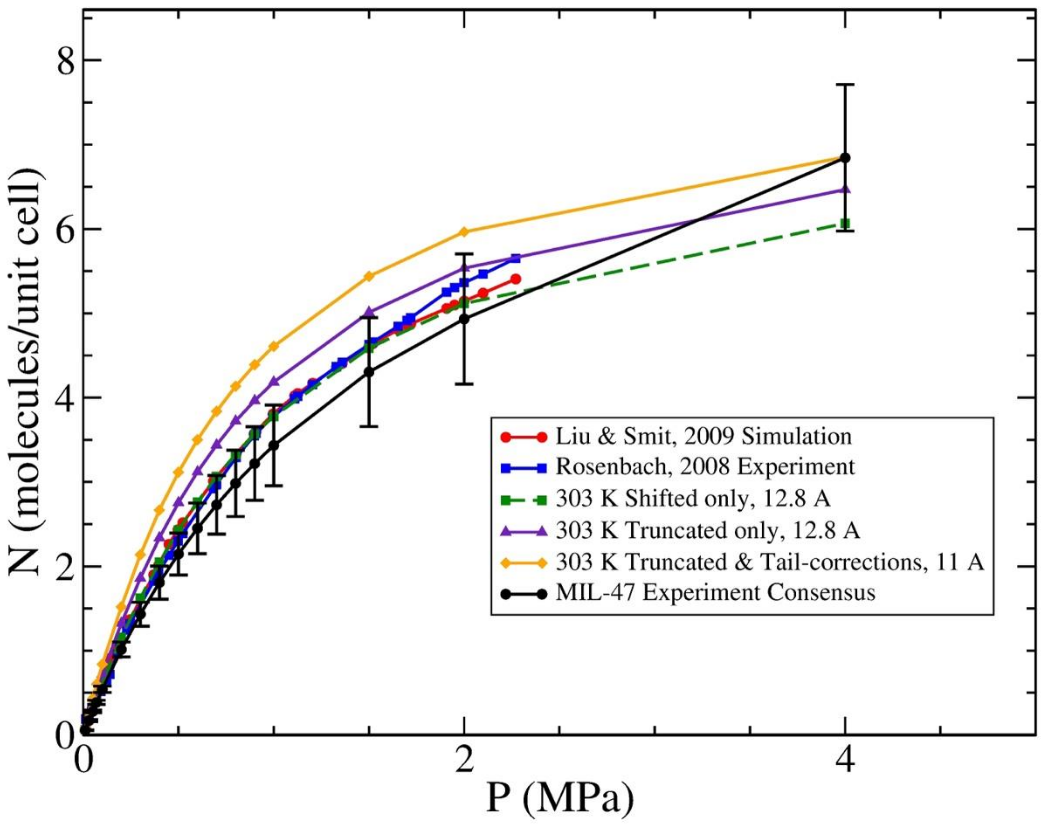

Figure S25. *Comparing the effect of shifted and/or truncated potentials and tail corrections on the methane adsorption on MIL-47 when using the modified UFF parameters from Liu et al.*

6. Impact of Defects on Methane Adsorption on UiO-66

Figure S26 compares adsorption isotherms obtained on different structures of UiO-66. First of all, there is very little difference between results obtained on the structure present in the RASPA repository, corresponding to the dehydroxylated structure and the hydroxylated structure provided by Van Speybroeck and co-workers (labelled "pristine"). Furthermore, the presence of up to 3 missing linkers has a practically negligible effect on the adsorption isotherm, in agreement with the results of Vandenbrande *et al*.[21] Therefore, in the main paper, we focused on the effect of missing clusters compared with the experimental consensus isotherm.

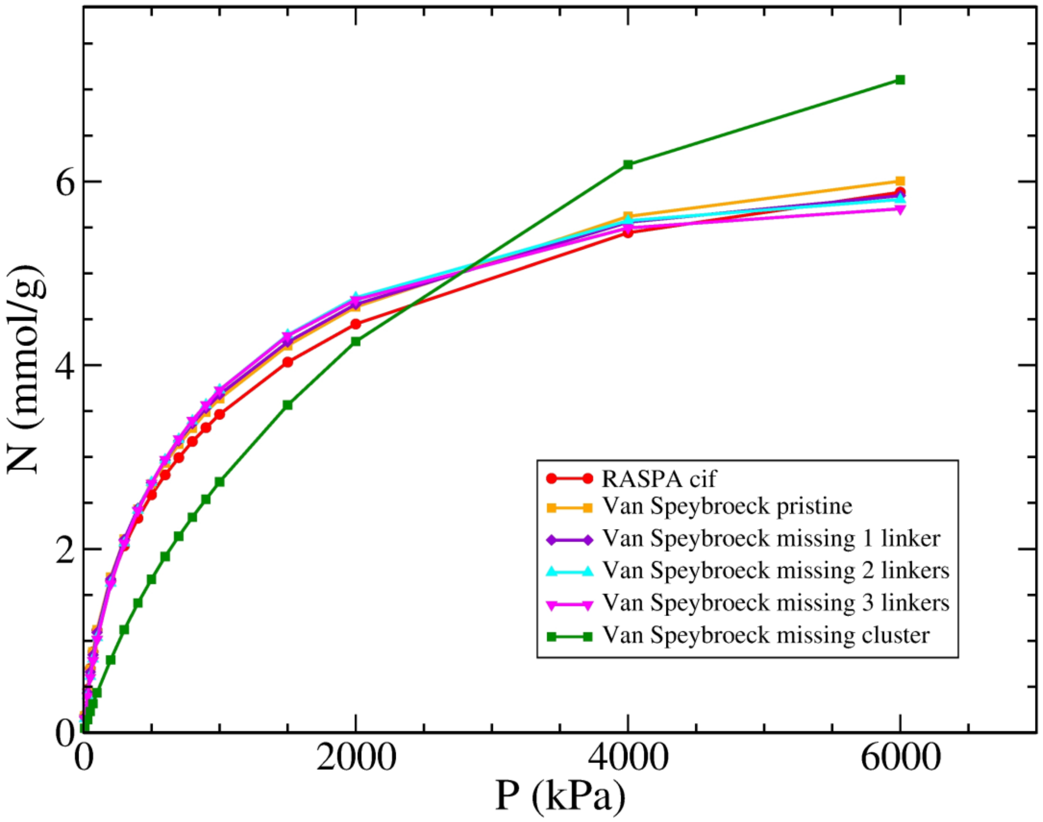

Figure S26. *Comparing methane adsorption on UiO-66 using the UFF force field: dehydroxylated (red circles), partially hydroxylated (orange squares), 1 missing linker (purple diamonds), 2 missing linkers (blue triangles up), 3 missing linkers (pink triangles down) and missing cluster (green squares).*

To model the UiO-66 pristine and missing cluster structures, we followed the same principles as the dehydroxylated structure. However, for the hydroxylated pristine UiO-66, we distinguished between the two oxygens in the Zr cluster since not all the oxygen atoms were bonded to a hydrogen atom (see Figure S27). These oxygen atoms were labelled as O2 (no H2 hydrogen) and O3 (with H2 hydrogen), so the O2 label remained consistent with that previously used. O3 was assigned the same parameters as O2 for all force fields since there were no chemical distinctions between them. Other than for DREIDING and UFF (generic oxygen parameters), both O2 and O3 were assigned as alcohol/hydroxyl atoms as done previously and, in all but one case, the H2 atom was given ε/k_B and σ values of zero, since the effect of the hydroxyl hydrogen atom type is implicitly included in the LJ parameters for the hydroxyl oxygen. Only CHARMM-27 provided an explicit parameter for this type of hydrogen (see Table S5).

The missing cluster structure follows the same labelling as the pristine structure apart from the additional atom type of C4 replacing C1 where the linker is cut off and terminated with a hydrogen atom, which we labelled as H3 (see Figure S28). For both TraPPE-EH and TraPPE-UA, the C4 had two options: i) continue with the same parameters as C1, leaving it assigned as a carboxyl; b) change C4 to an aldehyde CH united atom type that would include the terminating hydrogen. We decided to include both models in our analysis and determination of the simulation consensus isotherm.

AMBER-99 again does not distinguish between these carbon atoms, so all C1-C4 maintain the same parameters as a carbonyl/aromatic carbon. CHARMM-27 does not offer anything other than the same parameters used for C1. Therefore, C4 for CHARMM-27 is assigned as a carbonyl carbon. OPLS-AA does distinguish between C1 and C4 and provides an aldehyde atom type, but it has the exact same parameters as the carboxylate C1 (see Table S6).

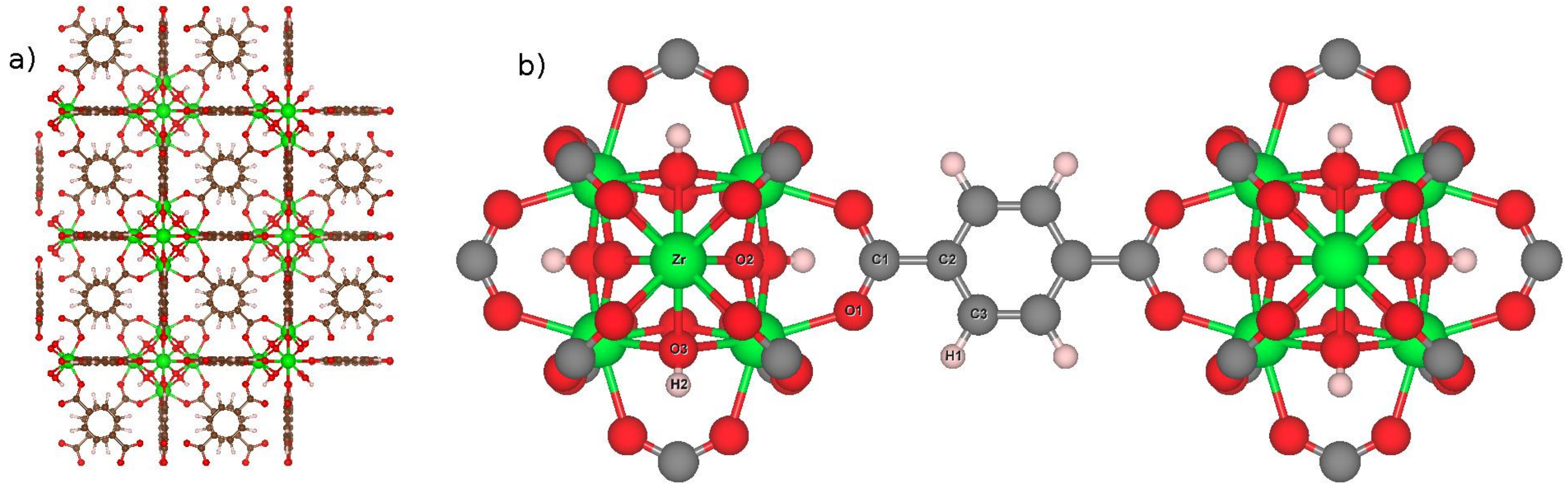

Figure S27. *UiO-66 hydroxylated pristine structure: a) unit cell showing no defects in the structure; b) repeat unit with labelled unique atom types.*

Table S5. *Lennard-Jones framework parameters used for modelling the UiO-66 pristine structure with each force field tested here.*[a]

Atom	UFF		DREIDING		TraPPE-EH		TraPPE-UA		AMBER-99		OPLS-AA		CHARMM-27	
	ε/k_B	σ	ε/k_B	σ	ε/k_B	σ	ε/k_B	σ	ε/k_B	σ	ε/k_B	σ	ε/k_B	σ
Zr[b]	34.722	2.783	34.722	2.783	34.722	2.783	34.722	2.783	34.722	2.783	34.722	2.783	34.722	2.783
O1	30.218	3.118	48.158	3.033	79.0	3.050	79.0	3.050	105.682	2.960	105.682	2.960	60.390	3.029
O2	30.193	3.118	48.158	3.033	93.0	3.020	93.0	3.020	105.883	3.067	85.552	3.070	76.544	3.154
O3	30.193	3.118	48.158	3.033	93.0	3.020	93.0	3.020	105.883	3.067	85.552	3.070	76.544	3.154
C1	52.838	3.431	47.845	3.473	41.0	3.900	41.0	3.900	43.279	3.400	52.841	3.750	55.357	3.564
C2	52.838	3.431	47.845	3.473	30.7	3.600	21.0	3.880	43.279	3.400	35.227	3.550	35.227	3.550
C3	52.838	3.431	47.845	3.473	30.7	3.600	50.5	3.695	43.279	3.400	35.227	3.550	35.227	3.550
H1	22.142	2.571	7.649	2.846	25.450	2.360	0.0	0.0	7.549	2.600	15.097	2.420	15.097	2.420
H2	22.142	2.571	7.649	2.846	0.0	0.0	0.0	0.0	0.0	0.0	0.0	0.0	23.148	0.400

[a] *Values for ε/k_B are in K and σ are given in Å.* [b] All parameters for metal atoms were taken from UFF.

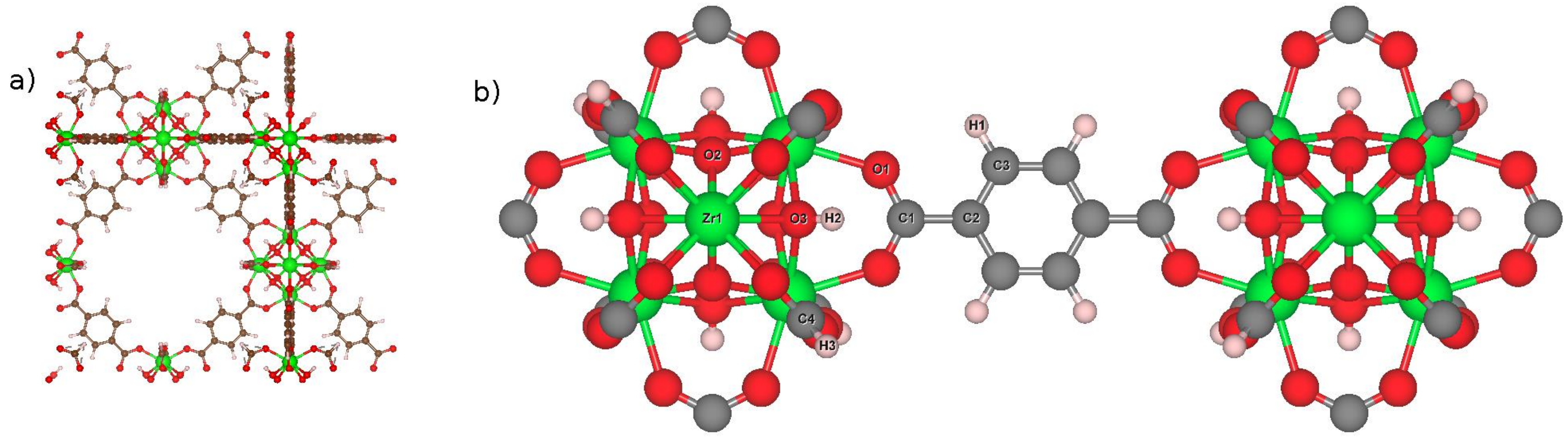

Figure S28. *UiO-66 with missing cluster structure: a) unit cell showing the defect in the structure; b) repeat unit with labelled unique atom types.*

Table S6. *Lennard-Jones framework parameters used for modelling the UiO-66 missing cluster structure with each force field tested here.*[a]

Atom	TraPPE-EH		TraPPE-EH aldehyde C4		TraPPE-UA		TraPPE-UA aldehyde C4		AMBER-99		OPLS-AA		CHARMM-27	
	ε/k_B	σ	ε/k_B	σ	ε/k_B	σ	ε/k_B	σ	ε/k_B	σ	ε/k_B	σ	ε/k_B	σ
Zr[b]	34.722	2.783	34.722	2.783	34.722	2.783	34.722	2.783	34.722	2.783	34.722	2.783	34.722	2.783
O1	79.0	3.050	79.0	3.050	79.0	3.050	79.0	3.050	105.682	2.960	105.682	2.960	60.390	3.029
O2	93.0	3.020	93.0	3.020	93.0	3.020	93.0	3.020	105.883	3.067	85.552	3.070	76.544	3.154
O3	93.0	3.020	93.0	3.020	93.0	3.020	93.0	3.020	105.883	3.067	85.552	3.070	76.544	3.154
C1	41.0	3.900	41.0	3.900	41.0	3.900	41.0	3.900	43.279	3.400	52.841	3.750	55.357	3.564
C2	30.7	3.600	30.7	3.600	21.0	3.880	21.0	3.880	43.279	3.400	35.227	3.550	35.227	3.550
C3	30.7	3.600	30.7	3.600	50.5	3.695	50.5	3.695	43.279	3.400	35.227	3.550	35.227	3.550
C4	30.7	3.600	54.0	3.520	21.0	3.880	54.0	3.520	43.279	3.400	52.841	3.750	55.357	3.564
H1	25.450	2.360	25.450	2.360	0.0	0.0	0.0	0.0	7.549	2.600	15.097	2.420	15.097	2.420
H2	0.0	0.0	0.0	0.0	0.0	0.0	0.0	0.0	0.0	0.0	0.0	0.0	23.148	0.400
H3	0.0	0.0	0.0	0.0	0.0	0.0	0.0	0.0	7.901	2.293	7.548	2.420	23.148	0.400

[a] *Values for ε/k_B are in K and σ are given in Å.* [b] All parameters for metal atoms were taken from UFF.

***Table S6 cont**. Lennard-Jones framework parameters used for modelling the UiO-66 missing cluster structure with each force field here.*[a]

Atom	UFF		DREIDING	
	ε/k_B	σ	ε/k_B	σ
Zr[b]	34.722	2.783	34.722	2.783
O1	30.218	3.118	48.158	3.033
O2	30.193	3.118	48.158	3.033
O3	30.193	3.118	48.158	3.033
C1	52.838	3.431	47.845	3.473
C2	52.838	3.431	47.845	3.473
C3	52.838	3.431	47.845	3.473
C4	52.838	3.431	47.846	3.473
H1	22.142	2.571	7.649	2.846
H2	22.142	2.571	7.649	2.846
H3	22.142	2.571	7.649	2.846

[a] *Values for ε/k_B are in K and σ are given in Å.* [b] All parameters for metal atoms were taken from UFF.

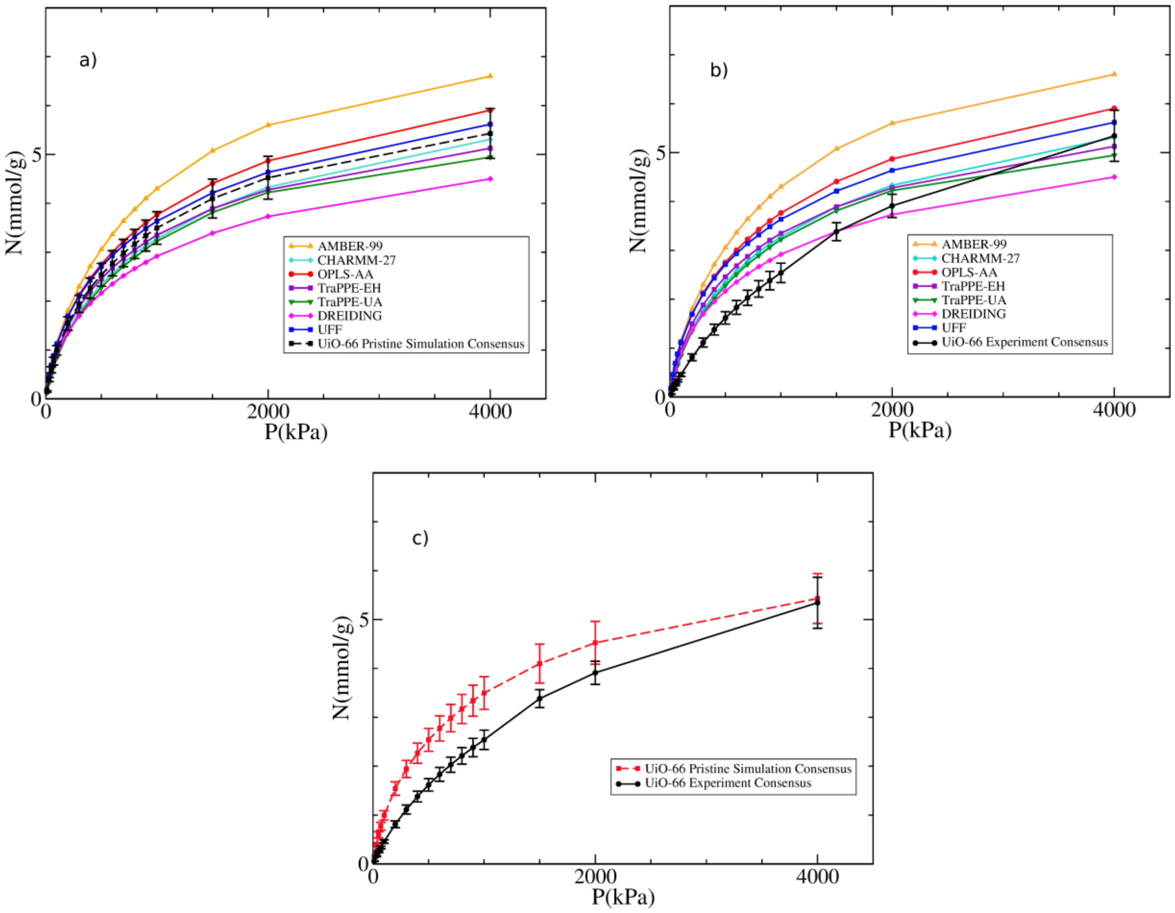

Figure S29. *Methane adsorption on UiO-66 pristine structure at 298 K: a) Predicted adsorption uptake for different force field parameter sets, together with the average of the simulated isotherms with 95% confidence interval error bars (black circles and dashed line); b) Simulated isotherms for different force fields compared to the experimental consensus isotherm (full black line); c) Consensus isotherms for both simulated isotherms (red dashed line) and experimental data (full black line).*

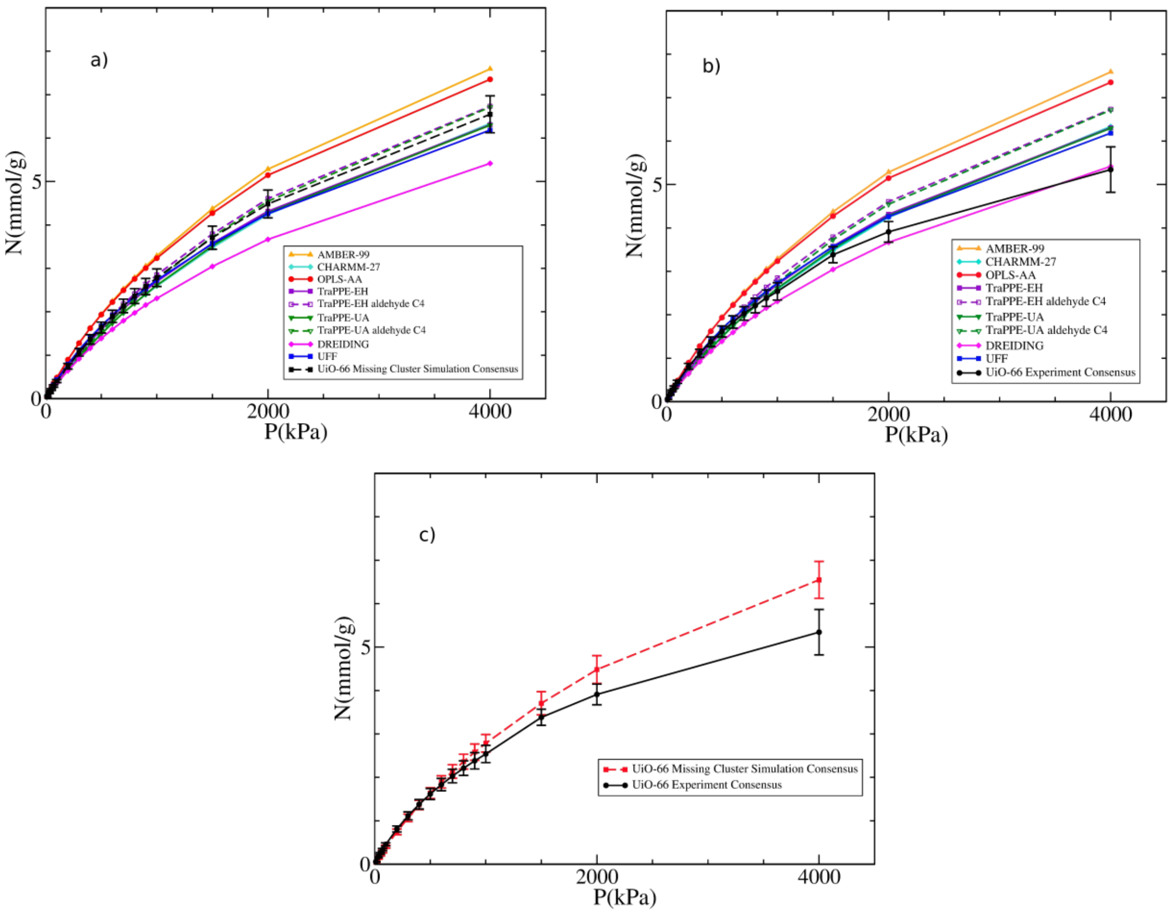

Figure S30. *Methane adsorption on UiO-66 missing cluster structure at 298 K: a) Predicted adsorption uptake for different force field parameter sets, together with the average of the simulated isotherms with 95% confidence interval error bars (black circles and dashed line); b) Simulated isotherms for different force fields compared to the experimental consensus isotherm (full black line); c) Consensus isotherms for both simulated isotherms (red dashed line) and experimental data (full black line).*

References

(1) Park, J.; Howe, J. D.; Sholl, D. S. How Reproducible Are Isotherm Measurements in Metal–Organic Frameworks? *Chemistry of Materials* **2017**, *29* (24), 10487–10495. https://doi.org/10.1021/acs.chemmater.7b04287.

(2) Siderius, D.; Shen, V.; Johnson III, R.; van Zee, R. NIST/ARPA-E Database of Novel and Emerging Adsorbent Materials (National Institute of Standards and Technology). 2020. https://doi.org/10.18434/T4/1502644.

(3) Surblé, S.; Millange, F.; Serre, C.; Düren, T.; Latroche, M.; Bourrelly, S.; Llewellyn, P. L.; Férey, G. Synthesis of MIL-102, a Chromium Carboxylate Metal−Organic Framework, with Gas Sorption Analysis. *J Am Chem Soc* **2006**, *128* (46), 14889–14896. https://doi.org/10.1021/ja064343u.

(4) Dubbeldam, D.; Frost, H.; Walton, K. S.; Snurr, R. Q. Molecular Simulation of Adsorption Sites of Light Gases in the Metal-Organic Framework IRMOF-1. *Fluid Phase Equilib* **2007**, *261* (1), 152–161. https://doi.org/https://doi.org/10.1016/j.fluid.2007.07.042.

(5) Demir, H.; Greathouse, J. A.; Staiger, C. L.; Perry IV, J. J.; Allendorf, M. D.; Sholl, D. S. DFT-Based Force Field Development for Noble Gas Adsorption in Metal Organic Frameworks. *J Mater Chem A Mater* **2015**, *3* (46), 23539–23548. https://doi.org/10.1039/C5TA06201B.

(6) Gurvitsch, L. J. Cited in SJ Gregg, KSW Sing, Adsorption, Surface Area and Porosity, Academic Press, London, P113, 1982. As. *J. Phys. Chem. Soc. Russ* **1915**, *47* (1), 49–56.

(7) Ongari, D.; Talirz, L.; Jablonka, K. M.; Siderius, D. W.; Smit, B. Data-Driven Matching of Experimental Crystal Structures and Gas Adsorption Isotherms of Metal–Organic Frameworks. *J Chem Eng Data* **2022**, *67* (7), 1743–1756. https://doi.org/10.1021/acs.jced.1c00958.

(8) Farmahini, A. H.; Limbada, K.; Sarkisov, L. Comment on the Applicability of the Gurvich Rule for Estimation of Pore Volume in Microporous Zeolites. *Adsorption* **2022**, *28* (5), 219–230. https://doi.org/10.1007/s10450-022-00364-w.

(9) Carné-Sánchez, A.; Stylianou, K. C.; Carbonell, C.; Naderi, M.; Imaz, I.; Maspoch, D. Protecting Metal–Organic Framework Crystals from Hydrolytic Degradation by Spray-Dry Encapsulating Them into Polystyrene Microspheres. *Advanced Materials* **2015**, *27* (5), 869–873. https://doi.org/https://doi.org/10.1002/adma.201403827.

(10) Sarkisov, L.; Bueno-Perez, R.; Sutharson, M.; Fairen-Jimenez, D. Materials Informatics with PoreBlazer v4.0 and the CSD MOF Database. *Chemistry of Materials* **2020**, *32* (23), 9849–9867. https://doi.org/10.1021/acs.chemmater.0c03575.

(11) Willems, T. F.; Rycroft, C. H.; Kazi, M.; Meza, J. C.; Haranczyk, M. Algorithms and Tools for High-Throughput Geometry-Based Analysis of Crystalline Porous Materials. *Microporous and Mesoporous Materials* **2012**, *149* (1), 134–141. https://doi.org/https://doi.org/10.1016/j.micromeso.2011.08.020.

(12) Pinheiro, M.; Martin, R. L.; Rycroft, C. H.; Haranczyk, M. High Accuracy Geometric Analysis of Crystalline Porous Materials. *CrystEngComm* **2013**, *15* (37), 7531–7538. https://doi.org/10.1039/C3CE41057A.

(13) Pinheiro, M.; Martin, R. L.; Rycroft, C. H.; Jones, A.; Iglesia, E.; Haranczyk, M. Characterization and Comparison of Pore Landscapes in Crystalline Porous Materials. *J Mol Graph Model* **2013**, *44*, 208–219. https://doi.org/https://doi.org/10.1016/j.jmgm.2013.05.007.

(14) Tóth, J. Uniform Interpretation of Gas/Solid Adsorption. *Adv Colloid Interface Sci* **1995**, *55*, 1–239. https://doi.org/https://doi.org/10.1016/0001-8686(94)00226-3.

(15) Tukey, J. W. *Exploratory Data Analysis*; Reading, MA, 1977; Vol. 2.

(16) Bingel, L. W.; Walton, K. S.; Sholl, D. S. Experimentally Verified Alkane Adsorption Isotherms in Nanoporous Materials from Literature Meta-Analysis. *J Chem Eng Data* **2022**, *67* (7), 1757–1764. https://doi.org/10.1021/acs.jced.1c00967.

(17) Rosi, N. L.; Eckert, J.; Eddaoudi, M.; Vodak, D. T.; Kim, J.; O'Keeffe, M.; Yaghi, O. M. Hydrogen Storage in Microporous Metal-Organic Frameworks. *Science (1979)* **2003**, *300* (5622), 1127–1129. https://doi.org/10.1126/science.1083440.

(18) Liu, B.; Smit, B. Comparative Molecular Simulation Study of CO2/N2 and CH4/N2 Separation in Zeolites and Metal−Organic Frameworks. *Langmuir* **2009**, *25* (10), 5918–5926. https://doi.org/10.1021/la900823d.

(19) Rosenbach Jr., N.; Jobic, H.; Ghoufi, A.; Salles, F.; Maurin, G.; Bourrelly, S.; Llewellyn, P. L.; Devic, T.; Serre, C.; Férey, G. Quasi-Elastic Neutron Scattering and Molecular Dynamics Study of Methane Diffusion in Metal Organic Frameworks MIL-47(V) and MIL-53(Cr). *Angewandte Chemie International Edition* **2008**, *47* (35), 6611–6615. https://doi.org/https://doi.org/10.1002/anie.200801748.

(20) Rappé, A. K.; Casewit, C. J.; Colwell, K. S.; Goddard, W. A.; Skiff, W. M. UFF, a Full Periodic Table Force Field for Molecular Mechanics and Molecular Dynamics Simulations. *J Am Chem Soc* **1992**, *114* (25), 10024–10035. https://doi.org/10.1021/ja00051a040.

(21) Vandenbrande, S.; Verstraelen, T.; Gutiérrez-Sevillano, J. J.; Waroquier, M.; Van Speybroeck, V. Methane Adsorption in Zr-Based MOFs: Comparison and Critical Evaluation of Force Fields. *The Journal of Physical Chemistry C* **2017**, *121* (45), 25309–25322. https://doi.org/10.1021/acs.jpcc.7b08971.
